# Assessing the diet and seed dispersal ability of non‐native sambar deer (*Rusa unicolor*) in native ecosystems of south‐eastern Australia

**DOI:** 10.1002/ece3.10711

**Published:** 2023-11-27

**Authors:** Matthew J. Quin, John W. Morgan, Nicholas P. Murphy

**Affiliations:** ^1^ Department of Ecology, Environment and Evolution La Trobe University Bundoora Victoria Australia; ^2^ College of Science and Engineering James Cook University Douglas Queensland Australia; ^3^ Research Centre for Applied Alpine Ecology La Trobe University Bundoora Victoria Australia

**Keywords:** alpine, DNA sequencing, endozoochory, faecal DNA, germination, invasive plant species, native plant species, wet forest

## Abstract

Understanding the influence of non‐native herbivores on ecosystems by means of dietary foraging and seed dispersal is important for understanding how non‐native species can alter an invaded landscape, yet requires multiple methodologies. In south‐eastern Australia, introduced sambar deer (*Rusa unicolor*) are rapidly expanding in range and placing native ecosystems at risk through browsing and as vectors for seed dispersal. We simultaneously investigated sambar deer dietary composition and seed dispersal using DNA sequencing and germination trials, from faecal pellets collected in alpine and wet forest ecosystems. This allowed us to contrast the dietary impacts of introduced sambar deer in different environments, and to explore the potential for habitat‐specific variation in diet. DNA sequencing of the *trnL*, ITS2 and *rbcL* gene regions revealed a diverse plant species dietary composition comprising 1003 operational taxonomic units (OTUs). Sambar deer exhibited intermediate feeder behaviours dominated by forbs in alpine and shrubs in wet forest ecosystems. A large proportion of plant OTUs were considered likely to be native, however, the proportion of exotic species in the diet in both ecosystems was greater than would be expected based on the proportion of exotic species in each of the two landscapes. Seed germination trials indicated that sambar deer can disperse a substantial number of native and exotic species in both alpine and wet forest ecosystems. In alpine ecosystems, an individual sambar deer was estimated to disperse on average 816 (±193) seeds per day during the study period, of which 652 (±176) were exotic. *Synthesis and applications*. Our results suggest that native plant species comprise the majority of sambar deer diets in Australian ecosystems and that the introduced species is dispersing both native and exotic plant species via endozoochory. However, exotic species seedling germination numbers were significantly higher in alpine ecosystems, and given the large daily movements of sambar deer, represents a significant vector for the spread of exotic plant species. Management of native plant species and vegetation communities of conservation significance, or at risk to sambar deer browsing is of high priority, through either the removal of sambar deer or implementation of exclusion‐based methods.

## Graphical abstract

Understanding the influence of non‐native herbivores on ecosystems through dietary foraging and seed dispersal requires multiple methodologies. In south‐eastern Australia, sambar deer (*Rusa unicolor*) are rapidly expanding in range and placing native ecosystems at risk through browsing and as vectors for seed dispersal. We simultaneously investigated sambar deer dietary composition and seed dispersal using germination trials and DNA sequencing from faecal pellets collected in alpine and wet forest ecosystems. Our results suggest that native plant species comprise the majority of sambar deer diets in Australian ecosystems and that the species is dispersing both native and exotic plant species via endozoochory.

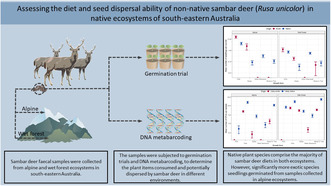

## 1 INTRODUCTION

The introduction and colonisation of non‐native species into new environments can have direct negative impacts on native species, and pose serious threats to native ecosystem function (Mack et al., 2000; Spear & Chown, 2009). Australian landscapes evolved in the absence of hard‐hoofed mammals (Pickering et al., 2010), yet many non‐native ungulates have colonised successfully since European settlement, including six species of deer (Davis et al., 2016; Moriarty, 2004). Currently, evidence suggests that three deer species, fallow (*Dama dama*), red (*Cervus elaphus*) and sambar (*Rusa unicolor*), are significantly increasing both their population size and distribution in the south‐eastern area of the continent (Davis et al., 2016).

Deer can alter the structure and function of ecosystems through browsing, grazing and trampling (Rooney & Waller, 2003). In areas where native herbivores share habitat and resources with deer, competition for resources and territory is high (Forsyth & Davis, 2011), potentially threatening the persistence of some native fauna (Côté et al., 2004). In some ecosystems, deer have shown selective feeding strategies, browsing certain plants more heavily than others and, in forested ecosystems, cascade effects on biodiversity have been observed following reductions in both canopy and herb layers through deer activity (Dolman & Wäber, 2008; Gill, 1992). Declines in songbird abundance have been associated with increasing deer populations in deciduous forests across Europe and North America, as many songbird species are dependent on understory vegetation, and are especially exposed to the reductions in vegetation density caused by deer (Cardinal et al., 2012; Chollet et al., 2015; Gill & Fuller, 2007). In Australia, the diet of deer is poorly understood (Davis et al., 2016), and a detailed investigation is necessary to understand dietary selection and potential impacts on native plant species.

Many studies for estimating herbivore diet have relied on microhistological and macroscopic techniques (Forsyth & Davis, 2011; Shrestha et al., 2005; Suter et al., 2004), which involve sampling stomach contents and identifying plant material ingested based on morphological and cell structural properties (Nugent, 1983). However, these invasive methods require taxonomic expertise to distinguish plant species, and have the potential for both over‐ and under‐estimation of dietary items due to differences in digestion rates (Kessler et al., 1981; Stribling et al., 2008). Recent advances in genetic techniques have demonstrated that DNA metabarcoding of faecal pellets provides a fast and efficient alternative for the investigation of herbivore diets (Creer et al., 2016; Goldberg et al., 2020).

Browsing of plant species by deer is also likely to influence seed dispersal (Bartuszevige & Endress, 2008). In Australia, hog deer (*Axis porcinus*) populations in the south‐east are capable of dispersing > 133,000 viable seeds per day via endozoochory, of which a majority are native (Davis et al., 2010). Claridge et al. (2016) compared the viable seed from faecal pellets of fallow deer and the native eastern grey kangaroo (*Macropus giganteus*) and determined that both a greater number of plant species and higher contribution of non‐native species germinated from the faecal pellets of fallow deer. Considering the longer stomach passage times attributed to deer, in combination with high levels of daily movement and consumption of a wide range of plant materials, endozoochory by deer has the potential to transport plant seeds long distances (Gill & Beardall, 2001).

In this study, we focus on sambar deer which are native to India, southern China and south‐eastern Asia, and have been successfully established in the United States, New Zealand and Australia (Leslie, 2011). Since their introduction into Australia in the 1860s, sambar deer have become well established in habitats ranging from coastal to alpine summits (Davis et al., 2016; Peel et al., 2005). The diet of sambar deer is considered varied, with season, location, habitat variety, competition and human activities all playing a role in dietary selection (Kushwaha et al., 2004), although forage availability appears to be a primary driver (Geist, 1998). In many cases, sambar deer display intermediate feeder behaviour, with the capacity to adapt to diets comprised heavily of browse or graze material (Leslie, 2011). Considering that the distribution of sambar deer encompasses a variety of ecosystems across south‐eastern Australia, we investigated the herbivory and the capacity for endozoochoric seed dispersal of sambar deer in two contrasting habitats; alpine and wet forests, which each contain diverse vegetative properties, and support varying densities of sambar deer. We used sambar deer faecal pellets to explore diet through DNA analyses and undertook germination trials to determine the plant species capable of germinating from faecal pellets. Our aim was to determine the diversity of native and introduced plant species eaten and spread by sambar deer, and how this may differ in two different ecosystems.

## 2 MATERIALS AND METHODS

### 2.1 Study area

#### 2.1.1 Alpine

The Bogong High Plains is an extensive area of high mountain vegetation situated in the Alpine National Park (36°53′ S, 147°18′ E) in north‐east Victoria, Australia. Located on a series of undulating alpine and subalpine plateaus (1660–1880 m above sea level; McDougall, 2003), this study area comprised a mixture of tussock grasslands, snow patch herbfields, open heathlands and snow gum (*Eucalyptus pauciflora*) dominated woodlands. Mean annual precipitation in the area is 2430 mm (much of which falls as snow in winter), with mean annual minimum and maximum temperatures of 2.7 and 9.5°C, respectively.

#### 2.1.2 Wet forest

The Central Highlands study area comprised the western sections of the Yarra Ranges National Park, in eastern Victoria (37°41′ S, 145°34′ E). This study area consisted of densely vegetated wet, damp and riparian forests dominated by mountain ash (*Eucalyptus regnans*) with shrubs, grasses and ferns comprising the understorey. The topography was varied with elevations from 200 to 1200 m, and the mean annual rainfall in the area is 1081 mm, with mean annual minimum and maximum temperatures of 7.5 and 20.5°C, respectively.

### 2.2 Sample collection

Sambar deer faecal pellets were collected from both study areas for DNA analysis and germination trials during Autumn 2019, after most seeds had set but before autumn seed germination. Sambar deer are the most common deer species inside the national parks where the study took place, and are likely to be the only deer species present in the specific locations where sampling occurred (Comte et al., 2022; Wills et al., 2023). As sambar deer expel many faecal pellets in one defecation, we define a faecal pellet group as a collection of pellets that varies in the total number of individual units yet are produced by only one sambar deer. A total of 160 sambar deer faecal pellet groups were collected across the two study areas (alpine; *n* = 81, wet forest; *n* = 79). Only fresh samples were collected, described as those that exhibited a wet shine with no outer decay. Sambar deer faecal pellets are easily distinguished from other mammals in Australia, however, a deer‐specific field guide was used to validate all sample collections (Claridge, 2016; Sotorra et al., 2021). Pellet groups displaying signs of contamination from non‐food items such as leaf or plant tissue were ignored.

Faecal pellets for DNA analysis were placed in individual plastic zip‐lock bags with silica gel sachets to limit moisture accumulation and stored at −30°C. Faecal pellets for germination trials were stored in individual paper bags and kept in a dark environment at room temperature until commencing the germination trial.

### 2.3 DNA extraction

Forty faecal pellet samples (alpine; *n* = 20, wet forest; *n* = 20) were selected for DNA analysis. Each sample comprised 8–12 faecal pellets collected in the field and were homogenised into single samples. Three subsamples of ~200 mg of each homogenised sample were placed into separate microcentrifuge tubes for DNA extraction. Extractions were undertaken in a dedicated polymerase chain reaction (PCR) free room using a QIAamp Fast DNA Stool Mini Kit (Qiagen) following manufacturer's protocols. Alpine and wet forest samples were extracted in different sessions to avoid contamination, and negative extraction controls were performed throughout.

### 2.4 PCR amplification and library preparation

Established primer sets were used to amplify segments of the *rbcL*, ITS2 and *trnL* intron P6 loop gene regions, following custom PCR reactions and protocols (Appendix S1). PCRs included both negative extraction and H_2_O controls to test for sample contamination. After successful amplification of target gene regions, an additional PCR was performed to attach Illumina adaptors and dual indexes. All PCR products were cleaned and normalised, and a final library comprising alpine and wet forest samples was prepared as per the Illumina MiSeq protocol. Sequencing was performed using MiSeq Reagent v2 (2 × 250 bp) sequencing kit.

### 2.5 Data filtering and analysis

The sequence analysis tool USEARCH v11.0.667 (Edgar, 2010) was used to cluster sequences into operational taxonomic units (OTUs) at ≥97% similarity, as this corresponds to approximately species level (Schloss & Handelsman, 2005; Appendix S1). The frequency of OTUs across samples from each gene region was examined and compared between study areas.

To assess the feeding behaviour of sambar deer in both ecosystems, OTUs were categorised as one of five plant growth forms (fern, forb, grass, other graminoid, shrub or tree). OTUs classified only to genus level were categorised based on the proportion of species of a particular growth form within the genus, and the presence of these species within each study area. The presence of species within the study area was based on records retrieved from the online Royal Botanic Gardens Victoria VicFlora database. We then compared the OTU richness for each growth form using a Kruskal–Wallis *H* test with post hoc Dunn's test in R (R Development Core Team, 2010). To validate this approach, and ensure we were not overlooking important within‐genus variation, we also performed this same test using a subset of the data containing only OTUs determined to species level.

To determine the proportion of native and exotic OTUs within the sambar deer diet, the datasets for each gene region were combined and duplicate species were removed. OTUs were labelled as ‘Likely Native’, ‘Likely Exotic’ or ‘Unknown’ by comparison to the ‘Establishment Means’ information on the online Royal Botanic Gardens Victoria VicFlora database. Any OTUs identified only to genus were classified based on the proportion of native‐to‐exotic species contained within the genus, and the presence of these species within each study area. The online VicFlora database was used once again to confirm the presence of species within the study area. A genus was labelled ‘Likely Native’ if more than 50% of the species within the genus in the study area were native. The ‘Unknown’ characterisation was used for OTUs that were identified only to family, as well as for genera that were split with 50% native and 50% exotic species within the sampled region. The proportional native and exotic richness of sambar deer diet was assessed with a two‐proportion z‐test, comparing the proportions of native and exotic OTUs with the expected proportions within each study area. We performed this same test on a subset of the data containing only species‐level classifications of native and exotic origin to assess the similarity of patterns when comparing to broader genus‐level classifications.

### 2.6 Seed germination trials

For many alpine plant species, a period of snow cover is required to break seed dormancy and promote germination (Hoyle et al., 2014). Therefore, a cold–wet stratification technique was applied to the alpine samples. Alpine faecal pellet samples were halved by weight, with half placed at room temperature in a dark environment, while the other half were dampened and placed in a cool room at 2°C to undergo the cold–wet stratification. During this period, wet forest samples were stored at room temperature in a dark environment. All samples were removed and prepared for the germination trial after 35 days.

A total of 256 punnets (85 × 135 × 50 mm) were prepared with a 4:1 mixture of sterilised seed‐raising mix to vermiculite. Faecal pellet samples were crumbled into coarse fragments within individual paper bags and evenly spread over the seed‐raising mix (layer of approximately 5 mm). The 81 alpine pellet group samples were divided to create matched pairs of cold–wet stratified and non‐stratified samples (*n* = 162 in total). Seventy‐nine wet forest samples and 15 control punnets (containing only seed‐raising mix and vermiculite) were randomly distributed within an unheated glasshouse. Trays were illuminated with natural light during the day and were watered ad libitum. All trays were randomly redistributed once a week to avoid within‐glasshouse microclimatic differences, and ‘control’ punnets were randomly distributed within trays to record potential contamination by wind‐blown seed. After germination, seedlings were identified, counted and removed from punnets. Unknown species were transferred to individual pots and grown further until they could be identified. The germination study was run for 365 days.

To determine whether the non‐stratified and cold–wet stratified alpine datasets should be analysed independently, a Spearman's rank correlation coefficient test was used to test for correlation of the number of germinants per punnet, number of different species per punnet and number of germinants for each different species. A non‐parametric Wilcoxon rank‐sum test was used to compare the number of native and exotic seedlings that emerged from sambar deer faecal pellets in both study regions, and the same test was used to compare the mean plant species richness per punnet between the alpine and wet forest samples. To test for differences in the plant growth form of emerging seedlings (forb, grass, other graminoid, shrub or tree), a Kruskal–Wallis *H* test with post hoc Dunn's test was performed.

The potential number of viable seeds dispersed daily by an individual sambar deer was estimated following previous methodologies (Williams et al., 2008). The mean number of germinants for a complete sambar deer defecation was determined by multiplying the mean number of germinants from the 12 collected pellets in this study by 2.98, based on a sambar deer pellet group averaging 35.8 pellets (Sotorra et al., 2021), and then multiplying this number by an estimated defecation rate of 12 pellet groups per day (Srikosamatara, 1993).

## 3 RESULTS

### 3.1 DNA sequencing

A total of 5,988,518 filtered reads were generated, comprising 2,949,158 from alpine samples and 3,039,630 from wet forest samples. Data analysis resulted in a total of 138 alpine and 227 wet forest plant OTUs for the *trnL* gene region, 123 alpine and 221 wet forest plant OTUs for the ITS2 gene region and 79 alpine and 215 wet forest plant OTUs using the *rbcL* gene region (Appendix S1).

**FIGURE 1:**
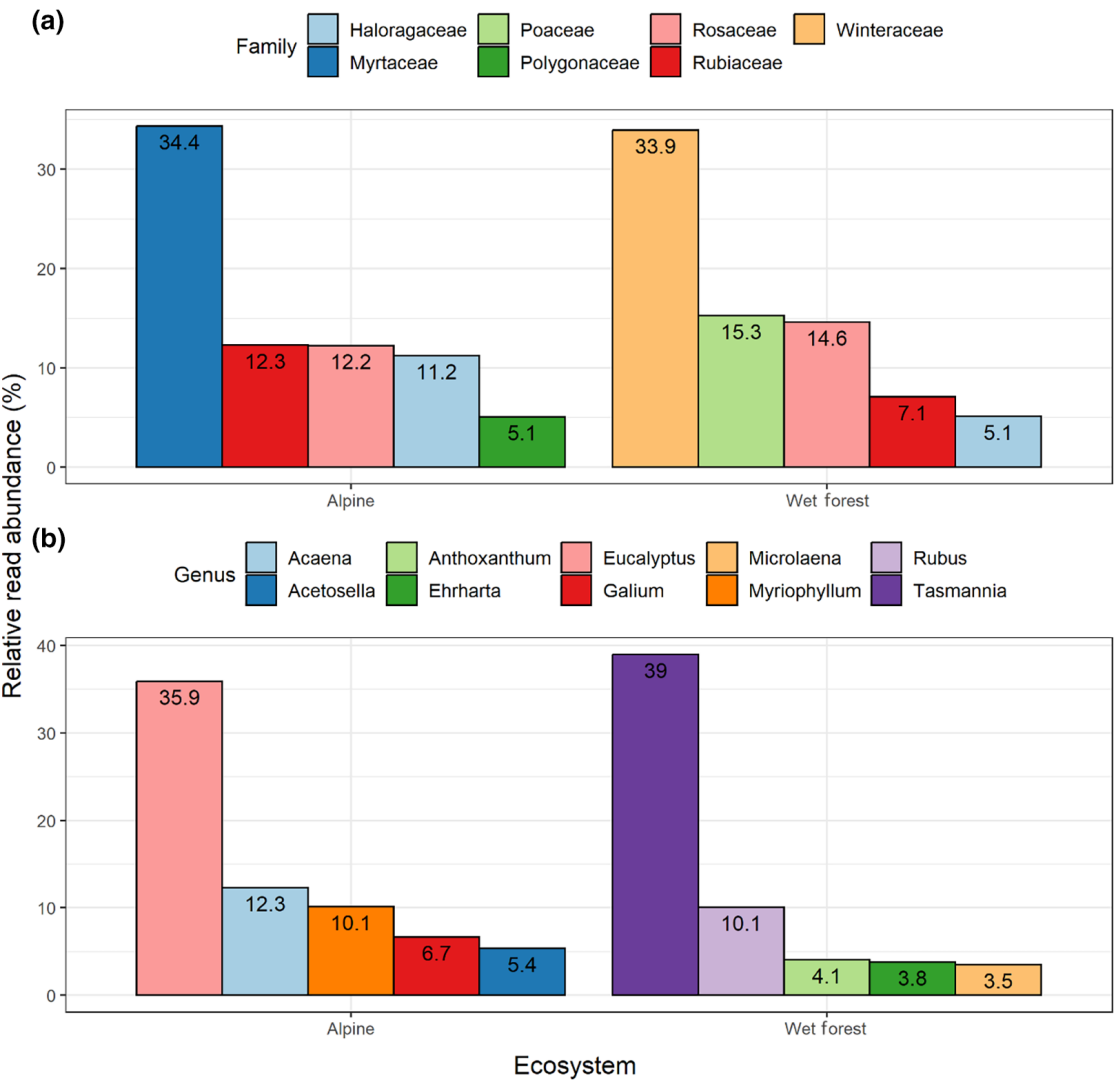
The relative read abundance for the top five dietary items at the taxonomic level of (a) family and (b) genus across all samples collected from alpine and wet forest ecosystems.

In total, 110 genera from 56 families were represented in the alpine samples, the most common OTUs across the samples being from the Myrtaceae (12.8%), Asteraceae (12.5%), Rubiaceae (9.3%), Rosaceae (7.7%), Poaceae (5.1%) and Fabaceae (4.8%). Sequences from the Myrtaceae contributed to the highest proportion of reads (34.3%), followed by Rubiaceae (12.3%) and Rosaceae (12.2%; Figure 1). A greater diversity of genera and families were observed in wet forest samples, with a total of 168 genera from 78 families detected. The most common OTUs from the wet forest study area were Rubiaceae (18.6%), Poaceae (10.3%), Fabaceae (7.1%), Asteraceae (6.4%), Rosaceae (4.8%) and Myrtaceae (3.9%). Sequences from the Winteraceae contributed to the highest proportion of reads (33.9%), followed by Poaceae (15.3%) and Rosaceae (14.6%; Figure 1).

In the alpine samples, the most frequently occurring OTUs across each of the samples (detected in >75% of pellet samples for any of the three gene regions studied) were the native *Acaena* sp., *Asperula* sp., *Celmisia costiniana*, *Eucalyptus* sp., *Galium* sp., *Poa* sp. and *Tasmannia* sp., and the introduced *Acetosella vulgaris* and *Sonchus* sp. The most frequently occurring species across the wet forest samples were the native *Acaena* sp., *Eucalyptus* sp., *Geranium* sp., *Microlaena stipoides*, *Poa* sp., *Rubus* sp. and *Tasmannia* sp., and introduced *Anthoxanthum odoratum* and *Ehrharta erecta*.

There was no difference in the proportion of native and introduced species detected when comparing alpine and wet forest ecosystems (*z* = −0.762, *p* = .447), with diets in both sites consisting of a high proportion of ‘likely native’ species (alpine, 77%; wet forest, 75%). However, the proportion of ‘likely native’ species detected in the diet was significantly less than the proportion of native species that were present in the environment (based on records retrieved from the VicFlora database) in both the alpine (*z* = −4.038, *p* < .001) and wet forest (*z* = −6.117, *p* < .001) study sites, suggesting some preference for exotic species. These results were further supported by the analysis using a subset of the data containing only species‐level OTUs, for both alpine (*z* = −3.0448, *p* < .01) and wet forest (*z* = −11.891, *p* < .001) habitats.

**FIGURE 2:**
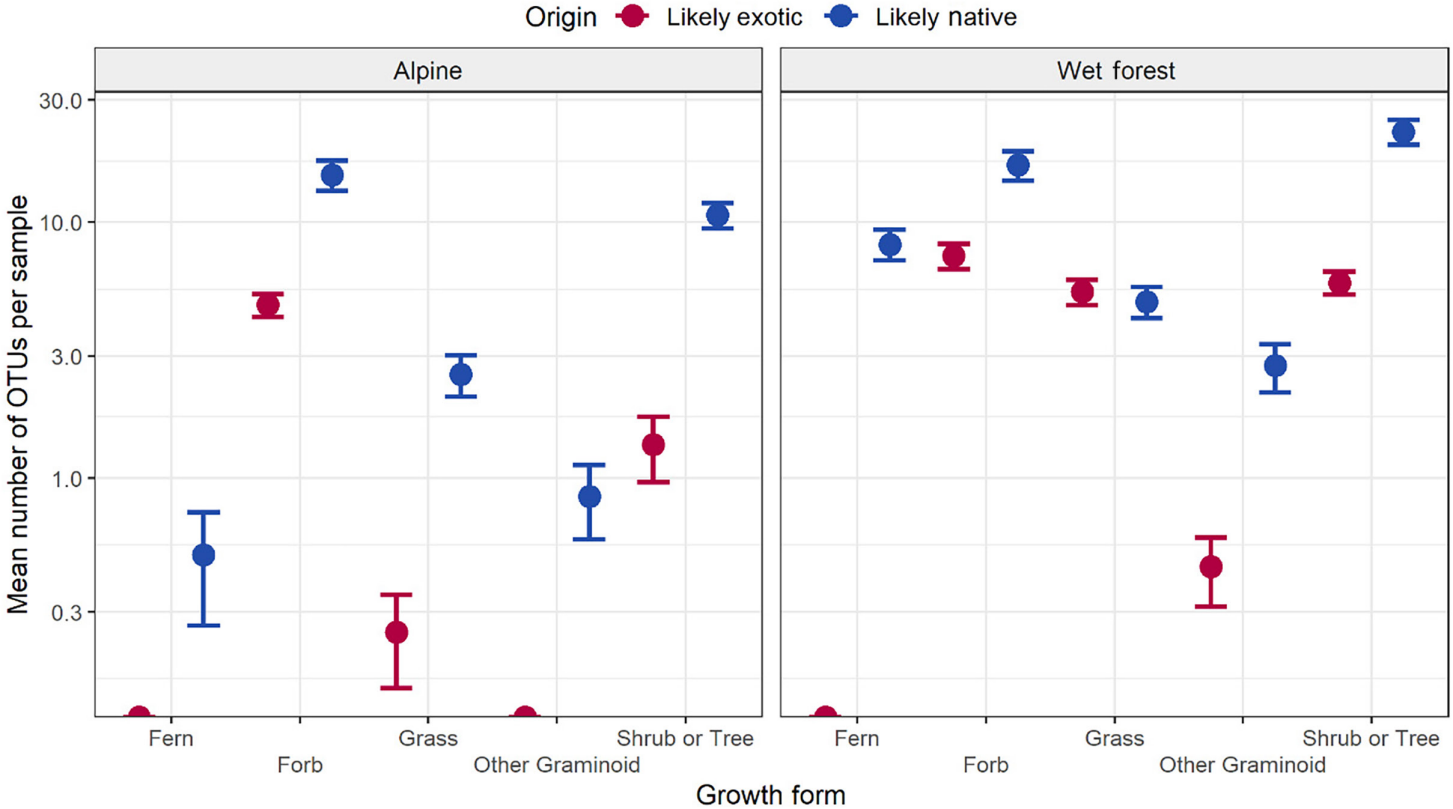
Mean (±1 SE) number of detected operational taxonomic units (OTUs) classified by growth form from sambar deer faecal pellets collected in alpine and wet forest ecosystems. Colours distinguish OTUs based on their origin of ‘likely exotic’ and ‘likely native’.

There was a significant difference in mean number of detected OTUs from different plant growth forms in alpine areas (*χ* ^2^(4) = 81.51, df = 4, *p* < .001; Figure 2), and all pairwise comparisons were significantly different, except for forb and shrub or tree (*z* = −1.53, *p* = .125), other graminoid and grass (*z* = −2.44, *p* = .147) and fern and other graminoid (*z* = 0.499, *p* = .618). A similar result was observed in wet forest samples (*χ* ^2^(4) = 66.77, df = 4, *p* < .001), with pairwise comparisons resulting in a significant difference in mean number of OTUs from different plant growth forms for all comparisons, except for forb and shrub or tree (*z* = 0.445, *p* = .657), forb and grass (*z* = −2.33, *p* = .2), fern and grass (*z* = 2.04, *p* = .416), fern and other graminoids (*z* = −2.00, *p* = .45) and grass and shrub or tree (*z* = 2.77, *p* = .06). Similar results were observed for the analysis incorporating only species‐level OTUs, for both alpine (*χ* ^2^(4) = 67.295, df = 4, *p* < .001) and wet forest (*χ* ^2^(4) = 43.474, df = 4, *p* < .001) habitats, however, comparisons between fern and grass were not significant in alpine samples using the reduced dataset (*z* = 0.06, *p* = .95), or were comparisons between fern and forb (*z* = −2.465, *p* = .137) or fern and shrub (*z* = −2.517, *p* = .118) in the reduced wet forest dataset.

**FIGURE 3:**
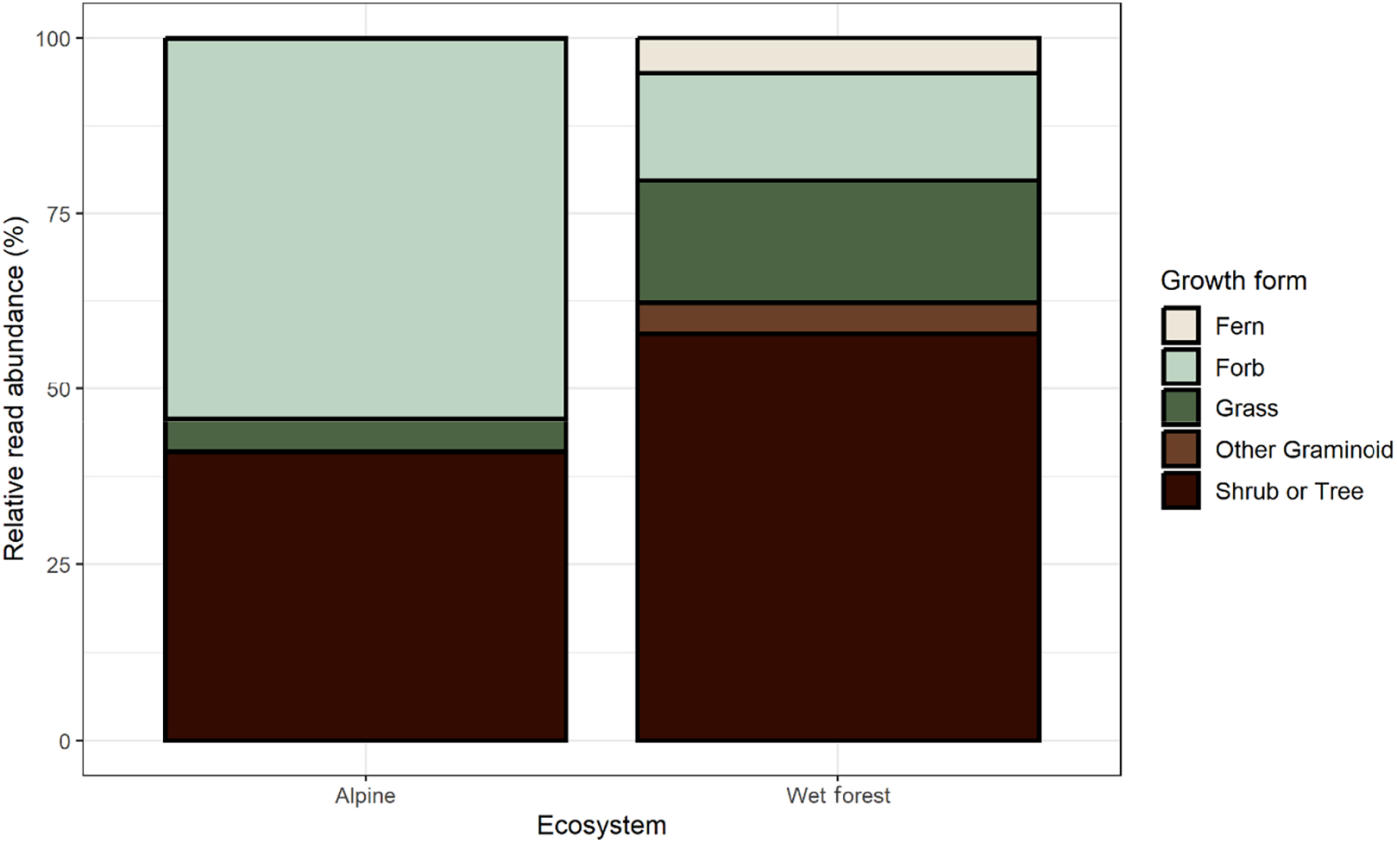
The relative read abundance of each growth form detected across all samples from alpine and wet forest ecosystems.

A comparison of the relative read abundance of each growth form detected among the two ecosystems highlighted a larger proportion of forb items in the diet of sambar deer in alpine environments compared to wet forests (Figure 3). However, based on sequence read depth, the shrub or tree growth form dominated the sambar deer diet in wet forests.

### 3.2 Germination

A total of 2087 seedlings emerged during the germination trials, consisting of 1849 from alpine samples (89%) and 238 from wet forest samples (11%). This was comprised of 68 plant species, 39 of which were native (58%), 26 exotic (38%) and 3 were of uncertain origin (3%; Table 1). Alpine samples contained 32 plant species (20 native, 12 exotic) and wet forest samples contained 50 plant species (24 native, 23 exotic and 3 uncertain). Fourteen species were recorded in both ecosystems (5 native and 9 exotic).

**TABLE 1:** List of plant species that germinated from sambar deer faecal pellets collected from the two study areas.

Family	Species	Common name	Growth form	Origin	Alpine		Wet forest	
					Total	Frequency (%)	Total	Frequency (%)
Apiaceae	*Oreomyrrhis eriopoda*	Australian Caraway	Forb	Native	1	1	0	0
Araliaceae	*Hydrocotyle laxiflora*	Stinking Pennywort	Forb	Native	0	0	2	3
Asteraceae	*Argyrotegium fordianum*		Forb	Native	1	1	0	0
Asteraceae	*Cassinia aculeata*	Common Cassinia	Shrub	Native	0	0	3	3
Asteraceae	*Cassinia* sp.		Shrub	Native	3	4	0	0
Asteraceae	*Coronidium monticola*		Forb	Native	2	1	0	0
Asteraceae	*Cotula alpina*	Alpine Cotula	Forb	Native	83	22	0	0
Asteraceae	*Erigeron bonariensis*	Flaxleaf Fleabane	Forb	Exotic	1	1	1	1
Asteraceae	*Euchiton involucratus*	Common Cudweed	Forb	Native	0	0	1	1
Asteraceae	*Gamochaeta purpurea*	Spike Cudweed	Forb	Exotic	0	0	4	5
Asteraceae	*Hypochaeris radicata*	Cats‐ear	Forb	Exotic	3	4	1	1
Asteraceae	*Lagenophora stipitata*	Blue Bottle‐daisy	Forb	Native	2	1	0	0
Asteraceae	*Leptinella filicula*	Mountain Cotula	Forb	Native	0	0	1	1
Asteraceae	*Sonchus oleraceus*	Sow Thistle	Forb	Exotic	8	9	1	1
Asteraceae	*Symphyotrichum subulatum*	Aster‐weed	Forb	Exotic	2	1	0	0
Asteraceae	*Taraxacum sect. taraxacum*	Dandelion	Forb	Exotic	0	0	5	6
Brassicaceae	*Cardamine lilacina*		Forb	Native	1	1	0	0
Campanulaceae	*Lobelia anceps*	Angled Lobelia	Forb	Native	0	0	1	1
Campanulaceae	*Wahlenbergia gracilis*	Sprawling Bluebell	Forb	Native	0	0	4	3
Caryophyllaceae	*Cerastium glomeratum*	Sticky Mouse‐ear Chickweed	Forb	Exotic	129	36	13	9
Caryophyllaceae	*Scleranthus biflorus*	Twin‐flower Knawel	Forb	Native	179	47	0	0
Cyperaceae	*Carex appressa*	Tall Sedge	Other Graminoid	Native	0	0	2	3
Cyperaceae	*Carex breviculmis*	Short‐stem Sedge	Other Graminoid	Native	7	4	0	0
Cyperaceae	*Carex gaudichaudiana*	Fen Sedge	Other Graminoid	Native	0	0	3	1
Cyperaceae	*Carex inversa*	Knob Sedge	Other Graminoid	Native	0	0	5	5
Fabaceae	*Lotus corniculatus*	Bird's‐foot Trefoil	Forb	Exotic	0	0	2	3
Fabaceae	*Trifolium glomeratum*	Clustered Clover	Forb	Exotic	11	5	1	1
Fabaceae	*Trifolium repens var. repens*	White Clover	Forb	Exotic	32	7	4	5
Gentianaceae	*Centaurium erythraea*	Common Centaury	Forb	Exotic	0	0	6	6
Haloragaceae	*Gonocarpus tetragynus*	Common Raspwort	Forb	Native	0	0	1	1
Iridaceae	*Romulea rosea var. australis*	Onion‐grass	Forb	Exotic	0	0	1	1
Juncaceae	*Juncus bufonius*	Toad Rush	Other Graminoid	Uncertain	0	0	10	11
Juncaceae	*Juncus holoschoenus*		Other Graminoid	Native	0	0	28	22
Juncaceae	*Juncus planifolius*	Broad‐leaf Rush	Other Graminoid	Native	0	0	6	6
Juncaceae	*Luzula modesta*		Other Graminoid	Native	19	16	1	1
Lamiaceae	*Mentha laxiflora*	Forest Mint	Forb	Native	0	0	3	3
Lamiaceae	*Prunella vulgaris*	Self‐heal	Forb	Exotic	0	0	1	1
Myrtaceae	*Eucalyptus* sp.		Tree	Native	0	0	1	1
Myrtaceae	*Leptospermum* sp.		Shrub	Native	4	5	2	3
Onagraceae	*Epilobium billardiereanum*		Forb	Native	3	4	15	5
Onagraceae	*Epilobium ciliatum*	Glandular Willow‐herb	Forb	Exotic	17	2	0	0
Plantaginaceae	*Callitriche muelleri*	Round Water‐starwort	Forb	Native	0	0	1	1
Plantaginaceae	*Gratiola peruviana*	Austral Brooklime	Forb	Native	0	0	6	8
Plantaginaceae	*Plantago euryphylla*	Broad Plantain	Forb	Native	2	1	0	0
Plantaginaceae	*Plantago major*	Greater Plantain	Forb	Exotic	0	0	2	3
Plantaginaceae	*Veronica arvensis*	Wall Speedwell	Forb	Exotic	5	2	3	3
Poaceae	*Agrostis capillaris*	Brown‐top Bent	Grass	Exotic	193	14	7	6
Poaceae	*Axonopus fissifolius*	Narrow‐leafed Carpet Grass	Grass	Exotic	0	0	1	1
Poaceae	*Ehrharta erecta*	Panic Veldt‐grass	Grass	Exotic	0	0	3	4
Poaceae	*Eragrostis brownii*	Common Love‐grass	Grass	Native	0	0	3	4
Poaceae	*Holcus lanatus*	Yorkshire Fog	Grass	Exotic	0	0	1	1
Poaceae	*Phleum pratense*	Timothy Grass	Grass	Exotic	4	1	0	0
Poaceae	*Poa hothamensis*	Ledge‐grass	Grass	Native	7	7	0	0
Poaceae	*Unidentified Poaceae* sp. 1		Grass	Uncertain	0	0	1	1
Poaceae	*Unidentified Poaceae* sp. 2		Grass	Uncertain	0	0	1	1
Poaceae	*Vulpia bromoides*	Squirrel‐tail Fescue	Grass	Exotic	0	0	3	3
Polygonaceae	*Acetosella vulgaris*	Sheep Sorrel	Forb	Exotic	1066	65	46	5
Polygonaceae	*Persicaria decipiens*	Slender Knotweed	Forb	Native	0	0	1	1
Ranunculaceae	*Ranunculus victoriensis*	Victorian Buttercup	Forb	Native	2	2	0	0
Rosaceae	*Aphanes arvensis*	Parsley Piert	Forb	Exotic	0	0	3	4
Rosaceae	*Rubus parvifolius*	Small‐leaf Bramble	Shrub	Native	1	1	0	0
Rosaceae	*Rubus ulmifolius*	Blackberry	Shrub	Exotic	0	0	4	1
Rubiaceae	*Asperula conferta*	Common Woodruff	Forb	Native	55	32	10	9
Rubiaceae	*Galium leiocarpum*		Forb	Native	0	0	7	4
Rutaceae	*Asterolasia trymalioides*	Alpine Star‐bush	Shrub	Native	1	1	0	0
Solanaceae	*Solanum nigrum*	Black Nightshade	Forb	Exotic	0	0	1	1
Urticaceae	*Australina pusilla* subsp. *muelleri*	Shade Nettle	Forb	Native	1	1	0	0
Urticaceae	*Urtica incisa*	Scrub Nettle	Forb	Native	4	2	5	3

*Note*: For alpine samples (*n* = 81) and wet forest samples (*n* = 79), the plant family, common name, growth form, origin, total germinants and percentage of pellet groups that each species emerged from (frequency) are shown.

The mean (±1 SE) species richness per punnet in the alpine samples (3.05 ± 0.26) was significantly higher than observed in the wet forest samples (1.78 ± 0.19, *W* = 4334, *p* < .001). Germination from non‐stratified and cold–wet stratified alpine faecal samples were highly correlated for number of germinants per punnet (*r* = .85, *p* < .001), number of different species per punnet (*r* = .73, *p* < .001) and number of germinants for each different species (*r* = .72, *p* < .001), resulting in these two alpine datasets being pooled for further analysis.

**FIGURE 4:**
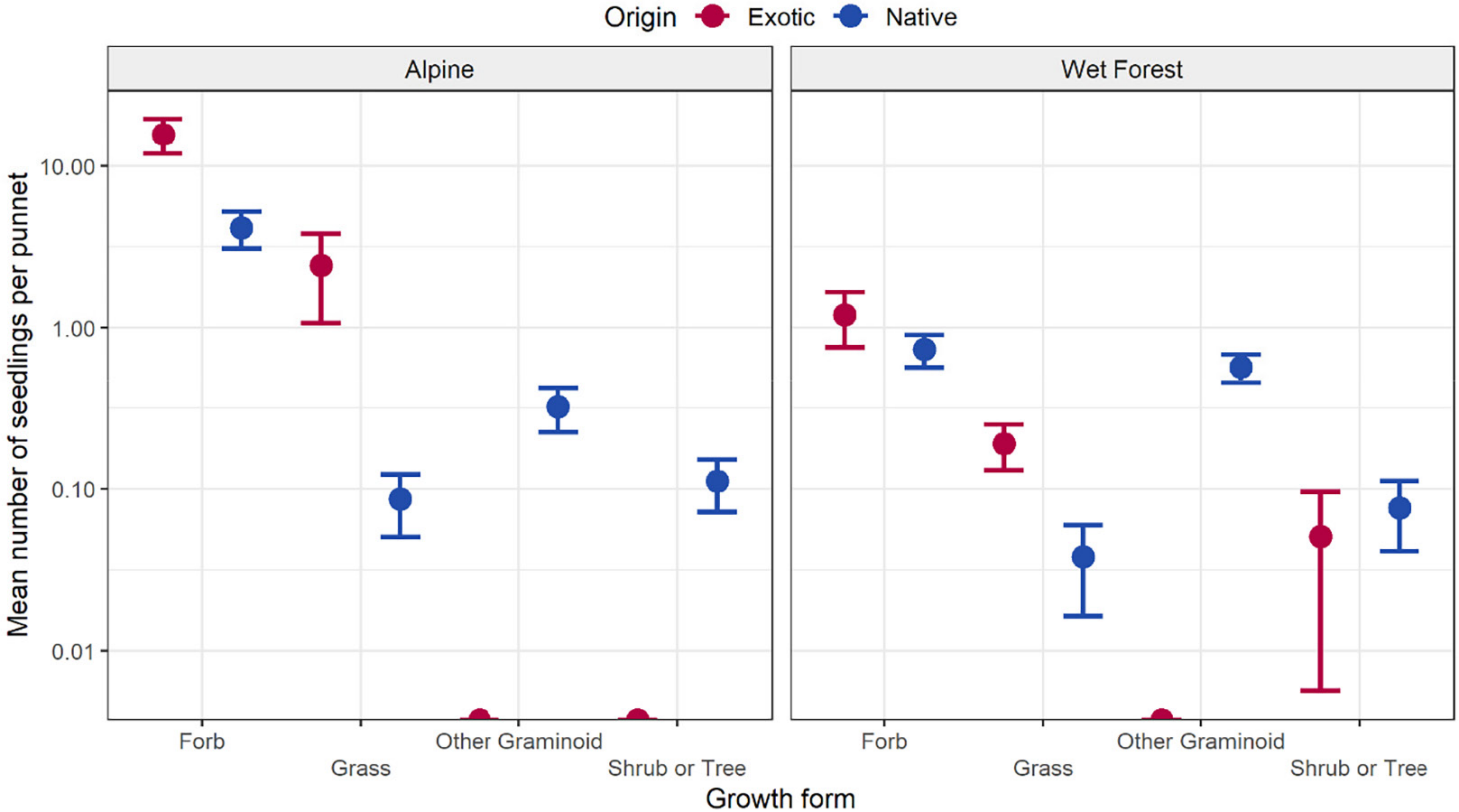
Mean (±1 SE) number of seedlings per punnet for each growth form that emerged from sambar deer faecal pellets collected in alpine and wet forest ecosystems. Colours distinguish seedlings based on their origin.

A total of 1849 seedlings emerged in the alpine samples, comprising 378 native (20% of total seedlings) and 1471 exotic seedlings (80%), with a significantly higher mean number of exotic seedlings per punnet (18.2 ± 4.91) than native species (4.67 ± 1.15; *W* = 4033, *p* = .011; Figure 4). In the wet forest samples, a total of 238 seedlings emerged comprising 112 native (47% of total seedlings), 114 exotic (48%) and 12 seedlings of uncertain origin (5%). However, no significant difference between the mean number of exotic seedlings (1.44 ± 0.498) and natives per pellet group (1.42 ± 0.196, *W* = 234, *p* = .076) was observed in wet forest samples.

The most frequently germinating species in the alpine samples (from >10% of pellet samples) were the exotic forbs *Acetosella vulgaris* and *Cerastium glomeratum*, and the exotic grass *Agrostis capillaris*, which together comprised approximately 75% of all alpine seedlings. The most frequently germinating native species were the forbs *Cotula alpina, Scleranthus biflorus* and *Asperula conferta*, and the native rush *Luzula modesta*. Only two species were observed in >10% of wet forest samples, the rushes *Juncus holoschoenus* and *Juncus bufonius*.

Forb seedlings were higher in mean abundance per punnet in alpine samples than all other growth forms (*χ* ^2^(3) = 154.65, df = 3, *p* < .001; Figure 4), which did not differ from one another. Forbs were also the most abundant in the wet forest samples (*χ* ^2^(3) = 50.933, df = 3, *p* < .001), with pairwise comparisons resulting in a significant difference among forb and grass (*z* = −4.98, *p* < .001), forb and shrub or tree (*z* = −6.38, *p* < .001), grass and other graminoids (*z* = 3.19, *p* = .008) and other graminoids and shrub or tree (*z* = −4.59, *p* < .001).

The mean (±1 SE) number of seeds dispersed daily by an individual sambar deer within the alpine study area was estimated as 816 (±193), which included 652 (±176) exotic seeds and 166 (±41) native seeds. Estimated seed load dispersed daily by sambar deer in the wet forest study area was lower, comprising 50 (±7) native and 50 (±16) exotic seeds, with an estimated mean daily dispersal of 100 (±19) seeds by individual sambar deer in this ecosystem (Appendix S1).

## 4 DISCUSSION

We simultaneously assessed the dietary foraging characteristics and seed dispersal capability of a non‐native herbivore and showed that the dietary composition across two ecologically distinct landscapes comprised a higher proportion of ‘likely native’ plant species. However, this result contrasted with observations from germination trials, where a higher number of exotic seedlings emerged from faecal pellets in both study areas.

### 4.1 Diet composition

The use of three gene regions for dietary analysis allowed us to explore the number of plant species foraged by sambar deer and unsurprisingly, the plant species richness of the diet was much higher than detected through germination trials alone. Previous dietary studies of herbivores in Australia have been undertaken with microhistological and macroscopic techniques that involve complex sieving and fine‐scale plant fragment identification (Davis et al., 2008; Norbury, 1988). With a smaller number of samples and a technique capable of faster results (Khanam et al., 2016), we detected a total of 168 genera from 78 families, which increased the number of previously known species eaten by sambar deer in wet forest habitats of south‐eastern Australia (Forsyth & Davis, 2011). This technique also allowed us to detect several rare plant species in the diet of sambar deer, including *Ozothamnus stirlingii* and *Wittsteinia vacciniacea*, which are both restricted in distribution to wet forest ecosystems, and are less likely to be detected with other methods due to their rare occurrence in the environment and diet (Garnick et al., 2018).

In both alpine and wet forest ecosystems, the diet of sambar deer was comprised of a higher proportion of species that are ‘likely native’, which is not unexpected given these areas are conserved as National Parks. However, the proportion of ‘likely exotic’ species in the diet was significantly higher than would be expected based on the proportion of native and exotic items in the study areas, suggesting that sambar deer might be targeting exotic species. In Australian alpine and wet forest ecosystems, native plant species have not evolved in the presence of novel consumers such as sambar deer, and are likely vulnerable to herbivory due to a lack of adequate defences (Hokkanen & Pimentel, 1989; Parker & Hay, 2005). Conversely, considering deer have many indigenous species in all continents except Antarctica and Australia, it is plausible that many exotic plant species established in Australia have co‐evolved alongside deer species in their native ranges, and therefore, the presence of sambar deer in alpine and wet forest ecosystems could enable an increase in the relative abundance and species richness of exotic plants that have similar home origins and adaptations to deer herbivory (Parker et al., 2006; Parker & Hay, 2005).

Forbs were well represented in deer diets in alpine ecosystems, while forbs, shrubs and trees were equally well represented in wet forests. Although dietary selection depends upon many factors including nutritional requirements, digestion ability and competition (Kushwaha et al., 2004; Stewart et al., 2003), sambar deer diet heavily depends on forage availability (Leslie, 2011), with previous research suggesting they are browsers (Semiadi et al., 1995), intermediate feeders (Forsyth & Davis, 2011) or grazers (Padmalal et al., 2003), depending on the environment they inhabit. It is unsurprising that in forb‐dominated alpine ecosystems, the diet was heavily comprised of forb species, while in shrub‐dominated wet forest ecosystems, the diet was dominated by browse material. Nevertheless, using different methods, we observed a higher contribution of forbs to the diet relative to the study performed by Forsyth and Davis (2011). It is possible that DNA analysis techniques provide superior resolution of the readily digested and often underestimated forb growth form in microhistological and macroscopic analysis (Kessler et al., 1981), and that sambar deer diet may change throughout the year (Bennett, 2008). If the latter, further research on temporal variation in sambar deer diet would resolve a significant knowledge gap in alpine and wet forest ecosystems.

### 4.2 Deer as vectors for seed dispersal

In both ecosystems, sambar deer dispersed a wide range of both native and exotic plant species through endozoochory. However, a larger number of exotic seedlings emerged from faecal pellets despite our results suggesting exotic species comprise a much smaller component of the overall diet.

In alpine and wet forest ecosystems of south‐eastern Australia, sambar deer coexist with numerous herbivores and omnivores including the native swamp wallaby (*Wallabia bicolor*) and common wombat (*Vombatus ursinus*), and the introduced European rabbit (*Oryctolagus cuniculus*), hare (*Lepus europaeus*), red fox (*Vulpes vulpes*) and feral pig (*Sus scrofa*). The role of each species as seed dispersers has been investigated in a variety of ecosystems worldwide (Auld et al., 2007; Bourgeois et al., 2005; Evans et al., 2006; Green et al., 2013; Matías et al., 2010; O'Connor & Kelly, 2012), and although factors such as size, diet, movement patterns and digestive system differences vary the number of plant species seeds potentially dispersed by each, deer species deposit a larger number of seeds (Claridge et al., 2016; Davis et al., 2010; Eycott et al., 2007). In alpine ecosystems, we estimated that sambar deer dispersed 847 (±201) seeds per day and that approximately 80% of these seeds were exotic. This is problematic, as the native, low‐growing forb species observed at high elevation typically rely on gravity and/or wind as main dispersal mechanisms and may have limited capacity for long‐distance seed dispersal via the process of endozoochory (Morgan & Venn, 2017). Consequently, browsing by sambar deer, now a dominant herbivore in alpine ecosystems, may prevent these typical methods of seedling establishment, and subsequently limit the recruitment of native plant species (Côté et al., 2004).

Deer species can act as dispersal mechanism for exotic plant seeds, and may play a substantial role in exotic species invasion (Vellend, 2002; Williams et al., 2008). Faecal samples collected from wet forest ecosystems contained viable seeds of the exotic variety of blackberry (*Rubus ulmifolius*), one of the most significant environmental and agricultural weeds in south‐eastern Australia (Deehan et al., 2007). Although this invasive weed typically has low germination rates (Evans et al., 1998), establishment of a single seed may be enough for the re‐invasion of whole landscapes (Delaisse et al., 2023). In Australia, the main consumers of the fruit are native emus (*Dromaius novaehollandiae*) and introduced red foxes, which both act as dispersal agents of blackberry seeds (Spennemann, 2021). The blackberry genus was frequently detected in the diet of sambar deer in this study, and therefore, it is possible that with increased seed supply through foraging, sambar deer may increase the likelihood of seedling recruitment of blackberry in the landscapes they inhabit.

*Acetosella vulgaris* seedlings in faecal pellets were clearly the most abundant. A pasture weed in Australia and overseas (Korpelainen, 1993), *A. vulgaris* is one of the more common invasive species in the Australian Alps (McDougall et al., 2005), and has high seed production (Pickering et al., 2003). However, the role played by sambar deer as effective dispersers of these species depends heavily on their movement within the landscape (Davis et al., 2010; Gill & Beardall, 2001). Previous research on sambar deer movement using radio‐telemetric techniques has observed mean annual home ranges of between 10 and 13.3 km^2^ for stags and 3 and 6.46 km^2^ for hinds (Chundawat et al., 2007; Sankar, 1994). Home range movements of sambar deer would constitute long‐distance dispersal of plants (Cain et al., 2000), and for some plant species, this method may be advantageous over other dispersal mechanisms (Davis et al., 2010; Tackenberg et al., 2003). Considering the predicted range expansions of deer species in south‐eastern Australia, further research into the geographical barriers for movement, and the ecological limits of sambar deer in both study regions would further detail potential pathways for invasion by exotic species through endozoochory (Davis et al., 2016; Webley et al., 2007).

### 4.3 Management implications

Mitigating the impacts of non‐native herbivores in native ecosystems is imperative for protecting plant species and vegetation communities (Augustine & Jordan, 1998). Our results demonstrate that sambar deer are heavily foraging native species yet dispersing weeds, which is clearly detrimental to native ecosystems.

The sheer number of plant species that were observed in the diet and germinated from faecal pellets collected at a single point in time emphasises that endozoochory by sambar has the capacity to alter the composition of ecosystems, and in the alpine and wet forest ecosystems of south‐eastern Australia, sambar deer may be one of the most important vectors for long‐distance seed dispersal. Clearly, a major concern is the ability of deer to access remote areas, which could successfully move seeds between isolated patches of similar habitats (Poschlod & Bonn, 1998). Further research of sambar deer movement patterns and home ranges in south‐eastern Australia should be undertaken, as this will determine the extent to which the species can act as long‐distance dispersal mechanisms of native and exotic plant seed and may additionally lead to more focussed management actions.

Density and grazing intensity are two critical factors in determining the degree to which species affect broader ecosystems and should be considered when managing areas inhabited by non‐native herbivores (Anderson, 1994). Sambar deer populations continue to increase in south‐eastern Australia (Watter et al., 2020), and previous research has highlighted the high levels of plant biomass consumed by sambar deer, eclipsing the consumption by other coexisting native herbivores (Bennett, 2008). In areas where deer density is high, and/or rare plant species and communities are established, sambar deer should be recognised as a serious threat, and either the removal of individuals or implementation of exclusion‐based methods should be explored.

## AUTHOR CONTRIBUTIONS

**Matthew J. Quin:** Conceptualization (equal); formal analysis (equal); methodology (equal); writing – original draft (equal); writing – review and editing (equal). **John W. Morgan:** Conceptualization (equal); methodology (equal); writing – review and editing (equal). **Nicholas P. Murphy:** Conceptualization (equal); methodology (equal); supervision (lead); writing – original draft (equal); writing – review and editing (equal).

## CONFLICT OF INTEREST STATEMENT

The authors declare no conflicts of interest.

## STATEMENT OF INCLUSION

Our research was discussed with local management authorities and community groups to develop appropriate questions and methods. We considered previous literature published within the study area and whenever relevant, these studies were cited. We recognise that more could have been done to engage with international scientists with deer ecology expertise, and this will be considered in future research.

## Supporting information


Appendix S1.

## Data Availability

Data are available from the Dryad Digital Repository https://doi.org/10.5061/dryad.d51c5b08j.
