# Supplementary material for: Assessing the diet and seed dispersal ability of non‐native sambar deer (Rusa unicolor) in native ecosystems of south‐eastern Australia
Source: Ecol Evol. 2023 Nov 27;13(11):e10711. doi: 10.1002/ece3.10711 (PMC10682569; doi:10.1002/ece3.10711)
Supplement: Supplementary file 1 — Appendix S1. [file ECE3-13-e10711-s001.docx]

Supplementary Material: Assessing the diet and seed dispersal ability of non-native sambar deer (*Rusa unicolor*) in native ecosystems of south-eastern Australia.

**Table S1.** Primer names, sequences and expected size for *rbcL*, ITS2 and *trnL* gene regions.

**Table S2.** PCR programs used for each gene region during first rounds of amplification.

**Method for the PCR steps**

Each first round PCR mixture included 15.5 μL of H_2_O, 5 μL of MyTaq™ Red Reaction Buffer (Bioline), 0.5 μL of each forward and reverse primer (10 μM), 0.5 μL of MyTaq™ DNA polymerase (Bioline), and 3 μL of DNA template (ranging in concentration from 4.3–158.5 ng µL^-1^). Where samples failed to amplify, DNA samples were diluted at 1/10 and 1/100 until amplification was successful.

Illumina adaptors and dual indexes were attached during a second PCR containing 9.45 μL of H_2_O, 3 μL of MyTaq™ Red Reaction Buffer (Bioline), 0.75 μL of the index primer pair, 0.3 μL of MyTaq™ DNA polymerase (Bioline), and 1.5 μL of DNA PCR template). PCR conditions included an initial denaturation of 95 °C for 3 min, followed by 10 cycles of 95 °C for 20 s, 60 °C for 15 s, and 72 °C for 30 s.

Final PCR products were cleaned using SPRI magnetic beads to remove non-specific DNA fragments. PCR products were quantified using a Thermo Fisher NanoDrop Lite Spectrophotometer, and samples were normalised using H_2_O.

**Ssequence preparation and data filtering**

For ITS2 and *trnL* sequences, forward and reverse sequences for each sample were merged using the fastq_mergepairs function in the sequence analysis tool USEARCH v11.0.667 (Edgar 2010). The fastx_truncate function was used to remove the primers, and sequences were then filtered based on length using the fastq_filter function. Contaminants and singleton sequences were removed using the fastx_uniques function, and the -unoise3 function was used to generate OTUs. All *rbcL* sequences were analysed separately as forward and reverse sequences, as the length of these sequences prevented merging. However, sequences that were identified to the same species were merged in later analyses. Any OTUs present in negative extractions or H_2_O controls were removed from the entire dataset, as were any OTUs with a read depth <5. After the completion of filtering, OTUs from each of the three extraction sub-samples were merged into a single sample.

The taxonomic assignment of OTUs was determined by comparison with the National Centre for Biotechnology Information (NCBI) GenBank nucleotide database. BLAST (Basic Local Alignment Search Tool; Altschul *et al*. 1990) was used to search each OTU against NCBI GenBank with default parameters.

An OTU was assigned to species level when a ≥97% match of a reference sequence to the full length of the consensus OTU sequence was observed. Matches that were not able to be made to species level were assigned to the lowest taxonomic level possible. The Atlas of Living Australia (www.ala.org.au) and Royal Botanic Gardens Victoria VicFlora (www.vicflora.rbg.vic.gov.au) were used to ensure that any identification was plausible, by ensuring that species, genus or family identified were within or surrounding the study areas. The Royal Botanic Gardens Victoria VicFlora was also used to develop a comprehensive list of all identified plant species within each study area (<https://vicflora.rbg.vic.gov.au/flora/checklist>).

**Table S3**. Taxonomic table displaying the assignment of OTUs in the Alpine samples. Sum refers to total number of sequence reads for each OTU. Count refers to the number of samples an OTU was detected in, and average read depth highlights the total reads on average for OTUs across detected samples.

| OTU ID | Locus | Class | Order | Family | Genus | Species | Growth Form | Origin | Sum | Count | Average Read Depth |
| --- | --- | --- | --- | --- | --- | --- | --- | --- | --- | --- | --- |
| Otu1 | trnL | Magnoliopsida | Myrtales | Myrtaceae | Eucalyptus |  | Shrub or Tree | Likely Native | 139875 | 18 | 7771 |
| Otu2 | trnL | Magnoliopsida | Rosales | Rosaceae | Acaena |  | Forb | Likely Native | 94094 | 19 | 4952 |
| Otu4 | trnL | Magnoliopsida | Caryophyllales | Polygonaceae | Acetosella | vulgaris | Forb | Likely Exotic | 72271 | 20 | 3614 |
| Otu5 | trnL | Magnoliopsida | Gentianales | Rubiaceae | Galium |  | Forb | Likely Native | 59486 | 19 | 3131 |
| Otu6 | trnL | Magnoliopsida | Gentianales | Rubiaceae | Asperula |  | Forb | Likely Native | 52957 | 19 | 2787 |
| Otu7 | trnL | Magnoliopsida | Gentianales | Rubiaceae |  |  | Forb | Unknown | 27934 | 6 | 4656 |
| Otu8 | trnL | Pinopsida | Pinales | Podocarpaceae | Podocarpus | lawrencei | Shrub or Tree | Likely Native | 15838 | 8 | 1980 |
| Otu11 | trnL | Magnoliopsida | Poales | Poaceae | Poa |  | Grass | Likely Native | 14016 | 18 | 779 |
| Otu13 | trnL | Magnoliopsida | Asterales | Asteraceae |  |  | Forb | Unknown | 10253 | 3 | 3418 |
| Otu14 | trnL | Magnoliopsida | Proteales | Proteaceae | Grevillea |  | Shrub or Tree | Likely Native | 7903 | 11 | 718 |
| Otu15 | trnL | Magnoliopsida | Sapindales | Rutaceae |  |  | Shrub or Tree | Unknown | 7234 | 17 | 426 |
| Otu16 | trnL | Magnoliopsida | Asterales | Asteraceae | Sonchus |  | Forb | Likely Exotic | 9513 | 17 | 560 |
| Otu17 | trnL | Magnoliopsida | Fabales | Fabaceae | Pultenaea |  | Shrub or Tree | Likely Native | 6046 | 10 | 605 |
| Otu18 | trnL | Magnoliopsida | Rosales | Rosaceae |  |  | Shrub or Tree | Unknown | 4777 | 5 | 955 |
| Otu20 | trnL | Magnoliopsida | Malpighiales | Salicaceae | Salix | cinerea | Shrub or Tree | Likely Exotic | 2650 | 7 | 379 |
| Otu22 | trnL | Magnoliopsida | Malpighiales | Violaceae | Melicytus | dentatus | Shrub or Tree | Likely Native | 2266 | 12 | 189 |
| Otu24 | trnL | Magnoliopsida | Myrtales | Onagraceae | Epilobium |  | Forb | Likely Native | 2151 | 12 | 179 |
| Otu25 | trnL | Magnoliopsida | Canellales | Winteraceae | Tasmannia |  | Shrub or Tree | Likely Native | 1874 | 16 | 117 |
| Otu30 | trnL | Magnoliopsida | Rosales | Urticaceae |  |  | Forb | Unknown | 1444 | 3 | 481 |
| Otu31 | trnL | Magnoliopsida | Lamiales | Plantaginaceae | Veronica |  | Forb | Likely Native | 1149 | 3 | 383 |
| Otu32 | trnL | Magnoliopsida | Gentianales | Rubiaceae |  |  | Forb | Unknown | 1101 | 10 | 110 |
| Otu35 | trnL | Magnoliopsida | Malvales | Thymelaeaeae | Pimelea |  | Shrub or Tree | Likely Native | 770 | 5 | 154 |
| Otu36 | trnL | Magnoliopsida | Lamiales | Lamiaceae | Prostanthera |  | Shrub or Tree | Likely Native | 1157 | 1 | 1157 |
| Otu40 | trnL | Magnoliopsida | Ranunculales | Ranunculaceae | Ranunculus | repens | Forb | Likely Exotic | 496 | 11 | 45 |
| Otu47 | trnL | Magnoliopsida | Lamiales | Plantaginaceae | Plantago | lanceolata | Forb | Likely Exotic | 386 | 2 | 193 |
| Otu48 | trnL | Magnoliopsida | Geraniales | Geraniaceae | Geranium |  | Forb | Likely Native | 461 | 8 | 58 |
| Otu50 | trnL | Polypodiopsida | Polypodiales | Dryopteridaceae | Polystichum | proliferum | Fern | Likely Native | 339 | 5 | 68 |
| Otu52 | trnL | Magnoliopsida | Poales | Juncaceae | Luzula | modesta | Grass-like Plants | Likely Native | 491 | 6 | 82 |
| Otu54 | trnL | Magnoliopsida | Poales | Poaceae |  |  | Grass | Unknown | 363 | 5 | 73 |
| Otu71 | trnL | Magnoliopsida | Myrtales | Myrtaceae | Kunzea |  | Shrub or Tree | Likely Native | 251 | 3 | 84 |
| Otu72 | trnL | Magnoliopsida | Fabales | Fabaceae | Acacia |  | Shrub or Tree | Likely Native | 380 | 4 | 95 |
| Otu74 | trnL | Magnoliopsida | Poales | Poaceae | Anthoxanthum | odoratum | Grass | Likely Exotic | 189 | 3 | 63 |
| Otu81 | trnL | Magnoliopsida | Lamiales | Orobanchaceae | Euphrasia |  | Forb | Likely Native | 153 | 1 | 153 |
| Otu84 | trnL | Pinopsida | Pinales | Cupressaceae |  |  | Shrub or Tree | Unknown | 150 | 1 | 150 |
| Otu85 | trnL | Pinopsida | Pinales | Cupressaceae |  |  | Shrub or Tree | Unknown | 178 | 3 | 59 |
| Otu86 | trnL | Magnoliopsida | Apiales | Araliaceae | Hydrocotyle | sibthorpioides | Forb | Likely Native | 139 | 2 | 70 |
| Otu88 | trnL | Magnoliopsida | Fabales | Fabaceae | Templetonia | egena | Shrub or Tree | Likely Exotic | 114 | 4 | 29 |
| Otu95 | trnL | Magnoliopsida | Solanales | Solanaceae | Solanum |  | Shrub or Tree | Likely Exotic | 109 | 1 | 109 |
| Otu99 | trnL | Magnoliopsida | Myrtales | Myrtaceae | Eucalyptus |  | Shrub or Tree | Likely Native | 35 | 2 | 18 |
| Otu107 | trnL | Magnoliopsida | Fabales | Fabaceae | Bossiaea |  | Shrub or Tree | Likely Native | 45 | 1 | 45 |
| Otu109 | trnL | Magnoliopsida | Rosales | Rosaceae | Acaena |  | Forb | Likely Native | 209 | 7 | 30 |
| Otu113 | trnL | Magnoliopsida | Gentianales | Rubiaceae |  |  | Forb | Unknown | 12 | 1 | 12 |
| Otu114 | trnL | Magnoliopsida | Asterales | Goodeniaceae | Goodenia |  | Shrub or Tree | Likely Native | 67 | 2 | 34 |
| Otu116 | trnL | Magnoliopsida | Poales | Cyperaceae | Carex | breviculmis | Grass-like Plants | Likely Native | 92 | 2 | 46 |
| Otu117 | trnL | Magnoliopsida | Myrtales | Myrtaceae |  |  | Shrub or Tree | Unknown | 52 | 2 | 26 |
| Otu118 | trnL | Magnoliopsida | Oxalidales | Oxalidaceae | Oxalis | corniculata | Forb | Likely Exotic | 69 | 1 | 69 |
| Otu119 | trnL | Polypodiopsida | Polypodiales | Blechnaceae | Blechnum | fluviatile | Fern | Likely Native | 66 | 1 | 66 |
| Otu121 | trnL | Magnoliopsida | Gentianales | Rubiaceae | Galium |  | Forb | Likely Native | 14 | 1 | 14 |
| Otu122 | trnL | Magnoliopsida | Malpighiales | Violaceae | Melicytus | dentatus | Shrub or Tree | Likely Native | 51 | 1 | 51 |
| Otu124 | trnL | Magnoliopsida | Asterales | Campanulaceae | Wahlenbergia | multicaulis | Forb | Likely Native | 65 | 2 | 33 |
| Otu125 | trnL | Magnoliopsida | Fabales | Fabaceae | Daviesia |  | Shrub or Tree | Likely Native | 65 | 1 | 65 |
| Otu132 | trnL | Magnoliopsida | Asterales | Asteraceae | Cirsium | vulgare | Forb | Likely Exotic | 43 | 2 | 22 |
| Otu133 | trnL | Magnoliopsida | Gentianales | Rubiaceae | Asperula |  | Forb | Likely Native | 22 | 1 | 22 |
| Otu134 | trnL | Magnoliopsida | Poales | Poaceae | Rytidosperma |  | Grass | Likely Native | 36 | 2 | 18 |
| Otu139 | trnL | Magnoliopsida | Ericales | Theaceae | Camelia |  | Shrub or Tree | Likely Exotic | 66 | 1 | 66 |
| Otu145 | trnL | Magnoliopsida | Caryophyllales | Polygonaceae | Rumex |  | Forb | Likely Native | 16 | 1 | 16 |
| Otu150 | trnL | Magnoliopsida | Gentianales | Rubiaceae |  |  | Forb | Unknown | 28 | 1 | 28 |
| Otu155 | trnL | Magnoliopsida | Brassicales | Brassicaceae | Cardamine |  | Forb | Likely Native | 24 | 1 | 24 |
| Otu163 | trnL | Magnoliopsida | Myrtales | Myrtaceae |  |  | Forb | Unknown | 20 | 2 | 10 |
| Otu173 | trnL | Magnoliopsida | Solanales | Convulvulaceae | Dichondra |  | Forb | Likely Native | 47 | 1 | 47 |
| Otu174 | trnL | Magnoliopsida | Gentianales | Rubiaceae |  |  | Forb | Unknown | 609 | 7 | 87 |
| Otu175 | trnL | Magnoliopsida | Myrtales | Myrtaceae | Eucalyptus |  | Shrub or Tree | Likely Native | 924 | 12 | 77 |
| Otu190 | trnL | Magnoliopsida | Malpighiales | Violaceae | Viola |  | Forb | Likely Native | 44 | 1 | 44 |
| Otu193 | trnL | Magnoliopsida | Myrtales | Myrtaceae |  |  | Shrub or Tree | Unknown | 57 | 2 | 29 |
| Otu194 | trnL | Magnoliopsida | Apiales | Araliaceae | Polyscias | sambucifolia | Shrub or Tree | Likely Native | 27 | 2 | 14 |
| Otu195 | trnL | Magnoliopsida | Rosales | Rosaceae |  |  | Forb | Unknown | 36 | 2 | 18 |
| Otu201 | trnL | Magnoliopsida | Fabales | Fabaceae | Mirbelia | oxylobioides | Shrub or Tree | Likely Native | 19 | 1 | 19 |
| Otu205 | trnL | Magnoliopsida | Poales | Cyperaceae | Carex |  | Grass-like Plants | Likely Native | 51 | 2 | 26 |
| Otu209 | trnL | Magnoliopsida | Myrtales | Myrtaceae |  |  | Shrub or Tree | Unknown | 14 | 1 | 14 |
| Otu217 | trnL | Magnoliopsida | Proteales | Proteaceae | Orites | lancifolius | Shrub or Tree | Likely Native | 11 | 1 | 11 |
| Otu218 | trnL | Magnoliopsida | Rosales | Ulmaceae | Ulmus |  | Shrub or Tree | Likely Exotic | 33 | 1 | 33 |
| Otu221 | trnL | Polypodiopsida | Polypodiales | Blechnaceae | Blechnum |  | Fern | Likely Native | 23 | 1 | 23 |
| Otu222 | trnL | Magnoliopsida | Asterales | Asteraceae | Celmisia | costiniana | Forb | Likely Native | 2278 | 15 | 152 |
| Otu231 | trnL | Magnoliopsida | Brassicales | Brassicaceae | Cardamine |  | Forb | Likely Native | 29 | 1 | 29 |
| Otu235 | trnL | Polypodiopsida | Polypodiales | Blechnaceae | Blechnum | penna-marina | Fern | Likely Native | 10 | 1 | 10 |
| Otu236 | trnL | Magnoliopsida | Poales | Cyperaceae | Carex |  | Grass-like Plants | Likely Native | 17 | 1 | 17 |
| Otu243 | trnL | Magnoliopsida | Poales | Restionaceae |  |  | Grass-like Plants | Unknown | 27 | 2 | 14 |
| Otu250 | trnL | Magnoliopsida | Brassicales | Brassicaceae |  |  | Forb | Unknown | 27 | 1 | 27 |
| Otu259 | trnL | Magnoliopsida | Gentianales | Rubiaceae |  |  | Forb | Unknown | 13 | 1 | 13 |
| Otu262 | trnL | Magnoliopsida | Asterales | Asteraceae | Erigeron |  | Forb | Likely Exotic | 22 | 1 | 22 |
| Otu263 | trnL | Magnoliopsida | Gentianales | Rubiaceae |  |  | Forb | Unknown | 10 | 1 | 10 |
| Otu277 | trnL | Magnoliopsida | Poales | Poaceae | Trisetum | spicatum | Grass | Likely Native | 17 | 1 | 17 |
| Otu283 | trnL | Magnoliopsida | Asterales | Asteraceae |  |  | Shrub or Tree | Unknown | 23 | 1 | 23 |
| Otu284 | trnL | Magnoliopsida | Saxifragales | Haloragaceae | Myriophyllum |  | Forb | Likely Native | 14 | 1 | 14 |
| Otu285 | trnL | Magnoliopsida | Myrtales | Myrtaceae |  |  | Shrub or Tree | Unknown | 34 | 2 | 17 |
| Otu304 | trnL | Magnoliopsida | Poales | Poaceae | Cenchrus | clandestinus | Grass | Likely Exotic | 16 | 1 | 16 |
| Otu331 | trnL | Magnoliopsida | Asterales | Asteraceae |  |  | Forb | Unknown | 1075 | 9 | 119 |
| Otu338 | trnL | Magnoliopsida | Asterales | Asteraceae | Gazania |  | Forb | Likely Exotic | 13 | 1 | 13 |
| Otu348 | trnL | Magnoliopsida | Asterales | Asteraceae | Lactuca | serriola | Forb | Likely Exotic | 18 | 1 | 18 |
| Otu360 | trnL | Magnoliopsida | Myrtales | Myrtaceae |  |  | Shrub or Tree | Unknown | 211 | 8 | 26 |
| Otu364 | trnL | Magnoliopsida | Rosales | Rosaceae | Sanguisorba |  | Forb | Likely Exotic | 21 | 2 | 11 |
| Otu370 | trnL | Magnoliopsida | Malpighiales | Violaceae | Melicytus |  | Shrub or Tree | Likely Native | 10 | 1 | 10 |
| Otu373 | trnL | Magnoliopsida | Rosales | Rosaceae |  |  | Shrub or Tree | Unknown | 169 | 6 | 28 |
| Otu376 | trnL | Magnoliopsida | Asterales | Asteraceae | Celmisia |  | Forb | Likely Native | 13 | 1 | 13 |
| Otu387 | trnL | Magnoliopsida | Myrtales | Myrtaceae |  |  | Shrub or Tree | Unknown | 378 | 9 | 42 |
| Otu395 | trnL | Magnoliopsida | Myrtales | Myrtaceae |  |  | Shrub or Tree | Unknown | 563 | 11 | 51 |
| Otu400 | trnL | Magnoliopsida | Asterales | Asteraceae |  |  | Shrub or Tree | Unknown | 12 | 1 | 12 |
| Otu409 | trnL | Magnoliopsida | Lamiales | Lamiaceae | Prostanthera |  | Shrub or Tree | Likely Native | 27 | 1 | 27 |
| Otu410 | trnL | Magnoliopsida | Myrtales | Myrtaceae |  |  | Shrub or Tree | Unknown | 12 | 1 | 12 |
| Otu418 | trnL | Pinopsida | Pinales | Cupressaceae | Callitris | glaucophylla | Shrub or Tree | Likely Exotic | 15 | 1 | 15 |
| Otu419 | trnL | Magnoliopsida | Rosales | Rosaceae | Potentilla | recta | Forb | Likely Exotic | 54 | 2 | 27 |
| Otu430 | trnL | Magnoliopsida | Myrtales | Myrtaceae |  |  | Shrub or Tree | Unknown | 583 | 11 | 53 |
| Otu457 | trnL | Magnoliopsida | Myrtales | Myrtaceae |  |  | Shrub or Tree | Unknown | 541 | 10 | 54 |
| Otu482 | trnL | Magnoliopsida | Myrtales | Myrtaceae |  |  | Shrub or Tree | Unknown | 347 | 10 | 35 |
| Otu484 | trnL | Magnoliopsida | Poales | Cyperaceae | Carex |  | Grass-like Plants | Likely Native | 71 | 1 | 71 |
| Otu501 | trnL | Magnoliopsida | Apiales | Apiaceae |  |  | Forb | Unknown | 283 | 1 | 283 |
| Otu507 | trnL | Magnoliopsida | Myrtales | Myrtaceae |  |  | Shrub or Tree | Unknown | 937 | 12 | 78 |
| Otu514 | trnL | Magnoliopsida | Gentianales | Rubiaceae | Galium |  | Forb | Likely Native | 192 | 4 | 48 |
| Otu521 | trnL | Magnoliopsida | Rosales | Rosaceae | Acaena |  | Forb | Likely Native | 183 | 4 | 46 |
| Otu546 | trnL | Magnoliopsida | Myrtales | Myrtaceae |  |  | Shrub or Tree | Unknown | 396 | 10 | 40 |
| Otu569 | trnL | Magnoliopsida | Myrtales | Myrtaceae |  |  | Shrub or Tree | Unknown | 378 | 10 | 38 |
| Otu582 | trnL | Magnoliopsida | Myrtales | Myrtaceae |  |  | Shrub or Tree | Unknown | 336 | 8 | 42 |
| Otu596 | trnL | Magnoliopsida | Myrtales | Myrtaceae |  |  | Shrub or Tree | Unknown | 23 | 2 | 12 |
| Otu620 | trnL | Magnoliopsida | Myrtales | Myrtaceae |  |  | Shrub or Tree | Unknown | 571 | 11 | 52 |
| Otu624 | trnL | Magnoliopsida | Myrtales | Myrtaceae |  |  | Shrub or Tree | Unknown | 359 | 8 | 45 |
| Otu631 | trnL | Magnoliopsida | Gentianales | Rubiaceae |  |  | Forb | Unknown | 24 | 1 | 24 |
| Otu644 | trnL | Magnoliopsida | Myrtales | Myrtaceae |  |  | Shrub or Tree | Unknown | 247 | 7 | 35 |
| Otu648 | trnL | Magnoliopsida | Gentianales | Rubiaceae |  |  | Forb | Unknown | 621 | 7 | 89 |
| Otu667 | trnL | Magnoliopsida | Myrtales | Myrtaceae |  |  | Shrub or Tree | Unknown | 460 | 11 | 42 |
| Otu677 | trnL | Magnoliopsida | Myrtales | Myrtaceae |  |  | Shrub or Tree | Unknown | 61 | 3 | 20 |
| Otu721 | trnL | Magnoliopsida | Myrtales | Myrtaceae |  |  | Shrub or Tree | Unknown | 310 | 10 | 31 |
| Otu731 | trnL | Magnoliopsida | Myrtales | Myrtaceae |  |  | Shrub or Tree | Unknown | 320 | 10 | 32 |
| Otu747 | trnL | Magnoliopsida | Asterales | Asteraceae | Erigeron |  | Forb | Likely Exotic | 10 | 1 | 10 |
| Otu802 | trnL | Magnoliopsida | Rosales | Rosaceae | Sanguisorba |  | Forb | Likely Exotic | 123 | 4 | 31 |
| Otu816 | trnL | Magnoliopsida | Lamiales | Lamiaceae | Prostanthera |  | Shrub or Tree | Likely Native | 217 | 1 | 217 |
| Otu909 | trnL | Magnoliopsida | Rosales | Rosaceae | Acaena |  | Forb | Likely Native | 525 | 6 | 88 |
| Otu970 | trnL | Magnoliopsida | Gentianales | Rubiaceae |  |  | Forb | Unknown | 66 | 2 | 33 |
| Otu990 | trnL | Magnoliopsida | Myrtales | Myrtaceae |  |  | Shrub or Tree | Unknown | 256 | 7 | 37 |
| Otu1036 | trnL | Magnoliopsida | Gentianales | Rubiaceae |  |  | Forb | Unknown | 193 | 6 | 32 |
| Otu1107 | trnL | Magnoliopsida | Myrtales | Myrtaceae |  |  | Shrub or Tree | Unknown | 341 | 10 | 34 |
| Otu1118 | trnL | Magnoliopsida | Rosales | Rosaceae |  |  | Shrub or Tree | Unknown | 82 | 1 | 82 |
| Otu1135 | trnL | Magnoliopsida | Myrtales | Myrtaceae |  |  | Shrub or Tree | Unknown | 42 | 4 | 11 |
| Otu1153 | trnL | Magnoliopsida | Gentianales | Rubiaceae |  |  | Forb | Unknown | 21 | 2 | 11 |
| Otu1300 | trnL | Magnoliopsida | Myrtales | Onagraceae | Epilobium |  | Forb | Likely Native | 44 | 1 | 44 |
| Otu1331 | trnL | Magnoliopsida | Myrtales | Myrtaceae |  |  | Shrub or Tree | Unknown | 125 | 7 | 18 |
| Otu1371 | trnL | Magnoliopsida | Myrtales | Myrtaceae |  |  | Shrub or Tree | Unknown | 33 | 3 | 11 |
| Otu1815 | trnL | Magnoliopsida | Gentianales | Rubiaceae |  |  | Forb | Unknown | 11 | 1 | 11 |
| Otu2258 | trnL | Magnoliopsida | Rosales | Rosaceae |  |  | Shrub or Tree | Unknown | 10 | 1 | 10 |
| Otu9 | ITS2 | Magnoliopsida | Rosales | Rosaceae | Acaena |  | Forb | Likely Native | 43406 | 16 | 2713 |
| Otu11 | ITS2 | Magnoliopsida | Myrtales | Myrtaceae | Eucalyptus |  | Shrub or Tree | Likely Native | 20298 | 15 | 1353 |
| Otu12 | ITS2 | Magnoliopsida | Saxifragales | Haloragaceae | Gonocarpus |  | Forb | Likely Native | 24513 | 13 | 1886 |
| Otu13 | ITS2 | Magnoliopsida | Asterales | Stylidiaceae | Stylidium | graminifolium | Forb | Likely Native | 11900 | 5 | 2380 |
| Otu14 | ITS2 | Magnoliopsida | Caryophyllales | Polygonaceae | Acetosella | vulgaris | Forb | Likely Exotic | 28813 | 16 | 1801 |
| Otu15 | ITS2 | Magnoliopsida | Asterales | Asteraceae | Cotula |  | Forb | Likely Native | 6711 | 1 | 6711 |
| Otu17 | ITS2 | Magnoliopsida | Asterales | Asteraceae | Hypochaeris | radicata | Forb | Likely Exotic | 2326 | 6 | 388 |
| Otu19 | ITS2 | Magnoliopsida | Sapindales | Rutaceae | Phebalium | Squamulosum | Shrub or Tree | Likely Native | 2577 | 9 | 286 |
| Otu29 | ITS2 | Magnoliopsida | Sapindales | Rutaceae | Asterolasia |  | Shrub or Tree | Likely Native | 1133 | 2 | 567 |
| Otu31 | ITS2 | Magnoliopsida | Rosales | Rosaceae | Rubus |  | Shrub or Tree | Likely Exotic | 2512 | 2 | 1256 |
| Otu36 | ITS2 | Magnoliopsida | Poales | Poaceae | Poa |  | Grass | Likely Native | 1066 | 13 | 82 |
| Otu41 | ITS2 | Magnoliopsida | Myrtales | Onagraceae | Epilobium |  | Forb | Likely Native | 1950 | 6 | 325 |
| Otu45 | ITS2 | Magnoliopsida | Malpighiales | Salicaceae | Salix | cinerea | Shrub or Tree | Likely Exotic | 1050 | 2 | 525 |
| Otu47 | ITS2 | Pinopsida | Pinales | Podocarpaceae | Podocarpus | lawrencei | Shrub or Tree | Likely Native | 567 | 1 | 567 |
| Otu50 | ITS2 | Magnoliopsida | Apiales | Araliaceae | Hydrocotyle |  | Forb | Likely Native | 529 | 1 | 529 |
| Otu52 | ITS2 | Magnoliopsida | Fabales | Fabaceae | Bossiaea | foliosa | Shrub or Tree | Likely Native | 618 | 6 | 103 |
| Otu54 | ITS2 | Magnoliopsida | Asterales | Asteraceae | Celmisia |  | Forb | Likely Native | 957 | 4 | 239 |
| Otu56 | ITS2 | Magnoliopsida | Malpighiales | Violaceae | Melicytus | dentatus | Shrub or Tree | Likely Native | 757 | 6 | 126 |
| Otu58 | ITS2 | Magnoliopsida | Myrtales | Myrtaceae | Eucalyptus |  | Shrub or Tree | Likely Native | 26 | 2 | 13 |
| Otu61 | ITS2 | Magnoliopsida | Apiales | Apiaceae | Oreomyrrhis |  | Forb | Likely Native | 338 | 4 | 85 |
| Otu63 | ITS2 | Magnoliopsida | Gentianales | Rubiaceae | Coprosma | hirtella | Shrub or Tree | Likely Native | 2260 | 2 | 1130 |
| Otu68 | ITS2 | Magnoliopsida | Asterales | Asteraceae | Lagenophora |  | Forb | Likely Native | 173 | 1 | 173 |
| Otu70 | ITS2 | Magnoliopsida | Asterales | Asteraceae | Craspedia |  | Forb | Likely Native | 277 | 2 | 139 |
| Otu72 | ITS2 | Magnoliopsida | Asterales | Asteraceae | Olearia |  | Forb | Likely Native | 305 | 5 | 61 |
| Otu73 | ITS2 | Magnoliopsida | Lamiales | Lamiaceae | Prostanthera |  | Shrub or Tree | Likely Native | 275 | 2 | 138 |
| Otu75 | ITS2 | Magnoliopsida | Gentianales | Rubiaceae | Galium |  | Forb | Likely Native | 377 | 6 | 63 |
| Otu82 | ITS2 | Magnoliopsida | Caryophyllales | Caryophyllaceae | Stellaria |  | Forb | Likely Native | 198 | 4 | 50 |
| Otu87 | ITS2 | Magnoliopsida | Brassicales | Brassicaceae | Brassica | oleracea | Forb | Likely Exotic | 186 | 3 | 62 |
| Otu89 | ITS2 | Magnoliopsida | Caryophyllales | Caryophyllaceae | Scleranthus | biflorus | Forb | Likely Native | 175 | 3 | 58 |
| Otu90 | ITS2 | Magnoliopsida | Rosales | Urticaceae | Urtica | incisa | Forb | Likely Native | 138 | 1 | 138 |
| Otu91 | ITS2 | Magnoliopsida | Asterales | Asteraceae | Coronidium | monticola | Forb | Likely Native | 169 | 4 | 42 |
| Otu101 | ITS2 | Magnoliopsida | Fabales | Fabaceae | Oxylobium | ellipticum | Shrub or Tree | Likely Native | 142 | 2 | 71 |
| Otu105 | ITS2 | Magnoliopsida | Geraniales | Geraniaceae | Geranium |  | Forb | Likely Native | 129 | 1 | 129 |
| Otu109 | ITS2 | Magnoliopsida | Gentianales | Rubiaceae | Galium |  | Forb | Likely Native | 307 | 4 | 77 |
| Otu111 | ITS2 | Magnoliopsida | Saxifragales | Haloragaceae | Gonocarpus |  | Forb | Likely Native | 96 | 3 | 32 |
| Otu122 | ITS2 | Magnoliopsida | Myrtales | Myrtaceae | Baeckea |  | Shrub or Tree | Likely Native | 92 | 3 | 31 |
| Otu125 | ITS2 | Magnoliopsida | Malpighiales | Violaceae | Viola |  | Forb | Likely Native | 184 | 4 | 46 |
| Otu133 | ITS2 | Magnoliopsida | Asterales | Asteraceae | Leptinella |  | Forb | Likely Native | 83 | 1 | 83 |
| Otu144 | ITS2 | Magnoliopsida | Fabales | Fabaceae | Hovea |  | Shrub or Tree | Likely Native | 32 | 1 | 32 |
| Otu149 | ITS2 | Magnoliopsida | Asparagales | Asphodelaceae | Dianella | tasmanica | Grass-like Plants | Likely Native | 34 | 2 | 17 |
| Otu153 | ITS2 | Magnoliopsida | Malvales | Thymelaeaeae | Pimelea | axiflora subsp. alpina | Shrub or Tree | Likely Native | 58 | 1 | 58 |
| Otu157 | ITS2 | Magnoliopsida | Malvales | Thymelaeaeae | Pimelea | alpina | Shrub or Tree | Likely Native | 51 | 3 | 17 |
| Otu158 | ITS2 | Magnoliopsida | Fabales | Fabaceae | Podolobium | alpestre | Shrub or Tree | Likely Native | 49 | 2 | 25 |
| Otu166 | ITS2 | Magnoliopsida | Asterales | Stylidiaceae | Stylidium |  | Forb | Likely Native | 33 | 1 | 33 |
| Otu168 | ITS2 | Magnoliopsida | Proteales | Proteaceae | Grevillea |  | Shrub or Tree | Likely Native | 36 | 3 | 12 |
| Otu173 | ITS2 | Magnoliopsida | Ranunculales | Ranunculaceae | Ranunculus |  | Forb | Likely Native | 58 | 2 | 29 |
| Otu180 | ITS2 | Magnoliopsida | Rosales | Rhamnaceae | Pomaderris |  | Shrub or Tree | Likely Native | 9 | 1 | 9 |
| Otu185 | ITS2 | Magnoliopsida | Ericales | Ericaceae | Acrothamnus |  | Shrub or Tree | Likely Native | 36 | 1 | 36 |
| Otu186 | ITS2 | Magnoliopsida | Asterales | Asteraceae | Senecio | pinnatifolius | Forb | Likely Native | 43 | 1 | 43 |
| Otu188 | ITS2 | Pinopsida | Pinales | Podocarpaceae | Podocarpus | lawrencei | Shrub or Tree | Likely Native | 33 | 1 | 33 |
| Otu190 | ITS2 | Magnoliopsida | Asterales | Asteraceae | Leptorhynchos | squamatus | Forb | Likely Native | 20 | 1 | 20 |
| Otu191 | ITS2 | Magnoliopsida | Caryophyllales | Montiaceae | Montia | australasica | Forb | Likely Native | 71 | 1 | 71 |
| Otu200 | ITS2 | Magnoliopsida | Malpighiales | Salicaceae | Salix | cinerea | Shrub or Tree | Likely Exotic | 9 | 1 | 9 |
| Otu202 | ITS2 | Magnoliopsida | Canellales | Winteraceae | Tasmannia |  | Shrub or Tree | Likely Native | 45 | 2 | 23 |
| Otu205 | ITS2 | Magnoliopsida | Asterales | Asteraceae | Leptinella |  | Forb | Likely Native | 44 | 1 | 44 |
| Otu216 | ITS2 | Magnoliopsida | Caryophyllales | Polygonaceae | Rumex |  | Forb | Likely Exotic | 14 | 1 | 14 |
| Otu217 | ITS2 | Magnoliopsida | Asterales | Stylidiaceae | Stylidium |  | Forb | Likely Native | 47 | 1 | 47 |
| Otu223 | ITS2 | Magnoliopsida | Asterales | Asteraceae | Leptinella |  | Forb | Likely Native | 26 | 1 | 26 |
| Otu225 | ITS2 | Magnoliopsida | Asterales | Asteraceae | Microseris | lanceolata | Forb | Likely Native | 58 | 3 | 19 |
| Otu231 | ITS2 | Magnoliopsida | Caryophyllales | Caryophyllaceae | Cerastium |  | Forb | Likely Exotic | 25 | 1 | 25 |
| Otu241 | ITS2 | Magnoliopsida | Asparagales | Amarylidaceae | Allium |  | Forb | Likely Exotic | 14 | 1 | 14 |
| Otu248 | ITS2 | Magnoliopsida | Poales | Juncaceae | Luzula |  | Grass-like Plants | Likely Native | 8 | 1 | 8 |
| Otu250 | ITS2 | Magnoliopsida | Solanales | Convulvulaceae | Dichondra | repens | Forb | Likely Native | 19 | 1 | 19 |
| Otu252 | ITS2 | Bryopsida | Dicranales | Ditrichaceae | Ceratodon | purpureus | Mosses | Unknown | 78 | 2 | 39 |
| Otu253 | ITS2 | Bryopsida | Pottiales | Pottiaceae | Oncophorus | elongatus | Mosses | Unknown | 14 | 1 | 14 |
| Otu258 | ITS2 | Magnoliopsida | Saxifragales | Haloragaceae | Gonocarpus |  | Forb | Likely Native | 12 | 1 | 12 |
| Otu270 | ITS2 | Bryopsida | Bryales | Mniaceae | Pohlia | nutans | Mosses | Unknown | 22 | 1 | 22 |
| Otu272 | ITS2 | Magnoliopsida | Asterales | Stylidiaceae | Stylidium |  | Forb | Likely Native | 18 | 1 | 18 |
| Otu277 | ITS2 | Magnoliopsida | Rosales | Rosaceae | Rubus |  | Shrub or Tree | Likely Exotic | 19 | 2 | 10 |
| Otu280 | ITS2 | Magnoliopsida | Asterales | Asteraceae | Leptinella | filicula | Forb | Likely Native | 12 | 1 | 12 |
| Otu284 | ITS2 | Magnoliopsida | Caryophyllales | Polygonaceae | Rumex |  | Forb | Likely Exotic | 21 | 1 | 21 |
| Otu291 | ITS2 | Magnoliopsida | Saxifragales | Haloragaceae | Gonocarpus |  | Forb | Likely Native | 54 | 2 | 27 |
| Otu293 | ITS2 | Magnoliopsida | Asterales | Asteraceae | Brachyscome | decipiens | Forb | Likely Native | 10 | 1 | 10 |
| Otu295 | ITS2 | Magnoliopsida | Poales | Cyperaceae | Carex |  | Grass-like Plants | Likely Native | 21 | 2 | 11 |
| Otu300 | ITS2 | Magnoliopsida | Apiales | Araliaceae | Hydrocotyle |  | Forb | Likely Native | 19 | 1 | 19 |
| Otu301 | ITS2 | Magnoliopsida | Rosales | Rosaceae | Acaena |  | Forb | Likely Native | 14 | 1 | 14 |
| Otu308 | ITS2 | Magnoliopsida | Saxifragales | Haloragaceae | Gonocarpus | teucrioides | Forb | Likely Native | 9 | 1 | 9 |
| Otu317 | ITS2 | Magnoliopsida | Rosales | Rosaceae | Acaena |  | Forb | Likely Native | 5 | 1 | 5 |
| Otu320 | ITS2 | Magnoliopsida | Caryophyllales | Polygonaceae | Rumex |  | Forb | Likely Exotic | 10 | 1 | 10 |
| Otu321 | ITS2 | Magnoliopsida | Asterales | Stylidiaceae |  |  | Forb | Unknown | 16 | 1 | 16 |
| Otu325 | ITS2 | Magnoliopsida | Caryophyllales | Polygonaceae |  |  | Forb | Unknown | 17 | 1 | 17 |
| Otu330 | ITS2 | Magnoliopsida | Caryophyllales | Polygonaceae | Rumex |  | Forb | Likely Exotic | 8 | 1 | 8 |
| Otu333 | ITS2 | Magnoliopsida | Asterales | Asteraceae |  |  | Forb | Unknown | 37 | 1 | 37 |
| Otu341 | ITS2 | Magnoliopsida | Asterales | Stylidiaceae | Stylidium |  | Forb | Likely Native | 12 | 1 | 12 |
| Otu351 | ITS2 | Magnoliopsida | Asterales | Asteraceae |  |  | Forb | Unknown | 9 | 1 | 9 |
| Otu353 | ITS2 | Magnoliopsida | Asterales | Asteraceae | Ozothamnus |  | Shrub or Tree | Likely Native | 10 | 1 | 10 |
| Otu355 | ITS2 | Magnoliopsida | Caryophyllales | Polygonaceae | Rumex |  | Forb | Likely Exotic | 7 | 1 | 7 |
| Otu361 | ITS2 | Bryopsida | Hypnales | Brachytheciaceae | Brachytheciastrum | | Mosses | Unknown | 6 | 1 | 6 |
| Otu364 | ITS2 | Magnoliopsida | Asterales | Asteraceae |  |  | Shrub or Tree | Unknown | 35 | 1 | 35 |
| Otu381 | ITS2 | Magnoliopsida | Asterales | Asteraceae | Celmisia |  | Forb | Likely Native | 361 | 4 | 90 |
| Otu384 | ITS2 | Magnoliopsida | Caryophyllales | Polygonaceae | Rumex |  | Forb | Likely Exotic | 25 | 1 | 25 |
| Otu386 | ITS2 | Magnoliopsida | Gentianales | Rubiaceae | Coprosma |  | Shrub or Tree | Likely Native | 9 | 1 | 9 |
| Otu387 | ITS2 | Magnoliopsida | Saxifragales | Haloragaceae | Gonocarpus |  | Forb | Likely Native | 6 | 1 | 6 |
| Otu399 | ITS2 | Magnoliopsida | Rosales | Rosaceae | Acaena |  | Forb | Likely Native | 82 | 1 | 82 |
| Otu401 | ITS2 | Magnoliopsida | Myrtales | Myrtaceae | Lophostemon | confertus | Shrub or Tree | Likely Exotic | 7 | 1 | 7 |
| Otu402 | ITS2 | Magnoliopsida | Rosales | Rosaceae | Acaena |  | Forb | Likely Native | 13 | 1 | 13 |
| Otu406 | ITS2 | Magnoliopsida | Rosales | Rosaceae |  |  | Shrub or Tree | Unknown | 12 | 1 | 12 |
| Otu408 | ITS2 | Magnoliopsida | Gentianales | Rubiaceae | Coprosma |  | Shrub or Tree | Likely Native | 5 | 1 | 5 |
| Otu419 | ITS2 | Magnoliopsida | Caryophyllales | Polygonaceae | Rumex |  | Forb | Likely Exotic | 7 | 1 | 7 |
| Otu427 | ITS2 | Magnoliopsida | Asterales | Asteraceae |  |  | Shrub or Tree | Unknown | 6 | 1 | 6 |
| Otu432 | ITS2 | Pinopsida | Pinales | Podocarpaceae | Podocarpus |  | Shrub or Tree | Likely Native | 7 | 1 | 7 |
| Otu439 | ITS2 | Magnoliopsida | Rosales | Rosaceae |  |  | Forb | Unknown | 10 | 1 | 10 |
| Otu444 | ITS2 | Magnoliopsida | Asterales | Goodeniaceae | Goodenia |  | Shrub or Tree | Likely Native | 10 | 1 | 10 |
| Otu450 | ITS2 | Magnoliopsida | Caryophyllales | Polygonaceae | Rumex |  | Forb | Likely Exotic | 9 | 1 | 9 |
| Otu452 | ITS2 | Magnoliopsida | Lamiales | Lamiaceae | Mentha |  | Forb | Likely Exotic | 6 | 1 | 6 |
| Otu460 | ITS2 | Magnoliopsida | Asterales | Asteraceae | Cirsium | vulgare | Forb | Likely Exotic | 8 | 1 | 8 |
| Otu464 | ITS2 | Magnoliopsida | Caryophyllales | Polygonaceae | Rumex |  | Forb | Likely Exotic | 11 | 1 | 11 |
| Otu466 | ITS2 | Magnoliopsida | Caryophyllales | Polygonaceae | Rumex |  | Forb | Likely Exotic | 9 | 1 | 9 |
| Otu470 | ITS2 | Magnoliopsida | Gentianales | Rubiaceae | Coprosma |  | Shrub or Tree | Likely Native | 9 | 1 | 9 |
| Otu476 | ITS2 | Magnoliopsida | Gentianales | Rubiaceae | Galium |  | Forb | Likely Native | 178 | 4 | 45 |
| Otu479 | ITS2 | Magnoliopsida | Gentianales | Rubiaceae | Galium |  | Forb | Likely Native | 24 | 3 | 8 |
| Otu481 | ITS2 | Magnoliopsida | Laurales | Monimiaceae |  |  | Shrub or Tree | Unknown | 5 | 1 | 5 |
| Otu484 | ITS2 | Pinopsida | Pinales | Podocarpaceae | Podocarpus |  | Shrub or Tree | Likely Native | 6 | 1 | 6 |
| Otu491 | ITS2 | Magnoliopsida | Asterales | Asteraceae |  |  | Shrub or Tree | Unknown | 13 | 1 | 13 |
| Otu504 | ITS2 | Magnoliopsida | Asterales | Stylidiaceae | Stylidium |  | Forb | Likely Native | 9 | 1 | 9 |
| Otu507 | ITS2 | Magnoliopsida | Asterales | Stylidiaceae | Stylidium |  | Forb | Likely Native | 5 | 1 | 5 |
| Otu519 | ITS2 | Magnoliopsida | Asterales | Asteraceae | Olearia |  | Forb | Likely Native | 62 | 1 | 62 |
| Otu536 | ITS2 | Jungermanniopsida | Jungermanniales | Lophocoleaceae | Chiloscyphus |  | Liverworts | Unknown | 5 | 1 | 5 |
| Otu544 | ITS2 | Magnoliopsida | Apiales | Apiaceae |  |  | Forb | Unknown | 13 | 1 | 13 |
| Otu550 | ITS2 | Magnoliopsida | Caryophyllales | Polygonaceae | Rumex |  | Forb | Likely Exotic | 38 | 1 | 38 |
| Otu552 | ITS2 | Magnoliopsida | Asterales | Asteraceae |  |  | Shrub or Tree | Unknown | 34 | 1 | 34 |
| Otu570 | ITS2 | Pinopsida | Pinales | Podocarpaceae |  |  | Shrub or Tree | Unknown | 24 | 1 | 24 |
| Otu597 | ITS2 | Magnoliopsida | Poales | Poaceae | Anthosachne |  | Grass | Likely Native | 5 | 1 | 5 |
| Otu1 | rbcLFWD | Magnoliopsida | Myrtales | Myrtaceae | Eucalyptus |  | Shrub or Tree | Likely Native | 178331 | 12 | 14861 |
| Otu1 | rbcLREV | Magnoliopsida | Myrtales | Myrtaceae | Eucalyptus |  | Shrub or Tree | Likely Native | 143612 | 12 | 11968 |
| Otu2 | rbcLFWD | Magnoliopsida | Saxifragales | Haloragaceae | Myriophyllum |  | Forb | Likely Native | 71891 | 9 | 7988 |
| Otu2 | rbcLREV | Magnoliopsida | Saxifragales | Haloragaceae | Myriophyllum |  | Forb | Likely Native | 64354 | 9 | 7150 |
| Otu3 | rbcLREV | Magnoliopsida | Malpighiales | Violaceae | Viola |  | Forb | Likely Native | 33158 | 1 | 33158 |
| Otu3 | rbcLFWD | Magnoliopsida | Rosales | Rosaceae | Acaena | novae-zelandiae | Forb | Likely Native | 27120 | 6 | 4520 |
| Otu4 | rbcLFWD | Magnoliopsida | Caryophyllales | Polygonaceae | Acetosella | vulgaris | Forb | Likely Exotic | 31290 | 10 | 3129 |
| Otu4 | rbcLREV | Magnoliopsida | Rosales | Rosaceae | Acaena | novae-zelandiae | Forb | Likely Native | 22876 | 6 | 3813 |
| Otu5 | rbcLFWD | Magnoliopsida | Asterales | Stylidiaceae | Stylidium | graminifolium | Forb | Likely Native | 32726 | 2 | 16363 |
| Otu5 | rbcLREV | Magnoliopsida | Asterales | Stylidiaceae | Stylidium | graminifolium | Forb | Likely Native | 15487 | 2 | 7744 |
| Otu6 | rbcLFWD | Magnoliopsida | Malpighiales | Violaceae | Viola |  | Forb | Likely Native | 36690 | 1 | 36690 |
| Otu6 | rbcLREV | Jungermanniopsida | Jungermanniales | Cephaloziellaceae | Cephaloziella | spinicaulis | Mosses and Liverworts | Unknown | 12249 | 1 | 12249 |
| Otu7 | rbcLREV | Magnoliopsida | Fabales | Fabaceae | Trifolium | repens | Forb | Likely Exotic | 17455 | 2 | 8728 |
| Otu7 | rbcLFWD | Magnoliopsida | Gentianales | Rubiaceae | Galium |  | Forb | Likely Native | 15962 | 4 | 3991 |
| Otu8 | rbcLFWD | Magnoliopsida | Poales | Poaceae | Poa |  | Grass | Likely Native | 14699 | 6 | 2450 |
| Otu9 | rbcLREV | Magnoliopsida | Gentianales | Rubiaceae | Galium |  | Forb | Likely Native | 13290 | 4 | 3323 |
| Otu9 | rbcLFWD | Jungermanniopsida | Jungermanniales | Cephaloziellaceae | Cephaloziella | spinicaulis | Mosses and Liverworts | Unknown | 13352 | 1 | 13352 |
| Otu10 | rbcLFWD | Magnoliopsida | Asterales | Asteraceae | Cotula | australis | Forb | Unknown | 11175 | 5 | 2235 |
| Otu10 | rbcLREV | Magnoliopsida | Asterales | Asteraceae | Cotula | australis | Forb | Unknown | 9071 | 2 | 4536 |
| Otu11 | rbcLFWD | Magnoliopsida | Fabales | Fabaceae | Trifolium | repens | Forb | Likely Exotic | 10092 | 1 | 10092 |
| Otu12 | rbcLREV | Bryopsida | Dicranidales | Ditrichaceae | Ceratodon | purpureus | Mosses and Liverworts | Unknown | 6166 | 3 | 2055 |
| Otu12 | rbcLFWD | Bryopsida | Fissidentales | Fissidentaceae | Ditrichum | flexicaule | Mosses and Liverworts | Unknown | 6789 | 2 | 3395 |
| Otu13 | rbcLREV | Magnoliopsida | Caryophyllales | Polygonaceae | Acetosella | vulgaris | Forb | Likely Exotic | 19009 | 10 | 1901 |
| Otu13 | rbcLFWD | Magnoliopsida | Solanales | Solanaceae | Solanum |  | Shrub or Tree | Likely Exotic | 8144 | 1 | 8144 |
| Otu14 | rbcLREV | Magnoliopsida | Poales | Poaceae | Cenchrus |  | Grass | Likely Exotic | 9466 | 1 | 9466 |
| Otu15 | rbcLREV | Magnoliopsida | Poales | Poaceae | Poa |  | Grass | Likely Native | 11057 | 6 | 1843 |
| Otu15 | rbcLFWD | Magnoliopsida | Poales | Poaceae |  |  | Grass | Unknown | 10924 | 1 | 10924 |
| Otu16 | rbcLFWD | Magnoliopsida | Fagales | Casuarinaceae | Allocasuarina | verticillata | Shrub or Tree | Likely Exotic | 5320 | 1 | 5320 |
| Otu16 | rbcLREV | Magnoliopsida | Solanales | Solanaceae | Solanum |  | Shrub or Tree | Likely Exotic | 7011 | 1 | 7011 |
| Otu17 | rbcLREV | Magnoliopsida | Fagales | Casuarinaceae | Allocasuarina | verticillata | Shrub or Tree | Likely Exotic | 4483 | 1 | 4483 |
| Otu18 | rbcLFWD | Magnoliopsida | Gentianales | Rubiaceae | Coprosma | hirtella | Shrub or Tree | Likely Native | 3823 | 1 | 3823 |
| Otu18 | rbcLREV | Magnoliopsida | Gentianales | Rubiaceae | Coprosma | hirtella | Shrub or Tree | Likely Native | 3357 | 1 | 3357 |
| Otu19 | rbcLREV | Magnoliopsida | Malpighiales | Violaceae | Melicytus | dentatus | Shrub or Tree | Likely Native | 2076 | 2 | 1038 |
| Otu19 | rbcLFWD | Magnoliopsida | Rosales | Urticaceae | Australina | pusilla | Forb | Likely Native | 2992 | 1 | 2992 |
| Otu20 | rbcLFWD | Magnoliopsida | Caryophyllales | Caryophyllaceae | Scleranthus |  | Forb | Likely Native | 3413 | 1 | 3413 |
| Otu21 | rbcLREV | Magnoliopsida | Sapindales | Rutaceae | Asterolasia | asteriscophora | Shrub or Tree | Likely Native | 1551 | 1 | 1551 |
| Otu22 | rbcLFWD | Magnoliopsida | Malpighiales | Violaceae | Melicytus | dentatus | Shrub or Tree | Likely Native | 2349 | 2 | 1175 |
| Otu24 | rbcLREV | Magnoliopsida | Caryophyllales | Caryophyllaceae | Scleranthus |  | Forb | Likely Native | 2815 | 1 | 2815 |
| Otu25 | rbcLFWD | Magnoliopsida | Fabales | Fabaceae | Medicago |  | Forb | Likely Exotic | 10901 | 1 | 10901 |
| Otu25 | rbcLREV | Bryopsida | Bryales | Bryaceae |  |  | Mosses and Liverworts | Unknown | 1794 | 3 | 598 |
| Otu27 | rbcLFWD | Magnoliopsida | Sapindales | Rutaceae | Boronia |  | Shrub or Tree | Likely Native | 1702 | 2 | 851 |
| Otu27 | rbcLREV | Magnoliopsida | Rosales | Urticaceae | Australina | pusilla | Forb | Likely Native | 2455 | 1 | 2455 |
| Otu29 | rbcLREV | Magnoliopsida | Canellales | Winteraceae | Tasmannia |  | Shrub or Tree | Likely Native | 520 | 2 | 260 |
| Otu34 | rbcLFWD | Magnoliopsida | Asterales | Asteraceae | Hypochaeris | radicata | Forb | Likely Exotic | 299 | 1 | 299 |
| Otu34 | rbcLREV | Pinopsida | Pinales | Cupressaceae | Cupressus |  | Shrub or Tree | Likely Exotic | 458 | 1 | 458 |
| Otu35 | rbcLREV | Magnoliopsida | Rosales | Rosaceae | Rubus |  | Shrub or Tree | Likely Exotic | 825 | 1 | 825 |
| Otu35 | rbcLFWD | Magnoliopsida | Canellales | Winteraceae | Tasmannia |  | Shrub or Tree | Likely Native | 552 | 2 | 276 |
| Otu36 | rbcLFWD | Bryopsida | Bryales | Mniaceae | Pohlia | nutans | Mosses and Liverworts | Unknown | 550 | 1 | 550 |
| Otu38 | rbcLREV | Magnoliopsida | Laurales | Atherospermataceae | Atherosperma | moschatum | Shrub or Tree | Likely Native | 219 | 1 | 219 |
| Otu39 | rbcLREV | Magnoliopsida | Caryophyllales | Caryophyllaceae | Stellaria | media | Forb | Likely Exotic | 212 | 1 | 212 |
| Otu39 | rbcLFWD | Pinopsida | Pinales | Cupressaceae | Cupressus |  | Shrub or Tree | Likely Exotic | 475 | 1 | 475 |
| Otu41 | rbcLFWD | Polypodiopsida | Polypodiales | Dryopteridaceae | Polystichum |  | Fern | Likely Native | 349 | 1 | 349 |
| Otu41 | rbcLREV | Polypodiopsida | Polypodiales | Dryopteridaceae | Polystichum |  | Fern | Likely Native | 284 | 1 | 284 |
| Otu42 | rbcLFWD | Magnoliopsida | Poales | Poaceae | Anthosachne |  | Grass | Likely Native | 232 | 1 | 232 |
| Otu44 | rbcLFWD | Magnoliopsida | Caryophyllales | Caryophyllaceae | Stellaria | angustifolia | Forb | Likely Native | 235 | 1 | 235 |
| Otu45 | rbcLFWD | Magnoliopsida | Laurales | Atherospermataceae | Atherosperma | moschatum | Shrub or Tree | Likely Native | 235 | 1 | 235 |
| Otu45 | rbcLREV | Magnoliopsida | Poales | Poaceae | Rytidosperma |  | Grass | Likely Native | 98 | 1 | 98 |
| Otu46 | rbcLREV | Magnoliopsida | Myrtales | Onagraceae | Epilobium |  | Forb | Likely Native | 183 | 2 | 92 |
| Otu47 | rbcLFWD | Magnoliopsida | Myrtales | Onagraceae | Epilobium |  | Forb | Likely Native | 207 | 2 | 104 |
| Otu47 | rbcLREV | Bryopsida | Bryales | Bryaceae |  |  | Mosses and Liverworts | Unknown | 355 | 1 | 355 |
| Otu48 | rbcLFWD | Magnoliopsida | Apiales | Apiaceae | Daucus | glochidiatus | Forb | Likely Native | 138 | 1 | 138 |
| Otu49 | rbcLFWD | Magnoliopsida | Poales | Restionaceae | Empodisma | minus | Forb | Likely Native | 80 | 1 | 80 |
| Otu49 | rbcLREV | Magnoliopsida | Poales | Restionaceae | Empodisma | minus | Forb | Likely Native | 71 | 1 | 71 |
| Otu51 | rbcLREV | Magnoliopsida | Apiales | Apiaceae |  |  | Forb | Unknown | 113 | 1 | 113 |
| Otu54 | rbcLREV | Magnoliopsida | Poales | Poaceae | Anthosachne |  | Grass | Likely Native | 169 | 1 | 169 |
| Otu54 | rbcLFWD | Magnoliopsida | Poales | Poaceae | Rytidosperma | pallidum | Grass | Likely Native | 90 | 1 | 90 |
| Otu58 | rbcLFWD | Magnoliopsida | Geraniales | Geraniaceae | Geranium |  | Forb | Likely Native | 43 | 1 | 43 |
| Otu58 | rbcLREV | Magnoliopsida | Geraniales | Geraniaceae | Geranium |  | Forb | Likely Native | 40 | 1 | 40 |
| Otu59 | rbcLFWD | Magnoliopsida | Rosales | Rosaceae | Rubus |  | Shrub or Tree | Likely Exotic | 936 | 1 | 936 |
| Otu61 | rbcLFWD | Magnoliopsida | Asterales | Asteraceae |  |  | Shrub or Tree | Unknown | 3104 | 1 | 3104 |
| Otu65 | rbcLREV | Magnoliopsida | Asterales | Asteraceae |  |  | Forb | Unknown | 1048 | 4 | 262 |
| Otu67 | rbcLFWD | Bryopsida | Bryales | Bryaceae |  |  | Mosses and Liverworts | Unknown | 1404 | 2 | 702 |
| Otu68 | rbcLFWD | Bryopsida | Dicranales | Ditrichaceae |  |  | Mosses and Liverworts | Unknown | 344 | 1 | 344 |
| Otu73 | rbcLFWD | Bryopsida | Bryales | Bryaceae |  |  | Mosses and Liverworts | Unknown | 529 | 1 | 529 |
| Otu82 | rbcLREV | Magnoliopsida | Fabales | Fabaceae | Medicago |  | Forb | Likely Exotic | 111 | 1 | 111 |
| Otu84 | rbcLREV | Magnoliopsida | Fabales | Fabaceae | Medicago |  | Forb | Likely Exotic | 44 | 1 | 44 |
| Otu87 | rbcLREV | Magnoliopsida | Fabales | Fabaceae | Medicago |  | Forb | Likely Exotic | 273 | 1 | 273 |
| Otu92 | rbcLFWD | Magnoliopsida | Myrtales | Myrtaceae | Eucalyptus |  | Shrub or Tree | Likely Native | 267 | 4 | 67 |
| Otu125 | rbcLFWD | Magnoliopsida | Gentianales | Rubiaceae |  |  | Forb | Unknown | 39 | 1 | 39 |

**Table S4.** Taxonomic table displaying the assignment of OTUs in the Wet Forest samples. Sum refers to total number of sequence reads for each OTU. Count refers to the number of samples an OTU was detected in, and average read depth highlights the total reads on average for OTUs across detected samples.

| OTU ID | Locus | Class | Order | Family | Genus | Species | Growth Form | Origin | Sum | Count | Average Read Depth |
| --- | --- | --- | --- | --- | --- | --- | --- | --- | --- | --- | --- |
| Otu1 | trnL | Magnoliopsida | Canellales | Winteraceae | Tasmannia |  | Shrub or Tree | Likely Native | 192048 | 16 | 12003 |
| Otu2 | trnL | Magnoliopsida | Rosales | Rosaceae |  |  | Shrub or Tree | Unknown | 71870 | 19 | 3783 |
| Otu3 | trnL | Magnoliopsida | Poales | Poaceae | Microlaena | stipoides | Grass | Likely Native | 46700 | 17 | 2747 |
| Otu4 | trnL | Polypodiopsida | Polypodiales | Blechnaceae | Blechnum | cartilagineum | Fern | Likely Native | 27129 | 7 | 3876 |
| Otu5 | trnL | Magnoliopsida | Poales | Juncaceae | Juncus |  | Grass-like Plants | Likely Native | 27417 | 3 | 9139 |
| Otu7 | trnL | Polypodiopsida | Polypodiales | Dennstaedtiaceae | Pteridium | esculentum | Fern | Likely Native | 16117 | 13 | 1240 |
| Otu8 | trnL | Magnoliopsida | Poales | Poaceae | Anthoxanthum | odoratum | Grass | Likely Exotic | 21164 | 15 | 1411 |
| Otu9 | trnL | Magnoliopsida | Poales | Poaceae | Poa |  | Grass | Likely Native | 23188 | 17 | 1364 |
| Otu10 | trnL | Magnoliopsida | Gentianales | Rubiaceae |  |  | Forb | Unknown | 16695 | 12 | 1391 |
| Otu11 | trnL | Magnoliopsida | Myrtales | Myrtaceae |  |  | Shrub or Tree | Unknown | 35122 | 19 | 1849 |
| Otu12 | trnL | Magnoliopsida | Asterales | Asteraceae |  |  | Forb | Unknown | 14096 | 15 | 940 |
| Otu13 | trnL | Magnoliopsida | Rosales | Rosaceae | Acaena |  | Forb | Likely Native | 12737 | 18 | 708 |
| Otu14 | trnL | Magnoliopsida | Gentianales | Rubiaceae | Galium |  | Forb | Likely Native | 15971 | 13 | 1229 |
| Otu15 | trnL | Polypodiopsida | Cyatheales | Cyatheaceae | Cyathea |  | Fern | Likely Native | 8900 | 12 | 742 |
| Otu17 | trnL | Magnoliopsida | Caryophyllales | Polygonaceae | Acetosella | vulgaris | Forb | Likely Exotic | 7147 | 9 | 794 |
| Otu18 | trnL | Magnoliopsida | Fabales | Fabaceae | Lotus |  | Forb | Likely Exotic | 7055 | 13 | 543 |
| Otu19 | trnL | Magnoliopsida | Fabales | Fabaceae | Pultenaea |  | Shrub or Tree | Likely Native | 6256 | 6 | 1043 |
| Otu20 | trnL | Polypodiopsida | Polypodiales | Blechnaceae | Blechnum |  | Fern | Likely Native | 5304 | 11 | 482 |
| Otu21 | trnL | Magnoliopsida | Gentianales | Rubiaceae | Asperula |  | Forb | Likely Native | 8274 | 7 | 1182 |
| Otu22 | trnL | Magnoliopsida | Fagales | Fagaceae | Quercus | robur | Shrub or Tree | Likely Exotic | 4808 | 1 | 4808 |
| Otu23 | trnL | Magnoliopsida | Lamiales | Lamiaceae | Prostanthera |  | Shrub or Tree | Likely Native | 4720 | 12 | 393 |
| Otu24 | trnL | Magnoliopsida | Fabales | Fabaceae | Desmodium | gunnii | Forb | Likely Native | 3597 | 4 | 899 |
| Otu26 | trnL | Magnoliopsida | Lamiales | Plantaginaceae | Plantago | lanceolata | Forb | Likely Exotic | 4079 | 10 | 408 |
| Otu27 | trnL | Magnoliopsida | Oxalidales | Elaeocarpaceae | Tetratheca |  | Shrub or Tree | Likely Native | 3829 | 6 | 638 |
| Otu28 | trnL | Magnoliopsida | Poales | Poaceae | Ehrharta | erecta | Grass | Likely Exotic | 3050 | 2 | 1525 |
| Otu30 | trnL | Magnoliopsida | Apiales | Araliaceae | Hydrocotyle |  | Forb | Likely Native | 2797 | 3 | 932 |
| Otu31 | trnL | Magnoliopsida | Lamiales | Plantaginaceae | Gratiola |  | Forb | Likely Native | 2834 | 1 | 2834 |
| Otu32 | trnL | Magnoliopsida | Rosales | Rhamnaceae | Spyridium | parvifolium | Shrub or Tree | Likely Native | 2589 | 9 | 288 |
| Otu33 | trnL | Magnoliopsida | Geraniales | Geraniaceae | Geranium |  | Forb | Likely Native | 2797 | 17 | 165 |
| Otu34 | trnL | Magnoliopsida | Poales | Poaceae | Dactylis | glomerata | Grass | Likely Exotic | 2425 | 9 | 269 |
| Otu35 | trnL | Magnoliopsida | Fabales | Fabaceae | Acacia |  | Shrub or Tree | Likely Native | 3142 | 11 | 286 |
| Otu36 | trnL | Magnoliopsida | Poales | Cyperaceae | Carex |  | Grass-like Plants | Likely Native | 2855 | 2 | 1428 |
| Otu37 | trnL | Magnoliopsida | Poales | Poaceae | Rytidosperma |  | Grass | Likely Native | 2023 | 7 | 289 |
| Otu38 | trnL | Polypodiopsida | Cyatheales | Dicksoniaceae | Dicksonia | antarctica | Fern | Likely Native | 1479 | 14 | 106 |
| Otu39 | trnL | Magnoliopsida | Poales | Poaceae | Holcus | lanatus | Grass | Likely Exotic | 1149 | 9 | 128 |
| Otu40 | trnL | Polypodiopsida | Polypodiales | Blechnaceae | Blechnum |  | Fern | Likely Native | 1295 | 7 | 185 |
| Otu41 | trnL | Magnoliopsida | Proteales | Proteaceae | Persoonia |  | Shrub or Tree | Likely Native | 1345 | 7 | 192 |
| Otu42 | trnL | Magnoliopsida | Oxalidales | Cunoniaceae | Bauera | rubioides | Shrub or Tree | Likely Native | 1728 | 4 | 432 |
| Otu43 | trnL | Pinopsida | Pinales | Pinaceae |  |  | Shrub or Tree | Unknown | 1204 | 3 | 401 |
| Otu46 | trnL | Magnoliopsida | Caryophyllales | Polygonaceae | Persicaria |  | Forb | Likely Native | 977 | 4 | 244 |
| Otu47 | trnL | Magnoliopsida | Rosales | Urticaceae | Urtica |  | Forb | Likely Native | 923 | 8 | 115 |
| Otu48 | trnL | Pinopsida | Pinales | Pinaceae | Pinus | radiata | Shrub or Tree | Likely Exotic | 1044 | 5 | 209 |
| Otu49 | trnL | Magnoliopsida | Poales | Cyperaceae | Lepidosperma | tortuosum | Grass-like Plants | Likely Native | 874 | 7 | 125 |
| Otu50 | trnL | Magnoliopsida | Caryophyllales | Polygonaceae | Persicaria |  | Forb | Likely Native | 264 | 3 | 88 |
| Otu51 | trnL | Magnoliopsida | Myrtales | Onagraceae | Epilobium |  | Forb | Likely Native | 424 | 4 | 106 |
| Otu52 | trnL | Magnoliopsida | Poales | Poaceae |  |  | Grass | Unknown | 972 | 7 | 139 |
| Otu53 | trnL | Magnoliopsida | Sapindales | Rutaceae | Zieria |  | Shrub or Tree | Likely Native | 788 | 2 | 394 |
| Otu54 | trnL | Magnoliopsida | Fagales | Nothofagaceae | Nothofagus | cunninghamii | Shrub or Tree | Likely Native | 858 | 1 | 858 |
| Otu55 | trnL | Magnoliopsida | Sapindales | Rutaceae |  |  | Shrub or Tree | Unknown | 815 | 1 | 815 |
| Otu56 | trnL | Magnoliopsida | Fabales | Fabaceae | Glycine |  | Shrub or Tree | Likely Native | 606 | 3 | 202 |
| Otu58 | trnL | Magnoliopsida | Laurales | Lauraceae |  |  | Shrub or Tree | Unknown | 776 | 7 | 111 |
| Otu60 | trnL | Magnoliopsida | Poales | Poaceae | Phragmites | australis | Grass | Likely Native | 620 | 1 | 620 |
| Otu61 | trnL | Magnoliopsida | Asterales | Asteraceae | Cirsium |  | Forb | Likely Exotic | 410 | 5 | 82 |
| Otu62 | trnL | Magnoliopsida | Myrtales | Myrtaceae | Kunzea |  | Shrub or Tree | Likely Native | 501 | 3 | 167 |
| Otu63 | trnL | Magnoliopsida | Rosales | Rosaceae | Cotoneaster |  | Shrub or Tree | Likely Exotic | 293 | 3 | 98 |
| Otu65 | trnL | Polypodiopsida | Cyatheales | Dicksoniaceae | Calochlaena | dubia | Fern | Likely Native | 279 | 8 | 35 |
| Otu66 | trnL | Magnoliopsida | Ericales | Ericaceae |  |  | Shrub or Tree | Unknown | 449 | 3 | 150 |
| Otu67 | trnL | Magnoliopsida | Solanales | Solanaceae | Solanum | nigrum | Shrub or Tree | Likely Exotic | 144 | 2 | 72 |
| Otu68 | trnL | Magnoliopsida | Oxalidales | Oxalidaceae | Oxalis | corniculata | Forb | Likely Exotic | 407 | 6 | 68 |
| Otu69 | trnL | Magnoliopsida | Rosales | Rosaceae | Aphanes | arvensis | Forb | Likely Exotic | 10 | 1 | 10 |
| Otu70 | trnL | Magnoliopsida | Lamiales | Plantaginaceae | Plantago |  | Forb | Unknown | 48 | 2 | 24 |
| Otu75 | trnL | Magnoliopsida | Malpighiales | Salicaceae | Populus |  | Shrub or Tree | Likely Exotic | 408 | 3 | 136 |
| Otu78 | trnL | Magnoliopsida | Asterales | Goodeniaceae | Goodenia |  | Shrub or Tree | Likely Native | 349 | 4 | 87 |
| Otu83 | trnL | Magnoliopsida | Ranunculales | Ranunculaceae | Clematis |  | Forb | Likely Native | 287 | 4 | 72 |
| Otu88 | trnL | Magnoliopsida | Asterales | Campanulaceae | Wahlenbergia |  | Forb | Likely Native | 10 | 1 | 10 |
| Otu89 | trnL | Magnoliopsida | Poales | Cyperaceae | Lepidosperma |  | Grass-like Plants | Likely Native | 262 | 4 | 66 |
| Otu90 | trnL | Magnoliopsida | Ranunculales | Ranunculaceae | Ranunculus | repens | Forb | Likely Exotic | 238 | 2 | 119 |
| Otu91 | trnL | Magnoliopsida | Lamiales | Bignoniaceae | Pandorea | pandorana | Shrub or Tree | Likely Native | 275 | 3 | 92 |
| Otu93 | trnL | Polypodiopsida | Polypodiales | Dryopteridaceae | Polystichum | proliferum | Fern | Likely Native | 130 | 6 | 22 |
| Otu95 | trnL | Magnoliopsida | Gentianales | Rubiaceae |  |  | Forb | Unknown | 171 | 4 | 43 |
| Otu96 | trnL | Magnoliopsida | Ericales | Primulaceae |  |  | Forb | Unknown | 208 | 1 | 208 |
| Otu98 | trnL | Magnoliopsida | Myrtales | Lythraceae | Lythrum | salicaria | Forb | Unknown | 211 | 1 | 211 |
| Otu99 | trnL | Magnoliopsida | Laurales | Lauraceae | Cassytha |  | Shrub or Tree | Likely Native | 99 | 2 | 50 |
| Otu100 | trnL | Polypodiopsida | Polypodiales | Blechnaceae | Blechnum |  | Fern | Likely Native | 180 | 1 | 180 |
| Otu106 | trnL | Magnoliopsida | Gentianales | Rubiaceae |  |  | Forb | Unknown | 110 | 5 | 22 |
| Otu108 | trnL | Magnoliopsida | Malpighiales | Hypericaceae | Hypericum |  | Forb | Likely Exotic | 133 | 3 | 44 |
| Otu109 | trnL | Magnoliopsida | Malpighiales | Euphorbiaceae | Amperea | xiphoclada | Shrub or Tree | Likely Native | 142 | 1 | 142 |
| Otu110 | trnL | Magnoliopsida | Gentianales | Rubiaceae |  |  | Forb | Unknown | 143 | 3 | 48 |
| Otu112 | trnL | Magnoliopsida | Gentianales | Rubiaceae |  |  | Forb | Unknown | 135 | 3 | 45 |
| Otu114 | trnL | Magnoliopsida | Poales | Poaceae | Festuca |  | Grass | Likely Native | 125 | 4 | 31 |
| Otu117 | trnL | Magnoliopsida | Ericales | Ericaceae | Rhododendron |  | Shrub or Tree | Unknown | 121 | 1 | 121 |
| Otu118 | trnL | Magnoliopsida | Fabales | Fabaceae | Acacia |  | Shrub or Tree | Likely Native | 467 | 4 | 117 |
| Otu119 | trnL | Magnoliopsida | Fabales | Fabaceae | Acacia | melanoxyolon | Shrub or Tree | Likely Native | 141 | 3 | 47 |
| Otu120 | trnL | Magnoliopsida | Gentianales | Rubiaceae |  |  | Forb | Unknown | 15503 | 9 | 1723 |
| Otu121 | trnL | Magnoliopsida | Poales | Poaceae | Axonopus | fissifolius | Grass | Likely Exotic | 110 | 3 | 37 |
| Otu122 | trnL | Magnoliopsida | Apiales | Araliaceae | Polyscias | sambucifolia | Shrub or Tree | Likely Native | 90 | 2 | 45 |
| Otu123 | trnL | Magnoliopsida | Sapindales | Rutaceae | Correa |  | Shrub or Tree | Likely Native | 481 | 5 | 96 |
| Otu124 | trnL | Pinopsida | Pinales | Cupressaceae | Callitris |  | Shrub or Tree | Likely Native | 95 | 1 | 95 |
| Otu126 | trnL | Magnoliopsida | Santales | Loranthaceae | Muellerina | eucalyptoides | Shrub or Tree | Likely Native | 67 | 3 | 22 |
| Otu129 | trnL | Magnoliopsida | Gentianales | Rubiaceae |  |  | Forb | Unknown | 80 | 1 | 80 |
| Otu130 | trnL | Magnoliopsida | Gentianales | Rubiaceae |  |  | Forb | Unknown | 114 | 1 | 114 |
| Otu131 | trnL | Magnoliopsida | Lamiales | Plantaginaceae | Plantago |  | Forb | Unknown | 89 | 2 | 45 |
| Otu134 | trnL | Magnoliopsida | Gentianales | Rubiaceae |  |  | Forb | Unknown | 152 | 5 | 30 |
| Otu135 | trnL | Magnoliopsida | Poales | Cyperaceae | Gahnia | sieberiana | Grass-like Plants | Likely Native | 66 | 2 | 33 |
| Otu136 | trnL | Magnoliopsida | Asterales | Asteraceae |  |  | Shrub or Tree | Unknown | 111 | 2 | 56 |
| Otu137 | trnL | Polypodiopsida | Polypodiales | Pteridiaceae | Adiantum | aethiopicum | Creeper | Likely Native | 88 | 1 | 88 |
| Otu141 | trnL | Magnoliopsida | Lamiales | Plantaginaceae | Veronica |  | Forb | Likely Native | 56 | 2 | 28 |
| Otu142 | trnL | Magnoliopsida | Gentianales | Rubiaceae |  |  | Forb | Unknown | 85 | 1 | 85 |
| Otu143 | trnL | Magnoliopsida | Gentianales | Rubiaceae |  |  | Forb | Unknown | 144 | 2 | 72 |
| Otu147 | trnL | Magnoliopsida | Malpighiales | Hypericaceae | Hypericum | androsaemum | Forb | Likely Exotic | 64 | 2 | 32 |
| Otu150 | trnL | Magnoliopsida | Canellales | Winteraceae | Tasmannia |  | Shrub or Tree | Likely Native | 36 | 2 | 18 |
| Otu151 | trnL | Magnoliopsida | Gentianales | Rubiaceae |  |  | Forb | Unknown | 65 | 1 | 65 |
| Otu152 | trnL | Magnoliopsida | Poales | Poaceae | Bromus | catharticus | Grass | Likely Exotic | 65 | 1 | 65 |
| Otu153 | trnL | Magnoliopsida | Gentianales | Rubiaceae |  |  | Forb | Unknown | 64 | 1 | 64 |
| Otu156 | trnL | Magnoliopsida | Fabales | Fabaceae | Goodia | lotifolia | Shrub or Tree | Likely Native | 136 | 2 | 68 |
| Otu159 | trnL | Magnoliopsida | Ericales | Theaceae | Camelia |  | Shrub or Tree | Likely Exotic | 62 | 1 | 62 |
| Otu160 | trnL | Magnoliopsida | Poales | Poaceae | Eragrostis | brownii | Grass | Likely Native | 61 | 1 | 61 |
| Otu161 | trnL | Magnoliopsida | Gentianales | Rubiaceae |  |  | Forb | Unknown | 66 | 1 | 66 |
| Otu162 | trnL | Magnoliopsida | Gentianales | Rubiaceae |  |  | Forb | Unknown | 67 | 1 | 67 |
| Otu163 | trnL | Magnoliopsida | Gentianales | Rubiaceae |  |  | Forb | Unknown | 64 | 1 | 64 |
| Otu167 | trnL | Magnoliopsida | Gentianales | Apocynaceae |  |  | Shrub or Tree | Unknown | 42 | 1 | 42 |
| Otu168 | trnL | Magnoliopsida | Gentianales | Rubiaceae |  |  | Forb | Unknown | 67 | 1 | 67 |
| Otu176 | trnL | Magnoliopsida | Gentianales | Rubiaceae |  |  | Forb | Unknown | 231 | 2 | 116 |
| Otu177 | trnL | Magnoliopsida | Gentianales | Rubiaceae |  |  | Forb | Unknown | 49 | 2 | 25 |
| Otu178 | trnL | Magnoliopsida | Caryophyllales | Polygonaceae | Rumex |  | Forb | Likely Native | 57 | 3 | 19 |
| Otu181 | trnL | Magnoliopsida | Gentianales | Rubiaceae |  |  | Forb | Unknown | 97 | 3 | 32 |
| Otu184 | trnL | Magnoliopsida | Poales | Juncaceae | Juncus |  | Grass-like Plants | Likely Native | 56 | 1 | 56 |
| Otu185 | trnL | Magnoliopsida | Solanales | Convolvulaceae |  |  | Forb | Unknown | 49 | 3 | 16 |
| Otu186 | trnL | Magnoliopsida | Asterales | Asteraceae |  |  | Shrub or Tree | Unknown | 45 | 1 | 45 |
| Otu187 | trnL | Magnoliopsida | Myrtales | Myrtaceae | Leptospermum |  | Shrub or Tree | Likely Native | 153 | 2 | 77 |
| Otu193 | trnL | Magnoliopsida | Fabales | Fabaceae | Acacia |  | Shrub or Tree | Likely Native | 1122 | 10 | 112 |
| Otu195 | trnL | Magnoliopsida | Gentianales | Rubiaceae |  |  | Forb | Unknown | 51 | 1 | 51 |
| Otu196 | trnL | Magnoliopsida | Brassicales | Brassicaceae | Brassica | oleracea | Forb | Likely Exotic | 46 | 1 | 46 |
| Otu197 | trnL | Magnoliopsida | Asterales | Asteraceae | Helichrysum |  | Shrub or Tree | Likely Native | 34 | 1 | 34 |
| Otu198 | trnL | Magnoliopsida | Myrtales | Myrtaceae | Leptospermum |  | Shrub or Tree | Likely Native | 39 | 2 | 20 |
| Otu205 | trnL | Magnoliopsida | Myrtales | Myrtaceae |  |  | Shrub or Tree | Unknown | 27 | 2 | 14 |
| Otu210 | trnL | Magnoliopsida | Gentianales | Rubiaceae | Galium |  | Forb | Likely Native | 28 | 2 | 14 |
| Otu212 | trnL | Magnoliopsida | Gentianales | Rubiaceae |  |  | Forb | Unknown | 24 | 1 | 24 |
| Otu213 | trnL | Magnoliopsida | Gentianales | Rubiaceae |  |  | Forb | Unknown | 41 | 2 | 21 |
| Otu216 | trnL | Magnoliopsida | Fabales | Fabaceae | Indigofera | australis | Shrub or Tree | Likely Native | 41 | 1 | 41 |
| Otu217 | trnL | Magnoliopsida | Poales | Juncaceae | Juncus |  | Grass-like Plants | Likely Native | 41 | 1 | 41 |
| Otu218 | trnL | Magnoliopsida | Poales | Poaceae | Phleum | pratense | Grass | Likely Exotic | 216 | 2 | 108 |
| Otu222 | trnL | Magnoliopsida | Gentianales | Rubiaceae | Galium |  | Forb | Likely Native | 38 | 1 | 38 |
| Otu223 | trnL | Magnoliopsida | Apiales | Pittosporaceae |  |  | Shrub or Tree | Unknown | 33 | 2 | 17 |
| Otu235 | trnL | Magnoliopsida | Myrtales | Myrtaceae |  |  | Shrub or Tree | Unknown | 10 | 1 | 10 |
| Otu238 | trnL | Pinopsida | Pinales | Cupressaceae |  |  | Shrub or Tree | Unknown | 30 | 1 | 30 |
| Otu240 | trnL | Magnoliopsida | Myrtales | Myrtaceae |  |  | Shrub or Tree | Unknown | 32 | 1 | 32 |
| Otu241 | trnL | Polypodiopsida | Polypodiales | Dennstaedtiaceae | Histiopteris | incisa | Fern | Likely Native | 28 | 1 | 28 |
| Otu245 | trnL | Magnoliopsida | Poales | Poaceae |  |  | Grass | Unknown | 35 | 1 | 35 |
| Otu257 | trnL | Magnoliopsida | Malpighiales | Euphorbiaceae | Ricinus | communis | Shrub or Tree | Likely Exotic | 46 | 1 | 46 |
| Otu261 | trnL | Magnoliopsida | Gentianales | Rubiaceae | Galium |  | Forb | Likely Native | 33 | 1 | 33 |
| Otu269 | trnL | Magnoliopsida | Apiales | Pittosporaceae |  |  | Shrub or Tree | Unknown | 28 | 1 | 28 |
| Otu271 | trnL | Pinopsida | Pinales | Pinaceae |  |  | Shrub or Tree | Unknown | 32 | 1 | 32 |
| Otu272 | trnL | Magnoliopsida | Lamiales | Plantaginaceae | Veronica |  | Forb | Likely Native | 31 | 1 | 31 |
| Otu275 | trnL | Magnoliopsida | Gentianales | Rubiaceae |  |  | Forb | Unknown | 27 | 1 | 27 |
| Otu280 | trnL | Magnoliopsida | Gentianales | Rubiaceae |  |  | Forb | Unknown | 78 | 2 | 39 |
| Otu282 | trnL | Magnoliopsida | Gentianales | Rubiaceae | Galium |  | Forb | Likely Native | 53 | 2 | 27 |
| Otu285 | trnL | Magnoliopsida | Laurales | Lauraceae | Cassytha |  | Shrub or Tree | Likely Native | 39 | 1 | 39 |
| Otu288 | trnL | Magnoliopsida | Rosales | Rosaceae | Potentilla | recta | Shrub or Tree | Likely Exotic | 15 | 1 | 15 |
| Otu295 | trnL | Magnoliopsida | Gentianales | Rubiaceae |  |  | Forb | Unknown | 13 | 1 | 13 |
| Otu300 | trnL | Magnoliopsida | Poales | Poaceae |  |  | Grass | Unknown | 29 | 2 | 15 |
| Otu301 | trnL | Magnoliopsida | Fabales | Fabaceae |  |  | Shrub or Tree | Unknown | 15 | 1 | 15 |
| Otu312 | trnL | Magnoliopsida | Rosales | Rosaceae |  |  | Shrub or Tree | Unknown | 30 | 1 | 30 |
| Otu314 | trnL | Magnoliopsida | Gentianales | Rubiaceae | Galium |  | Forb | Likely Native | 23 | 1 | 23 |
| Otu315 | trnL | Magnoliopsida | Poales | Poaceae |  |  | Grass | Unknown | 11 | 1 | 11 |
| Otu318 | trnL | Magnoliopsida | Gentianales | Rubiaceae | Galium |  | Forb | Likely Native | 22 | 1 | 22 |
| Otu320 | trnL | Magnoliopsida | Poales | Cyperaceae | Lepidosperma |  | Grass-like Plants | Likely Native | 15 | 1 | 15 |
| Otu330 | trnL | Magnoliopsida | Fabales | Fabaceae | Acacia |  | Shrub or Tree | Likely Native | 331 | 4 | 83 |
| Otu331 | trnL | Magnoliopsida | Gentianales | Rubiaceae |  |  | Forb | Unknown | 10 | 1 | 10 |
| Otu335 | trnL | Magnoliopsida | Asterales | Asteraceae |  |  | Shrub or Tree | Unknown | 14 | 1 | 14 |
| Otu339 | trnL | Magnoliopsida | Gentianales | Rubiaceae |  |  | Forb | Unknown | 20 | 1 | 20 |
| Otu341 | trnL | Magnoliopsida | Gentianales | Rubiaceae |  |  | Forb | Unknown | 10 | 1 | 10 |
| Otu343 | trnL | Magnoliopsida | Gentianales | Rubiaceae |  |  | Forb | Unknown | 24 | 1 | 24 |
| Otu346 | trnL | Magnoliopsida | Gentianales | Rubiaceae |  |  | Forb | Unknown | 18 | 1 | 18 |
| Otu347 | trnL | Magnoliopsida | Gentianales | Rubiaceae |  |  | Forb | Unknown | 24 | 1 | 24 |
| Otu348 | trnL | Magnoliopsida | Apiales | Apiaceae | Centella | cordifolia | Forb | Likely Native | 17 | 1 | 17 |
| Otu351 | trnL | Magnoliopsida | Fabales | Fabaceae |  |  | Shrub or Tree | Unknown | 33 | 1 | 33 |
| Otu358 | trnL | Pinopsida | Pinales | Cuppressaceae |  |  | Shrub or Tree | Unknown | 20 | 1 | 20 |
| Otu394 | trnL | Pinopsida | Pinales | Podocarpaceae | Podocarpus | lawrencei | Shrub or Tree | Likely Native | 15 | 1 | 15 |
| Otu396 | trnL | Magnoliopsida | Gentianales | Rubiaceae | Galium |  | Forb | Likely Native | 17 | 1 | 17 |
| Otu403 | trnL | Magnoliopsida | Poales | Cyperaceae | Gahnia |  | Grass-like Plants | Likely Native | 15 | 1 | 15 |
| Otu409 | trnL | Magnoliopsida | Rosales | Rosaceae |  |  | Shrub or Tree | Unknown | 10 | 1 | 10 |
| Otu411 | trnL | Polypodiopsida | Osmundales | Osmundaceae | Todea | barbara | Fern | Likely Native | 13 | 1 | 13 |
| Otu416 | trnL | Magnoliopsida | Asparagales | Iridaceae | Sisyrinchium | micranthum | Forb | Likely Exotic | 11 | 1 | 11 |
| Otu418 | trnL | Magnoliopsida | Poales | Poaceae | Agrostis |  | Grass | Likely Native | 22 | 1 | 22 |
| Otu424 | trnL | Magnoliopsida | Gentianales | Rubiaceae |  |  | Forb | Unknown | 10 | 1 | 10 |
| Otu426 | trnL | Magnoliopsida | Asterales | Asteraceae | Helichrysum |  | Shrub or Tree | Likely Native | 11 | 1 | 11 |
| Otu427 | trnL | Magnoliopsida | Canellales | Winteraceae | Tasmannia |  | Shrub or Tree | Likely Native | 11 | 1 | 11 |
| Otu428 | trnL | Magnoliopsida | Gentianales | Rubiaceae | Galium |  | Forb | Likely Native | 13 | 1 | 13 |
| Otu436 | trnL | Magnoliopsida | Gentianales | Rubiaceae | Galium |  | Forb | Likely Native | 16 | 1 | 16 |
| Otu438 | trnL | Magnoliopsida | Gentianales | Rubiaceae | Galium |  | Forb | Likely Native | 13 | 1 | 13 |
| Otu442 | trnL | Magnoliopsida | Myrtales | Myrtaceae |  |  | Shrub or Tree | Unknown | 11 | 1 | 11 |
| Otu448 | trnL | Magnoliopsida | Asterales | Asteraceae |  |  | Shrub or Tree | Unknown | 1678 | 11 | 153 |
| Otu451 | trnL | Magnoliopsida | Fabales | Fabaceae | Acacia |  | Shrub or Tree | Likely Native | 10 | 1 | 10 |
| Otu456 | trnL | Magnoliopsida | Rosales | Rhamnaceae |  |  | Shrub or Tree | Unknown | 11 | 1 | 11 |
| Otu466 | trnL | Magnoliopsida | Poales | Poaceae | Poa |  | Grass | Likely Native | 10 | 1 | 10 |
| Otu484 | trnL | Magnoliopsida | Lamiales | Lamiaceae | Prostanthera |  | Shrub or Tree | Likely Native | 10 | 1 | 10 |
| Otu504 | trnL | Magnoliopsida | Gentianales | Rubiaceae | Galium |  | Forb | Likely Native | 17 | 1 | 17 |
| Otu509 | trnL | Magnoliopsida | Gentianales | Rubiaceae |  |  | Forb | Unknown | 13 | 1 | 13 |
| Otu516 | trnL | Magnoliopsida | Gentianales | Rubiaceae |  |  | Forb | Unknown | 16 | 1 | 16 |
| Otu528 | trnL | Magnoliopsida | Gentianales | Rubiaceae |  |  | Forb | Unknown | 10 | 1 | 10 |
| Otu533 | trnL | Magnoliopsida | Fabales | Fabaceae | Acacia |  | Shrub or Tree | Likely Native | 126 | 1 | 126 |
| Otu549 | trnL | Magnoliopsida | Fagales | Fagaceae | Quercus | robur | Shrub or Tree | Likely Exotic | 13 | 1 | 13 |
| Otu558 | trnL | Magnoliopsida | Gentianales | Rubiaceae | Galium |  | Forb | Likely Native | 10 | 1 | 10 |
| Otu559 | trnL | Magnoliopsida | Myrtales | Myrtaceae |  |  | Shrub or Tree | Unknown | 14 | 1 | 14 |
| Otu575 | trnL | Magnoliopsida | Gentianales | Rubiaceae |  |  | Forb | Unknown | 32 | 2 | 16 |
| Otu577 | trnL | Magnoliopsida | Poales | Poaceae |  |  | Grass | Unknown | 10 | 1 | 10 |
| Otu586 | trnL | Magnoliopsida | Poales | Poaceae | Dichelachne | crinita | Grass | Likely Native | 1054 | 7 | 151 |
| Otu588 | trnL | Magnoliopsida | Gentianales | Rubiaceae |  |  | Forb | Unknown | 357 | 4 | 89 |
| Otu598 | trnL | Magnoliopsida | Asterales | Asteraceae | Erigeron |  | Forb | Likely Exotic | 352 | 8 | 44 |
| Otu602 | trnL | Magnoliopsida | Myrtales | Myrtaceae |  |  | Shrub or Tree | Unknown | 11 | 1 | 11 |
| Otu627 | trnL | Magnoliopsida | Gentianales | Rubiaceae |  |  | Forb | Unknown | 10 | 1 | 10 |
| Otu718 | trnL | Magnoliopsida | Oxalidales | Cunoniaceae | Bauera | rubioides | Shrub or Tree | Likely Native | 10 | 1 | 10 |
| Otu819 | trnL | Magnoliopsida | Canellales | Winteraceae | Tasmannia |  | Shrub or Tree | Likely Native | 488 | 7 | 70 |
| Otu825 | trnL | Magnoliopsida | Poales | Poaceae |  |  | Grass | Unknown | 10 | 1 | 10 |
| Otu859 | trnL | Magnoliopsida | Asterales | Asteraceae | Helichrysum |  | Shrub or Tree | Likely Native | 224 | 2 | 112 |
| Otu891 | trnL | Magnoliopsida | Ranunculales | Ranunculaceae | Clematis |  | Forb | Likely Native | 120 | 3 | 40 |
| Otu893 | trnL | Magnoliopsida | Apiales | Araliaceae | Hydrocotyle |  | Forb | Likely Native | 88 | 3 | 29 |
| Otu904 | trnL | Magnoliopsida | Poales | Poaceae |  |  | Grass | Unknown | 10 | 1 | 10 |
| Otu1072 | trnL | Magnoliopsida | Rosales | Rosaceae |  |  | Shrub or Tree | Unknown | 130 | 4 | 33 |
| Otu1118 | trnL | Magnoliopsida | Rosales | Rhamnaceae | Spyridium |  | Shrub or Tree | Likely Native | 433 | 2 | 217 |
| Otu1123 | trnL | Magnoliopsida | Rosales | Rosaceae |  |  | Shrub or Tree | Unknown | 175 | 6 | 29 |
| Otu1143 | trnL | Magnoliopsida | Poales | Juncaceae | Juncus |  | Grass-like Plants | Likely Native | 81 | 1 | 81 |
| Otu1173 | trnL | Magnoliopsida | Laurales | Lauraceae |  |  | Shrub or Tree | Unknown | 234 | 2 | 117 |
| Otu1181 | trnL | Magnoliopsida | Myrtales | Myrtaceae |  |  | Shrub or Tree | Unknown | 11 | 1 | 11 |
| Otu1231 | trnL | Polypodiopsida | Polypodiales | Blechnaceae | Blechnum |  | Fern | Likely Native | 89 | 3 | 30 |
| Otu1253 | trnL | Magnoliopsida | Myrtales | Myrtaceae |  |  | Shrub or Tree | Unknown | 41 | 2 | 21 |
| Otu1313 | trnL | Magnoliopsida | Fabales | Fabaceae |  |  | Shrub or Tree | Unknown | 321 | 3 | 107 |
| Otu1351 | trnL | Magnoliopsida | Fabales | Fabaceae |  |  | Shrub or Tree | Unknown | 243 | 3 | 81 |
| Otu1360 | trnL | Magnoliopsida | Ranunculales | Ranunculaceae | Clematis |  | Forb | Likely Native | 50 | 3 | 17 |
| Otu1375 | trnL | Magnoliopsida | Rosales | Rosaceae |  |  | Shrub or Tree | Unknown | 155 | 6 | 26 |
| Otu1380 | trnL | Magnoliopsida | Poales | Poaceae |  |  | Grass | Unknown | 25 | 1 | 25 |
| Otu1433 | trnL | Magnoliopsida | Rosales | Rosaceae |  |  | Shrub or Tree | Unknown | 211 | 7 | 30 |
| Otu1785 | trnL | Magnoliopsida | Myrtales | Myrtaceae |  |  | Shrub or Tree | Unknown | 68 | 2 | 34 |
| Otu1898 | trnL | Magnoliopsida | Poales | Juncaceae | Juncus |  | Grass-like Plants | Likely Native | 26 | 1 | 26 |
| Otu2411 | trnL | Magnoliopsida | Rosales | Rosaceae |  |  | Shrub or Tree | Unknown | 29 | 1 | 29 |
| Otu2465 | trnL | Magnoliopsida | Fabales | Fabaceae | Acacia |  | Shrub or Tree | Likely Native | 22 | 1 | 22 |
| Otu5 | ITS2 | Magnoliopsida | Rosales | Rosaceae | Rubus |  | Shrub or Tree | Likely Exotic | 83788 | 18 | 4655 |
| Otu6 | ITS2 | Magnoliopsida | Canellales | Winteraceae | Tasmannia |  | Shrub or Tree | Likely Native | 31666 | 9 | 3518 |
| Otu7 | ITS2 | Magnoliopsida | Saxifragales | Haloragaceae | Gonocarpus |  | Forb | Likely Native | 55548 | 14 | 3968 |
| Otu10 | ITS2 | Magnoliopsida | Gentianales | Rubiaceae | Coprosma |  | Shrub or Tree | Likely Native | 32354 | 13 | 2489 |
| Otu11 | ITS2 | Magnoliopsida | Rosales | Rhamnaceae | Pomaderris |  | Shrub or Tree | Likely Native | 15848 | 9 | 1761 |
| Otu15 | ITS2 | Magnoliopsida | Rosales | Rosaceae | Acaena | novae-zelandiae | Forb | Likely Native | 21031 | 17 | 1237 |
| Otu16 | ITS2 | Magnoliopsida | Poales | Poaceae | Microlaena |  | Grass | Likely Native | 9044 | 16 | 565 |
| Otu18 | ITS2 | Magnoliopsida | Gentianales | Rubiaceae | Leptostigma | breviflorum | Forb | Likely Native | 4177 | 3 | 1392 |
| Otu19 | ITS2 | Magnoliopsida | Lamiales | Plantaginaceae | Plantago | lanceolata | Forb | Likely Exotic | 4305 | 13 | 331 |
| Otu22 | ITS2 | Magnoliopsida | Lamiales | Lamiaceae |  |  | Shrub or Tree | Unknown | 4187 | 11 | 381 |
| Otu30 | ITS2 | Magnoliopsida | Fabales | Fabaceae | Pultenaea | muelleri | Shrub or Tree | Likely Native | 4829 | 7 | 690 |
| Otu31 | ITS2 | Magnoliopsida | Apiales | Araliaceae | Hydrocotyle |  | Forb | Likely Native | 2363 | 3 | 788 |
| Otu32 | ITS2 | Magnoliopsida | Myrtales | Myrtaceae | Eucalyptus |  | Shrub or Tree | Likely Native | 3743 | 14 | 267 |
| Otu34 | ITS2 | Magnoliopsida | Myrtales | Myrtaceae | Eucalyptus |  | Shrub or Tree | Likely Native | 3874 | 13 | 298 |
| Otu38 | ITS2 | Magnoliopsida | Poales | Poaceae | Poa |  | Grass | Likely Native | 1877 | 9 | 209 |
| Otu40 | ITS2 | Magnoliopsida | Gentianales | Rubiaceae | Galium |  | Forb | Likely Native | 4816 | 9 | 535 |
| Otu41 | ITS2 | Magnoliopsida | Asterales | Asteraceae | Cassinia | aculeata | Shrub or Tree | Likely Native | 2739 | 9 | 304 |
| Otu42 | ITS2 | Magnoliopsida | Fagales | Fagaceae | Quercus | robur | Shrub or Tree | Likely Exotic | 1363 | 1 | 1363 |
| Otu43 | ITS2 | Magnoliopsida | Asterales | Asteraceae | Hypochaeris | radicata | Forb | Likely Exotic | 626 | 9 | 70 |
| Otu46 | ITS2 | Magnoliopsida | Myrtales | Myrtaceae | Leptospermum | scoparium | Shrub or Tree | Likely Native | 2643 | 4 | 661 |
| Otu47 | ITS2 | Magnoliopsida | Oxalidales | Elaeocarpaceae | Tetratheca | cilata | Shrub or Tree | Likely Native | 1500 | 4 | 375 |
| Otu50 | ITS2 | Magnoliopsida | Myrtales | Myrtaceae | Eucalyptus | regnans | Shrub or Tree | Likely Native | 2165 | 8 | 271 |
| Otu51 | ITS2 | Magnoliopsida | Myrtales | Onagraceae | Epilobium |  | Forb | Likely Native | 1033 | 9 | 115 |
| Otu55 | ITS2 | Magnoliopsida | Apiales | Araliaceae | Hydrocotyle |  | Forb | Likely Native | 21 | 2 | 11 |
| Otu56 | ITS2 | Magnoliopsida | Rosales | Rosaceae | Aphanes | arvensis | Forb | Likely Exotic | 14 | 1 | 14 |
| Otu58 | ITS2 | Magnoliopsida | Fagales | Nothofagaceae | Nothofagus | cunninghamii | Shrub or Tree | Likely Native | 1322 | 2 | 661 |
| Otu59 | ITS2 | Magnoliopsida | Fabales | Fabaceae | Trifolium | repens | Forb | Likely Exotic | 1969 | 3 | 656 |
| Otu66 | ITS2 | Magnoliopsida | Myrtales | Lythraceae | Lythrum | hyssopifolia | Forb | Unknown | 765 | 1 | 765 |
| Otu69 | ITS2 | Magnoliopsida | Caryophyllales | Caryophyllaceae | Cerastium |  | Forb | Likely Exotic | 12 | 1 | 12 |
| Otu70 | ITS2 | Magnoliopsida | Rosales | Rosaceae | Rubus |  | Shrub or Tree | Likely Exotic | 1502 | 9 | 167 |
| Otu73 | ITS2 | Magnoliopsida | Poales | Cyperaceae | Isolepis | inundata | Grass-like Plants | Likely Native | 620 | 3 | 207 |
| Otu76 | ITS2 | Magnoliopsida | Sapindales | Rutaceae | Correa | lawrenceana | Shrub or Tree | Likely Native | 833 | 2 | 417 |
| Otu79 | ITS2 | Magnoliopsida | Fabales | Fabaceae | Lotus | uliginosus | Forb | Likely Exotic | 1273 | 11 | 116 |
| Otu80 | ITS2 | Magnoliopsida | Fabales | Fabaceae | Platylobium |  | Shrub or Tree | Likely Native | 669 | 1 | 669 |
| Otu81 | ITS2 | Magnoliopsida | Lamiales | Plantaginaceae | Plantago | major | Forb | Likely Exotic | 33 | 1 | 33 |
| Otu82 | ITS2 | Magnoliopsida | Sapindales | Rutaceae | Zieria | arborescens | Shrub or Tree | Likely Native | 580 | 2 | 290 |
| Otu86 | ITS2 | Magnoliopsida | Poales | Poaceae | Holcus | lanatus | Grass | Likely Exotic | 42 | 4 | 11 |
| Otu87 | ITS2 | Magnoliopsida | Rosales | Urticaceae | Urtica | incisa | Forb | Likely Native | 47 | 1 | 47 |
| Otu93 | ITS2 | Magnoliopsida | Poales | Poaceae | Anthoxanthum | aristatum | Grass | Likely Exotic | 565 | 13 | 43 |
| Otu94 | ITS2 | Magnoliopsida | Poales | Juncaceae | Juncus | bulbosus | Grass-like Plants | Likely Exotic | 358 | 2 | 179 |
| Otu98 | ITS2 | Magnoliopsida | Asparagales | Amaryllidaceae | Allium | triquetrum | Forb | Likely Exotic | 646 | 1 | 646 |
| Otu101 | ITS2 | Magnoliopsida | Poales | Poaceae | Microlaena | stipoides | Grass | Likely Native | 699 | 13 | 54 |
| Otu102 | ITS2 | Magnoliopsida | Asterales | Asteraceae | Sonchus | oleraceus | Forb | Likely Exotic | 5 | 1 | 5 |
| Otu105 | ITS2 | Magnoliopsida | Myrtales | Myrtaceae | Leptospermum | lanigerum | Shrub or Tree | Likely Native | 455 | 5 | 91 |
| Otu107 | ITS2 | Magnoliopsida | Gentianales | Rubiaceae | Coprosma | hirtella | Shrub or Tree | Likely Native | 2221 | 4 | 555 |
| Otu111 | ITS2 | Magnoliopsida | Caryophyllales | Polygonaceae | Acetosella | vulgaris | Forb | Likely Exotic | 1530 | 6 | 255 |
| Otu114 | ITS2 | Magnoliopsida | Lamiales | Plantaginaceae | Callitriche | stagnalis | Forb | Likely Exotic | 174 | 1 | 174 |
| Otu117 | ITS2 | Magnoliopsida | Cyatheales | Cyatheaceae | Cyathea |  | Fern | Likely Native | 519 | 5 | 104 |
| Otu118 | ITS2 | Magnoliopsida | Malpighiales | Salicaceae | Populus |  | Shrub or Tree | Likely Exotic | 239 | 2 | 120 |
| Otu125 | ITS2 | Magnoliopsida | Laurales | Monimiaceae | Hedycarya | angustifolia | Shrub or Tree | Likely Native | 136 | 3 | 45 |
| Otu126 | ITS2 | Magnoliopsida | Geraniales | Geraniaceae | Geranium |  | Forb | Likely Native | 349 | 9 | 39 |
| Otu131 | ITS2 | Magnoliopsida | Fagales | Fagaceae | Quercus |  | Shrub or Tree | Likely Exotic | 226 | 1 | 226 |
| Otu132 | ITS2 | Magnoliopsida | Canellales | Winteraceae | Tasmannia |  | Shrub or Tree | Likely Native | 128 | 3 | 43 |
| Otu135 | ITS2 | Magnoliopsida | Myrtales | Myrtaceae | Kunzea |  | Shrub or Tree | Likely Native | 117 | 2 | 59 |
| Otu136 | ITS2 | Magnoliopsida | Caryophyllales | Polygonaceae | Persicaria |  | Forb | Likely Native | 123 | 1 | 123 |
| Otu138 | ITS2 | Magnoliopsida | Ericales | Ericaceae | Epacris |  | Shrub or Tree | Likely Native | 110 | 2 | 55 |
| Otu142 | ITS2 | Magnoliopsida | Caryophyllales | Caryophyllaceae | Stellaria |  | Forb | Likely Native | 135 | 5 | 27 |
| Otu144 | ITS2 | Magnoliopsida | Proteales | Proteaceae | Lomatia |  | Shrub or Tree | Likely Native | 197 | 5 | 39 |
| Otu146 | ITS2 | Magnoliopsida | Asterales | Asteraceae | Argyrotegium | fordianum | Forb | Likely Native | 124 | 2 | 62 |
| Otu150 | ITS2 | Magnoliopsida | Poales | Poaceae | Agrostis | capillaris | Grass | Likely Exotic | 188 | 9 | 21 |
| Otu152 | ITS2 | Magnoliopsida | Fabales | Fabaceae | Acacia | verticillata | Shrub or Tree | Likely Native | 83 | 4 | 21 |
| Otu153 | ITS2 | Magnoliopsida | Poales | Poaceae | Anthoxanthum | odoratum | Grass | Likely Exotic | 467 | 14 | 33 |
| Otu156 | ITS2 | Magnoliopsida | Poales | Cyperaceae | Lepidosperma | laterale | Grass-like Plants | Likely Native | 159 | 4 | 40 |
| Otu159 | ITS2 | Magnoliopsida | Fabales | Fabaceae | Glycine | microphylla | Climber | Likely Native | 101 | 3 | 34 |
| Otu161 | ITS2 | Magnoliopsida | Fagales | Fagaceae | Quercus |  | Shrub or Tree | Likely Exotic | 793 | 1 | 793 |
| Otu162 | ITS2 | Magnoliopsida | Gentianales | Rubiaceae |  |  | Forb | Unknown | 57 | 1 | 57 |
| Otu165 | ITS2 | Magnoliopsida | Asterales | Asteraceae | Olearia |  | Forb | Likely Native | 116 | 4 | 29 |
| Otu166 | ITS2 | Magnoliopsida | Asterales | Asteraceae | Cirsium | vulgare | Forb | Likely Exotic | 103 | 4 | 26 |
| Otu167 | ITS2 | Magnoliopsida | Santales | Loranthaceae | Muellerina | eucalyptoides | Shrub or Tree | Likely Native | 83 | 2 | 42 |
| Otu171 | ITS2 | Magnoliopsida | Gentianales | Rubiaceae |  |  | Forb | Unknown | 46 | 1 | 46 |
| Otu172 | ITS2 | Magnoliopsida | Ericales | Ericaceae | Leucopogon |  | Shrub or Tree | Likely Native | 112 | 1 | 112 |
| Otu173 | ITS2 | Magnoliopsida | Gentianales | Rubiaceae | Opercularia | varia | Forb | Likely Native | 78 | 1 | 78 |
| Otu174 | ITS2 | Magnoliopsida | Ericales | Primulaceae | Myrsine | howittiana | Shrub or Tree | Likely Native | 64 | 1 | 64 |
| Otu178 | ITS2 | Magnoliopsida | Canellales | Winteraceae | Tasmannia |  | Shrub or Tree | Likely Native | 59 | 2 | 30 |
| Otu183 | ITS2 | Magnoliopsida | Asterales | Asteraceae | Senecio |  | Forb | Likely Native | 209 | 5 | 42 |
| Otu187 | ITS2 | Magnoliopsida | Poales | Poaceae | Dactylis | glomerata | Grass | Likely Exotic | 67 | 3 | 22 |
| Otu191 | ITS2 | Magnoliopsida | Canellales | Winteraceae | Tasmannia |  | Shrub or Tree | Likely Native | 56 | 3 | 19 |
| Otu192 | ITS2 | Magnoliopsida | Malpighiales | Euphorbiaceae |  |  | Shrub or Tree | Unknown | 40 | 1 | 40 |
| Otu198 | ITS2 | Magnoliopsida | Fagales | Fagaceae | Quercus | robur | Shrub or Tree | Likely Exotic | 31 | 1 | 31 |
| Otu206 | ITS2 | Magnoliopsida | Lamiales | Lamiaceae | Prunella | vulgaris | Forb | Likely Exotic | 55 | 3 | 18 |
| Otu210 | ITS2 | Magnoliopsida | Oxalidales | Oxalidaceae | Oxalis | corniculata | Forb | Likely Exotic | 46 | 1 | 46 |
| Otu212 | ITS2 | Magnoliopsida | Apiales | Pittospiraceae | Billardiera | macrantha | Shrub or Tree | Likely Native | 80 | 2 | 40 |
| Otu213 | ITS2 | Magnoliopsida | Fabales | Fabaceae | Acacia |  | Shrub or Tree | Likely Native | 31 | 1 | 31 |
| Otu220 | ITS2 | Magnoliopsida | Fabales | Fabaceae | Platylobium |  | Shrub or Tree | Likely Native | 44 | 1 | 44 |
| Otu221 | ITS2 | Magnoliopsida | Rosales | Rhamnaceae | Spyridium | parvifolium | Shrub or Tree | Likely Native | 54 | 2 | 27 |
| Otu222 | ITS2 | Magnoliopsida | Ranunculales | Ranunculaceae | Ranunculus | repens | Forb | Likely Exotic | 34 | 2 | 17 |
| Otu225 | ITS2 | Magnoliopsida | Rosales | Rosaceae | Rubus |  | Shrub or Tree | Likely Exotic | 32 | 3 | 11 |
| Otu227 | ITS2 | Magnoliopsida | Gentianales | Rubiaceae |  |  | Forb | Unknown | 20 | 1 | 20 |
| Otu236 | ITS2 | Magnoliopsida | Poales | Poaceae | Dryopoa | dives | Grass | Likely Native | 35 | 2 | 18 |
| Otu237 | ITS2 | Magnoliopsida | Gentianales | Rubiaceae | Coprosma |  | Shrub or Tree | Likely Native | 19 | 1 | 19 |
| Otu239 | ITS2 | Magnoliopsida | Fagales | Fagaceae | Quercus |  | Shrub or Tree | Likely Exotic | 35 | 1 | 35 |
| Otu246 | ITS2 | Magnoliopsida | Canellales | Winteraceae | Tasmannia |  | Shrub or Tree | Likely Native | 37 | 3 | 12 |
| Otu248 | ITS2 | Magnoliopsida | Rosales | Rosaceae | Rubus |  | Shrub or Tree | Likely Exotic | 29 | 2 | 15 |
| Otu251 | ITS2 | Magnoliopsida | Apiales | Apiaceae | Oreomyrrhis |  | Forb | Likely Native | 21 | 2 | 11 |
| Otu252 | ITS2 | Magnoliopsida | Gentianales | Rubiaceae |  |  | Forb | Unknown | 9 | 1 | 9 |
| Otu255 | ITS2 | Magnoliopsida | Gentianales | Rubiaceae |  |  | Forb | Unknown | 25 | 1 | 25 |
| Otu256 | ITS2 | Magnoliopsida | Asterales | Asteraceae | Leontodon | saxatilis | Forb | Likely Exotic | 48 | 4 | 12 |
| Otu257 | ITS2 | Magnoliopsida | Rosales | Rosaceae | Malus | pumila | Shrub or Tree | Likely Exotic | 39 | 1 | 39 |
| Otu258 | ITS2 | Magnoliopsida | Asparagales | Asphodelaceae | Dianella | tasmanica | Grass-like Plants | Likely Native | 48 | 1 | 48 |
| Otu259 | ITS2 | Magnoliopsida | Rosales | Rhamnaceae | Pomaderris |  | Shrub or Tree | Likely Native | 193 | 4 | 48 |
| Otu260 | ITS2 | Magnoliopsida | Gentianales | Rubiaceae |  |  | Forb | Unknown | 13 | 1 | 13 |
| Otu261 | ITS2 | Magnoliopsida | Rosales | Rhamnaceae | Pomaderris |  | Shrub or Tree | Likely Native | 64 | 1 | 64 |
| Otu262 | ITS2 | Magnoliopsida | Gentianales | Rubiaceae | Coprosma |  | Shrub or Tree | Likely Native | 24 | 3 | 8 |
| Otu263 | ITS2 | Magnoliopsida | Lamiales | Lamiaceae |  |  | Forb | Unknown | 60 | 3 | 20 |
| Otu265 | ITS2 | Magnoliopsida | Rosales | Rosaceae | Eriobotrya | japonica | Shrub or Tree | Likely Exotic | 20 | 1 | 20 |
| Otu266 | ITS2 | Magnoliopsida | Malvales | Thymelaeaeae | Pimelea | axiflora | Shrub or Tree | Likely Native | 19 | 1 | 19 |
| Otu267 | ITS2 | Magnoliopsida | Poales | Cyperaceae | Carex |  | Grass-like Plants | Likely Native | 62 | 2 | 31 |
| Otu279 | ITS2 | Magnoliopsida | Poales | Cyperaceae | Cyperus | eragrostis | Grass-like Plants | Likely Exotic | 17 | 1 | 17 |
| Otu281 | ITS2 | Magnoliopsida | Asterales | Asteraceae | Gamochaeta | americana | Forb | Likely Exotic | 14 | 2 | 7 |
| Otu286 | ITS2 | Magnoliopsida | Malpighiales | Hypericaceae | Hypericum | japonicum | Forb | Likely Native | 18 | 2 | 9 |
| Otu293 | ITS2 | Magnoliopsida | Apiales | Araliaceae | Polyscias | sambucifolia | Shrub or Tree | Likely Native | 13 | 1 | 13 |
| Otu299 | ITS2 | Magnoliopsida | Poales | Poaceae | Bromus | hordeaceus | Grass | Likely Exotic | 11 | 1 | 11 |
| Otu301 | ITS2 | Magnoliopsida | Ranunculales | Ranunculaceae | Clematis |  | Climber | Likely Native | 13 | 1 | 13 |
| Otu302 | ITS2 | Magnoliopsida | Saxifragales | Haloragaceae | Gonocarpus |  | Forb | Likely Native | 28 | 1 | 28 |
| Otu306 | ITS2 | Magnoliopsida | Gentianales | Rubiaceae | Coprosma |  | Shrub or Tree | Likely Native | 35 | 2 | 18 |
| Otu307 | ITS2 | Magnoliopsida | Gentianales | Rubiaceae | Coprosma |  | Shrub or Tree | Likely Native | 14 | 2 | 7 |
| Otu312 | ITS2 | Magnoliopsida | Gentianales | Rubiaceae |  |  | Forb | Unknown | 9 | 1 | 9 |
| Otu316 | ITS2 | Jungermanniopsida | Metzgeriales | Metzgeriaceae | Metzgeria | furcata | Liverwort | Unknown | 16 | 1 | 16 |
| Otu317 | ITS2 | Magnoliopsida | Sapindales | Rutaceae | Correa | reflexa | Shrub or Tree | Likely Native | 46 | 2 | 23 |
| Otu318 | ITS2 | Magnoliopsida | Malpighiales | Hypericaceae | Hypericum |  | Forb | Likely Exotic | 23 | 1 | 23 |
| Otu319 | ITS2 | Magnoliopsida | Gentianales | Rubiaceae |  |  | Forb | Unknown | 12 | 1 | 12 |
| Otu320 | ITS2 | Magnoliopsida | Asterales | Asteraceae | Helianthus | annuus | Forb | Likely Exotic | 20 | 1 | 20 |
| Otu322 | ITS2 | Magnoliopsida | Poales | Poaceae | Dichelachne |  | Grass | Likely Native | 17 | 1 | 17 |
| Otu323 | ITS2 | Magnoliopsida | Canellales | Winteraceae | Tasmannia |  | Shrub or Tree | Likely Native | 8 | 1 | 8 |
| Otu325 | ITS2 | Magnoliopsida | Poales | Poaceae | Setaria |  | Grass | Likely Exotic | 20 | 2 | 10 |
| Otu327 | ITS2 | Magnoliopsida | Canellales | Winteraceae | Tasmannia |  | Shrub or Tree | Likely Native | 15 | 1 | 15 |
| Otu330 | ITS2 | Magnoliopsida | Lamiales | Plantaginaceae | Gratiola |  | Forb | Likely Native | 37 | 1 | 37 |
| Otu331 | ITS2 | Magnoliopsida | Poales | Poaceae | Lolium | multiflorum | Grass | Likely Exotic | 31 | 2 | 16 |
| Otu332 | ITS2 | Magnoliopsida | Rosales | Rosaceae | Malus | pumila | Shrub or Tree | Likely Exotic | 103 | 2 | 52 |
| Otu333 | ITS2 | Magnoliopsida | Fabales | Fabaceae | Lotus | subbiflorus | Forb | Likely Exotic | 19 | 1 | 19 |
| Otu334 | ITS2 | Magnoliopsida | Gentianales | Rubiaceae | Coprosma |  | Shrub or Tree | Likely Native | 13 | 1 | 13 |
| Otu338 | ITS2 | Magnoliopsida | Asterales | Asteraceae | Olearia | argophylla | Forb | Likely Native | 24 | 1 | 24 |
| Otu340 | ITS2 | Magnoliopsida | Caryophyllales | Caryophyllaceae | Stellaria |  | Forb | Likely Native | 27 | 1 | 27 |
| Otu345 | ITS2 | Magnoliopsida | Poales | Poaceae | Glyceria |  | Grass | Likely Exotic | 6 | 1 | 6 |
| Otu353 | ITS2 | Magnoliopsida | Rosales | Rhamnaceae | Pomaderris |  | Shrub or Tree | Likely Native | 124 | 1 | 124 |
| Otu354 | ITS2 | Magnoliopsida | Gentianales | Rubiaceae |  |  | Forb | Unknown | 10 | 1 | 10 |
| Otu357 | ITS2 | Magnoliopsida | Gentianales | Rubiaceae | Coprosma |  | Shrub or Tree | Likely Native | 12 | 1 | 12 |
| Otu364 | ITS2 | Magnoliopsida | Gentianales | Rubiaceae | Coprosma |  | Shrub or Tree | Likely Native | 15 | 1 | 15 |
| Otu370 | ITS2 | Magnoliopsida | Gentianales | Rubiaceae | Galium |  | Forb | Likely Native | 6 | 1 | 6 |
| Otu371 | ITS2 | Magnoliopsida | Gentianales | Rubiaceae | Nertera | granadensis | Forb | Likely Native | 5 | 1 | 5 |
| Otu372 | ITS2 | Magnoliopsida | Asterales | Asteraceae | Ozothamnus | stirlingii | Shrub or Tree | Likely Native | 60 | 1 | 60 |
| Otu377 | ITS2 | Magnoliopsida | Poales | Poaceae | Rytidosperma |  | Grass | Likely Native | 5 | 1 | 5 |
| Otu379 | ITS2 | Magnoliopsida | Rosales | Rosaceae | Potentilla | indica | Forb | Likely Exotic | 5 | 1 | 5 |
| Otu380 | ITS2 | Magnoliopsida | Asterales | Asteraceae | Senecio | phelleus | Forb | Likely Native | 11 | 1 | 11 |
| Otu381 | ITS2 | Magnoliopsida | Brassicales | Brassicaceae | Brassica | oleracea | Forb | Likely Exotic | 9 | 1 | 9 |
| Otu385 | ITS2 | Magnoliopsida | Asterales | Asteraceae | Cassinia | longifolia | Shrub or Tree | Likely Native | 773 | 8 | 97 |
| Otu387 | ITS2 | Magnoliopsida | Gentianales | Rubiaceae | Galium |  | Forb | Likely Native | 34 | 1 | 34 |
| Otu391 | ITS2 | Magnoliopsida | Saxifragales | Crassulaceae |  |  | Forb | Unknown | 5 | 1 | 5 |
| Otu401 | ITS2 | Magnoliopsida | Gentianales | Rubiaceae | Coprosma |  | Shrub or Tree | Likely Native | 19 | 1 | 19 |
| Otu405 | ITS2 | Magnoliopsida | Lamiales | Plantaginaceae | Veronica |  | Forb | Likely Native | 67 | 1 | 67 |
| Otu406 | ITS2 | Magnoliopsida | Rosales | Rhamnaceae | Pomaderris |  | Shrub or Tree | Likely Native | 10 | 1 | 10 |
| Otu409 | ITS2 | Magnoliopsida | Rosales | Rhamnaceae | Pomaderris |  | Shrub or Tree | Likely Native | 10 | 1 | 10 |
| Otu413 | ITS2 | Magnoliopsida | Asparagales | Asphodelaceae | Dianella |  | Grass-like Plants | Likely Native | 6 | 1 | 6 |
| Otu414 | ITS2 | Magnoliopsida | Rosales | Rhamnaceae | Pomaderris |  | Shrub or Tree | Likely Native | 6 | 1 | 6 |
| Otu416 | ITS2 | Magnoliopsida | Poales | Juncaceae | Juncus |  | Grass-like Plants | Likely Native | 5 | 1 | 5 |
| Otu417 | ITS2 | Magnoliopsida | Rosales | Rosaceae | Acaena |  | Shrub or Tree | Likely Native | 5 | 1 | 5 |
| Otu418 | ITS2 | Magnoliopsida | Caryophyllales | Polygonaceae | Persicaria |  | Forb | Likely Native | 14 | 1 | 14 |
| Otu421 | ITS2 | Magnoliopsida | Ericales | Ericaceae | Rhododendron |  | Shrub or Tree | Unknown | 10 | 1 | 10 |
| Otu422 | ITS2 | Magnoliopsida | Asterales | Goodeniaceae | Goodenia | ovata | Shrub or Tree | Likely Native | 10 | 2 | 5 |
| Otu425 | ITS2 | Magnoliopsida | Lamiales | Plantaginaceae | Veronica | serpyllifolia | Forb | Unknown | 7 | 1 | 7 |
| Otu426 | ITS2 | Magnoliopsida | Gentianales | Rubiaceae |  |  | Forb | Unknown | 6 | 1 | 6 |
| Otu432 | ITS2 | Magnoliopsida | Lamiales | Plantaginaceae | Veronica |  | Forb | Likely Native | 19 | 1 | 19 |
| Otu450 | ITS2 | Magnoliopsida | Lamiales | Plantaginaceae | Plantago |  | Forb | Likely Native | 7 | 1 | 7 |
| Otu452 | ITS2 | Magnoliopsida | Gentianales | Rubiaceae | Coprosma |  | Shrub or Tree | Likely Native | 5 | 1 | 5 |
| Otu453 | ITS2 | Magnoliopsida | Myrtales | Myrtaceae | Leptospermum |  | Shrub or Tree | Likely Native | 7 | 1 | 7 |
| Otu454 | ITS2 | Magnoliopsida | Poales | Poaceae | Phragmites | australis | Grass | Likely Native | 9 | 1 | 9 |
| Otu459 | ITS2 | Magnoliopsida | Rosales | Rosaceae | Malus | pumila | Shrub or Tree | Likely Exotic | 18 | 1 | 18 |
| Otu460 | ITS2 | Magnoliopsida | Asterales | Asteraceae | Cassinia |  | Shrub or Tree | Likely Native | 7 | 1 | 7 |
| Otu462 | ITS2 | Magnoliopsida | Gentianales | Apocynaceae | Parsonsia | brownii | Climber | Likely Native | 9 | 1 | 9 |
| Otu470 | ITS2 | Magnoliopsida | Asterales | Asteraceae |  |  | Shrub or Tree | Unknown | 7 | 1 | 7 |
| Otu474 | ITS2 | Magnoliopsida | Gentianales | Rubiaceae |  |  | Forb | Unknown | 8 | 1 | 8 |
| Otu478 | ITS2 | Magnoliopsida | Gentianales | Rubiaceae | Coprosma |  | Shrub or Tree | Likely Native | 6 | 1 | 6 |
| Otu480 | ITS2 | Magnoliopsida | Gentianales | Rubiaceae | Coprosma |  | Shrub or Tree | Likely Native | 6 | 1 | 6 |
| Otu482 | ITS2 | Magnoliopsida | Gentianales | Rubiaceae |  |  | Forb | Unknown | 44 | 2 | 22 |
| Otu485 | ITS2 | Magnoliopsida | Rosales | Rosaceae | Acaena |  | Forb | Likely Native | 5 | 1 | 5 |
| Otu488 | ITS2 | Magnoliopsida | Asterales | Alseuosmiaceae | Wittsteinia | vacciniacea | Shrub or Tree | Likely Native | 19 | 2 | 10 |
| Otu492 | ITS2 | Magnoliopsida | Asterales | Asteraceae | Senecio |  | Forb | Likely Native | 49 | 3 | 16 |
| Otu503 | ITS2 | Magnoliopsida | Rosales | Rhamnaceae | Pomaderris |  | Shrub or Tree | Likely Native | 12 | 1 | 12 |
| Otu508 | ITS2 | Magnoliopsida | Rosales | Ulmaceae | Ulmus | procera | Shrub or Tree | Likely Exotic | 12 | 2 | 6 |
| Otu511 | ITS2 | Magnoliopsida | Rosales | Rosaceae |  |  | Shrub or Tree | Unknown | 12 | 2 | 6 |
| Otu516 | ITS2 | Magnoliopsida | Caryophyllales | Polygonaceae | Rumex |  | Forb | Likely Exotic | 57 | 3 | 19 |
| Otu518 | ITS2 | Magnoliopsida | Asterales | Asteraceae | Euchiton |  | Forb | Likely Native | 64 | 1 | 64 |
| Otu520 | ITS2 | Magnoliopsida | Fabales | Fabaceae | Goodia | lotifolia | Shrub or Tree | Likely Native | 17 | 1 | 17 |
| Otu530 | ITS2 | Magnoliopsida | Poales | Poaceae | Paspalum | dilatatum | Grass | Likely Exotic | 26 | 2 | 13 |
| Otu547 | ITS2 | Magnoliopsida | Gentianales | Rubiaceae | Coprosma |  | Shrub or Tree | Likely Native | 5 | 1 | 5 |
| Otu567 | ITS2 | Magnoliopsida | Ericales | Ericaceae | Rhododendron |  | Shrub or Tree | Unknown | 5 | 1 | 5 |
| Otu569 | ITS2 | Magnoliopsida | Poales | Poaceae |  |  | Grass | Unknown | 6 | 1 | 6 |
| Otu572 | ITS2 | Magnoliopsida | Gentianales | Rubiaceae | Coprosma |  | Shrub or Tree | Likely Native | 7 | 1 | 7 |
| Otu575 | ITS2 | Magnoliopsida | Poales | Poaceae | Bromus | catharticus | Grass | Likely Exotic | 14 | 1 | 14 |
| Otu585 | ITS2 | Magnoliopsida | Fabales | Fabaceae | Trifolium |  | Forb | Likely Exotic | 8 | 1 | 8 |
| Otu595 | ITS2 | Magnoliopsida | Poales | Poaceae |  |  | Grass | Unknown | 22 | 1 | 22 |
| Otu598 | ITS2 | Magnoliopsida | Rosales | Rosaceae | Acaena |  | Forb | Likely Native | 5 | 1 | 5 |
| Otu605 | ITS2 | Magnoliopsida | Gentianales | Rubiaceae |  |  | Shrub or Tree | Unknown | 6 | 1 | 6 |
| Otu616 | ITS2 | Magnoliopsida | Gentianales | Rubiaceae |  |  | Shrub or Tree | Unknown | 7 | 1 | 7 |
| Otu635 | ITS2 | Magnoliopsida | Gentianales | Rubiaceae |  |  | Shrub or Tree | Unknown | 10 | 1 | 10 |
| Otu639 | ITS2 | Magnoliopsida | Fabales | Fabaceae | Lotus |  | Forb | Likely Exotic | 36 | 4 | 9 |
| Otu643 | ITS2 | Magnoliopsida | Gentianales | Rubiaceae |  |  | Shrub or Tree | Unknown | 5 | 1 | 5 |
| Otu668 | ITS2 | Magnoliopsida | Fabales | Fabaceae | Glycine |  | Climber | Likely Native | 9 | 1 | 9 |
| Otu670 | ITS2 | Magnoliopsida | Gentianales | Rubiaceae |  |  | Shrub or Tree | Unknown | 9 | 1 | 9 |
| Otu680 | ITS2 | Magnoliopsida | Gentianales | Rubiaceae |  |  | Shrub or Tree | Unknown | 7 | 1 | 7 |
| Otu683 | ITS2 | Magnoliopsida | Fagales | Fagaceae | Quercus |  | Shrub or Tree | Likely Exotic | 497 | 1 | 497 |
| Otu695 | ITS2 | Magnoliopsida | Apiales | Araliaceae |  |  | Shrub or Tree | Unknown | 17 | 1 | 17 |
| Otu715 | ITS2 | Magnoliopsida | Poales | Poaceae | Eragrostis | brownii | Grass | Likely Native | 6 | 1 | 6 |
| Otu722 | ITS2 | Magnoliopsida | Ericales | Ericaceae | Epacris |  | Shrub or Tree | Likely Native | 6 | 1 | 6 |
| Otu738 | ITS2 | Magnoliopsida | Rosales | Rhamnaceae | Pomaderris |  | Shrub or Tree | Likely Native | 353 | 4 | 88 |
| Otu763 | ITS2 | Magnoliopsida | Rosales | Rhamnaceae | Pomaderris |  | Shrub or Tree | Likely Native | 12 | 1 | 12 |
| Otu776 | ITS2 | Magnoliopsida | Rosales | Rosaceae |  |  | Shrub or Tree | Unknown | 5 | 1 | 5 |
| Otu787 | ITS2 | Magnoliopsida | Lamiales | Lamiaceae | Mentha |  | Forb | Likely Native | 6 | 1 | 6 |
| Otu799 | ITS2 | Magnoliopsida | Gentianales | Rubiaceae | Coprosma |  | Shrub or Tree | Likely Native | 7 | 1 | 7 |
| Otu802 | ITS2 | Magnoliopsida | Geraniales | Geraniaceae | Geranium |  | Forb | Likely Native | 68 | 3 | 23 |
| Otu805 | ITS2 | Magnoliopsida | Fagales | Fagaceae | Quercus |  | Shrub or Tree | Likely Exotic | 9 | 1 | 9 |
| Otu810 | ITS2 | Magnoliopsida | Solanales | Convulvulaceae | Ipomoea | indica | Forb | Likely Exotic | 6 | 1 | 6 |
| Otu854 | ITS2 | Magnoliopsida | Saxifragales | Haloragaceae |  |  | Forb | Unknown | 5 | 1 | 5 |
| Otu857 | ITS2 | Magnoliopsida | Gentianales | Rubiaceae |  |  | Shrub or Tree | Unknown | 5 | 1 | 5 |
| Otu865 | ITS2 | Magnoliopsida | Gentianales | Rubiaceae |  |  | Shrub or Tree | Unknown | 5 | 1 | 5 |
| Otu868 | ITS2 | Magnoliopsida | Asterales | Asteraceae | Senecio |  | Forb | Likely Native | 7 | 1 | 7 |
| Otu874 | ITS2 | Magnoliopsida | Poales | Poaceae | Microlaena |  | Grass | Likely Native | 7 | 1 | 7 |
| Otu877 | ITS2 | Magnoliopsida | Ericales | Primulaceae | Lysimachia | arvensis | Forb | Likely Exotic | 5 | 1 | 5 |
| Otu887 | ITS2 | Magnoliopsida | Dipsacales | Caprifoliaceae | Sambucus | gaudichaudiana | Shrub or Tree | Likely Native | 6 | 1 | 6 |
| Otu912 | ITS2 | Magnoliopsida | Rosales | Rhamnaceae | Pomaderris |  | Shrub or Tree | Likely Native | 37 | 1 | 37 |
| Otu917 | ITS2 | Magnoliopsida | Poales | Cyperaceae | Carex | fascicularis | Grass-like Plants | Likely Native | 17 | 1 | 17 |
| Otu1 | rbcLFWD | Magnoliopsida | Canellales | Winteraceae | Tasmannia |  | Shrub or Tree | Likely Native | 204630 | 12 | 17053 |
| Otu1 | rbcLREV | Magnoliopsida | Canellales | Winteraceae | Tasmannia |  | Shrub or Tree | Likely Native | 193814 | 12 | 16151 |
| Otu2 | rbcLFWD | Magnoliopsida | Poales | Poaceae | Ehrharta | erecta | Grass | Likely Exotic | 61033 | 17 | 3590 |
| Otu2 | rbcLREV | Magnoliopsida | Poales | Poaceae |  |  | Grass | Unknown | 51912 | 17 | 3054 |
| Otu3 | rbcLFWD | Magnoliopsida | Poales | Poaceae | Anthoxanthum | odoratum | Grass | Likely Exotic | 64389 | 18 | 3577 |
| Otu3 | rbcLREV | Magnoliopsida | Poales | Poaceae | Anthoxanthum | odoratum | Grass | Likely Exotic | 48657 | 17 | 2862 |
| Otu4 | rbcLFWD | Magnoliopsida | Rosales | Rosaceae | Rubus |  | Shrub or Tree | Likely Exotic | 41524 | 16 | 2595 |
| Otu4 | rbcLREV | Magnoliopsida | Rosales | Rosaceae | Rubus |  | Shrub or Tree | Likely Exotic | 34154 | 16 | 2135 |
| Otu5 | rbcLFWD | Magnoliopsida | Poales | Juncaceae | Juncus | bulbosus | Grass-like Plants | Likely Exotic | 24896 | 3 | 8299 |
| Otu5 | rbcLREV | Polypodiopsida | Polypodiales | Dennstaedtiaceae | Pteridium | esculentum | Fern | Likely Native | 15881 | 12 | 1323 |
| Otu6 | rbcLFWD | Polypodiopsida | Polypodiales | Dennstaedtiaceae | Pteridium | esculentum | Fern | Likely Native | 17075 | 12 | 1423 |
| Otu6 | rbcLREV | Magnoliopsida | Poales | Juncaceae | Juncus | bulbosus | Grass-like Plants | Likely Exotic | 22692 | 3 | 7564 |
| Otu7 | rbcLFWD | Magnoliopsida | Saxifragales | Haloragaceae | Myriophyllum |  | Forb | Likely Native | 20365 | 11 | 1851 |
| Otu7 | rbcLREV | Magnoliopsida | Saxifragales | Haloragaceae | Myriophyllum |  | Forb | Likely Native | 17813 | 11 | 1619 |
| Otu8 | rbcLFWD | Polypodiopsida | Polypodiales | Blechnaceae | Blechnum |  | Fern | Likely Native | 14007 | 7 | 2001 |
| Otu8 | rbcLREV | Magnoliopsida | Gentianales | Rubiaceae | Coprosma |  | Shrub or Tree | Likely Native | 9532 | 11 | 867 |
| Otu9 | rbcLFWD | Polypodiopsida | Cyatheales | Cyatheaceae | Cyathea |  | Fern | Likely Native | 9413 | 10 | 941 |
| Otu9 | rbcLREV | Polypodiopsida | Cyatheales | Cyatheaceae | Cyathea | australis | Fern | Likely Native | 7842 | 10 | 784 |
| Otu10 | rbcLFWD | Magnoliopsida | Santalales | Santalaceae | Santalum |  | Forb | Likely Exotic | 1137 | 6 | 190 |
| Otu11 | rbcLFWD | Magnoliopsida | Myrtales | Myrtaceae | Eucalyptus |  | Shrub or Tree | Likely Native | 17218 | 15 | 1148 |
| Otu12 | rbcLREV | Magnoliopsida | Myrtales | Myrtaceae | Eucalyptus |  | Shrub or Tree | Likely Native | 13303 | 14 | 950 |
| Otu13 | rbcLFWD | Magnoliopsida | Gentianales | Rubiaceae | Nertera | granadensis | Forb | Likely Native | 10507 | 10 | 1051 |
| Otu13 | rbcLREV | Magnoliopsida | Poales | Cyperaceae | Isolepis |  | Grass-like Plants | Likely Native | 6241 | 8 | 780 |
| Otu14 | rbcLFWD | Magnoliopsida | Geraniales | Geraniaceae | Geranium |  | Forb | Likely Native | 5911 | 10 | 591 |
| Otu14 | rbcLREV | Polypodiopsida | Polypodiales | Blechnaceae | Blechnum | cartilagineum | Fern | Likely Native | 10729 | 8 | 1341 |
| Otu15 | rbcLFWD | Magnoliopsida | Oxalidales | Elaeocarpaceae | Elaeocarpus | reticulatus | Shrub or Tree | Likely Native | 5527 | 5 | 1105 |
| Otu15 | rbcLREV | Magnoliopsida | Oxalidales | Elaeocarpaceae |  |  | Shrub or Tree | Unknown | 4853 | 5 | 971 |
| Otu16 | rbcLFWD | Magnoliopsida | Poales | Cyperaceae | Isolepis |  | Grass-like Plants | Likely Native | 5818 | 3 | 1939 |
| Otu16 | rbcLREV | Pinopsida | Pinales | Pinaceae |  |  | Shrub or Tree | Unknown | 2676 | 4 | 669 |
| Otu17 | rbcLFWD | Magnoliopsida | Poales | Poaceae | Rytidosperma | pallidum | Grass | Likely Native | 3553 | 4 | 888 |
| Otu17 | rbcLREV | Magnoliopsida | Fabales | Fabaceae | Acacia |  | Shrub or Tree | Likely Native | 5236 | 8 | 655 |
| Otu18 | rbcLREV | Magnoliopsida | Geraniales | Geraniaceae | Geranium |  | Forb | Likely Native | 4858 | 9 | 540 |
| Otu19 | rbcLFWD | Magnoliopsida | Rosales | Rosaceae | Acaena | novae-zelandiae | Forb | Likely Native | 3110 | 12 | 259 |
| Otu19 | rbcLREV | Magnoliopsida | Poales | Poaceae | Rytidosperma |  | Grass | Likely Native | 3102 | 4 | 776 |
| Otu20 | rbcLFWD | Magnoliopsida | Malpighiales | Violaceae | Viola |  | Forb | Likely Native | 2636 | 4 | 659 |
| Otu21 | rbcLREV | Magnoliopsida | Rosales | Rosaceae | Acaena | novae-zelandiae | Forb | Likely Native | 3033 | 12 | 253 |
| Otu22 | rbcLFWD | Magnoliopsida | Gentianales | Rubiaceae | Galium |  | Forb | Likely Native | 2872 | 5 | 574 |
| Otu22 | rbcLREV | Magnoliopsida | Malpighiales | Violaceae | Viola |  | Forb | Likely Native | 2310 | 4 | 578 |
| Otu23 | rbcLFWD | Magnoliopsida | Asterales | Asteraceae | Hypochaeris |  | Forb | Likely Exotic | 1501 | 12 | 125 |
| Otu24 | rbcLFWD | Magnoliopsida | Lamiales | Plantaginaceae | Gratiola |  | Forb | Likely Native | 2222 | 5 | 444 |
| Otu24 | rbcLREV | Magnoliopsida | Solanales | Convolvulaceae | Cuscuta |  | Forb | Likely Exotic | 1252 | 1 | 1252 |
| Otu25 | rbcLFWD | Magnoliopsida | Caryophyllales | Polygonaceae | Acetosella | vulgaris | Forb | Likely Exotic | 1401 | 3 | 467 |
| Otu26 | rbcLFWD | Polypodiopsida | Cyatheales | Dicksoniaceae | Dicksonia | antarctica | Fern | Likely Native | 1410 | 9 | 157 |
| Otu26 | rbcLREV | Magnoliopsida | Gentianales | Rubiaceae | Galium |  | Forb | Likely Native | 2465 | 5 | 493 |
| Otu27 | rbcLFWD | Magnoliopsida | Rosales | Rhamnaceae | Spyridium | parvifolium | Shrub or Tree | Likely Native | 1412 | 5 | 282 |
| Otu28 | rbcLREV | Magnoliopsida | Lamiales | Plantaginaceae | Gratiola |  | Forb | Likely Native | 1633 | 1 | 1633 |
| Otu31 | rbcLREV | Polypodiopsida | Cyatheales | Dicksoniaceae | Dicksonia | antarctica | Fern | Likely Native | 1242 | 9 | 138 |
| Otu32 | rbcLFWD | Magnoliopsida | Fabales | Fabaceae | Desmodium |  | Forb | Likely Native | 581 | 3 | 194 |
| Otu32 | rbcLREV | Magnoliopsida | Poales | Poaceae |  |  | Grass | Unknown | 933 | 3 | 311 |
| Otu33 | rbcLREV | Magnoliopsida | Rosales | Rhamnaceae | Spyridium | parvifolium | Shrub or Tree | Likely Native | 1234 | 5 | 247 |
| Otu34 | rbcLREV | Magnoliopsida | Fabales | Fabaceae | Desmodium |  | Forb | Likely Native | 581 | 3 | 194 |
| Otu35 | rbcLREV | Magnoliopsida | Fagales | Fagaceae | Quercus | robur | Shrub or Tree | Likely Exotic | 661 | 1 | 661 |
| Otu36 | rbcLREV | Magnoliopsida | Malpighiales | Hypericaceae | Hypericum |  | Forb | Likely Exotic | 678 | 1 | 678 |
| Otu37 | rbcLFWD | Magnoliopsida | Fabales | Fabaceae | Trifolium | repens | Forb | Likely Exotic | 739 | 1 | 739 |
| Otu38 | rbcLFWD | Magnoliopsida | Ericales | Ericaceae | Rhododendron |  | Shrub or Tree | Likely Exotic | 599 | 2 | 300 |
| Otu39 | rbcLREV | Magnoliopsida | Lamiales | Plantaginaceae | Plantago | lanceolata | Forb | Likely Exotic | 729 | 5 | 146 |
| Otu40 | rbcLFWD | Magnoliopsida | Oxalidales | Cunoniaceae |  |  | Shrub or Tree | Likely Native | 945 | 3 | 315 |
| Otu41 | rbcLFWD | Magnoliopsida | Fagales | Fagaceae | Quercus | robur | Shrub or Tree | Likely Exotic | 661 | 1 | 661 |
| Otu44 | rbcLFWD | Magnoliopsida | Lamiales | Plantaginaceae | Plantago | lanceolata | Forb | Likely Exotic | 757 | 5 | 151 |
| Otu45 | rbcLFWD | Magnoliopsida | Fagales | Nothofagaceae | Nothofagus | cunninghamii | Shrub or Tree | Likely Native | 712 | 2 | 356 |
| Otu45 | rbcLREV | Magnoliopsida | Fagales | Nothofagaceae | Nothofagus | cunninghamii | Shrub or Tree | Likely Native | 633 | 2 | 317 |
| Otu46 | rbcLREV | Magnoliopsida | Ericales | Ericaceae | Epacris |  | Shrub or Tree | Likely Native | 553 | 2 | 277 |
| Otu47 | rbcLREV | Magnoliopsida | Fabales | Fabaceae | Trifolium | repens | Forb | Likely Exotic | 673 | 1 | 673 |
| Otu56 | rbcLREV | Magnoliopsida | Caryophyllales | Polygonaceae | Acetosella | vulgaris | Forb | Likely Exotic | 1036 | 3 | 345 |
| Otu57 | rbcLFWD | Magnoliopsida | Poales | Poaceae |  |  | Grass | Unknown | 419 | 2 | 210 |
| Otu57 | rbcLREV | Bryopsida | Bryales | Bryaceae | Rosulabryum | capillare | Mosses and Liverworts | Unknown | 315 | 3 | 105 |
| Otu58 | rbcLREV | Bryopsida | Hypnales | Hypnaceae | Hypnum | cupressiforme | Mosses and Liverworts | Unknown | 380 | 1 | 380 |
| Otu61 | rbcLFWD | Bryopsida | Hypnales | Hypnaceae | Hypnum | curvifolium | Mosses and Liverworts | Unknown | 422 | 2 | 211 |
| Otu62 | rbcLREV | Magnoliopsida | Apiales | Araliaceae | Hydrocotyle |  | Forb | Likely Native | 569 | 2 | 285 |
| Otu70 | rbcLFWD | Magnoliopsida | Laurales | Monimiaceae | Hedycarya | angustifolia | Shrub or Tree | Likely Native | 341 | 4 | 85 |
| Otu70 | rbcLREV | Magnoliopsida | Oxalidales | Cunoniaceae | Bauera | rubioides | Shrub or Tree | Likely Native | 641 | 3 | 214 |
| Otu71 | rbcLFWD | Magnoliopsida | Fabales | Fabaceae | Acacia |  | Shrub or Tree | Likely Native | 6530 | 8 | 816 |
| Otu71 | rbcLREV | Magnoliopsida | Lamiales | Lamiaceae | Prostanthera |  | Shrub or Tree | Likely Native | 257 | 3 | 86 |
| Otu73 | rbcLFWD | Magnoliopsida | Myrtales | Onagraceae | Epilobium |  | Forb | Likely Native | 155 | 2 | 78 |
| Otu80 | rbcLFWD | Magnoliopsida | Asterales | Asteraceae |  |  | Shrub or Tree | Unknown | 541 | 9 | 60 |
| Otu81 | rbcLREV | Magnoliopsida | Lamiales | Plantaginaceae | Callitriche | stagnalis | Forb | Likely Exotic | 226 | 1 | 226 |
| Otu86 | rbcLFWD | Magnoliopsida | Rosales | Urticaceae | Australina | pusilla | Forb | Likely Native | 431 | 4 | 108 |
| Otu86 | rbcLREV | Magnoliopsida | Myrtales | Onagraceae | Epilobium |  | Forb | Likely Native | 151 | 2 | 76 |
| Otu88 | rbcLREV | Magnoliopsida | Santales | Loranthaceae | Muellerina | eucalyptoides | Shrub or Tree | Likely Native | 138 | 1 | 138 |
| Otu92 | rbcLREV | Magnoliopsida | Solanales | Convolvulaceae | Cuscuta | tasmanica | Forb | Likely Native | 142 | 1 | 142 |
| Otu94 | rbcLFWD | Magnoliopsida | Ericales | Primulaceae | Myrsine | howittiana | Forb | Likely Native | 179 | 1 | 179 |
| Otu96 | rbcLREV | Magnoliopsida | Ranunculales | Ranunculaceae | Clematis |  | Forb | Likely Native | 116 | 1 | 116 |
| Otu97 | rbcLREV | Magnoliopsida | Asterales | Campanulaceae | Wahlenbergia | gloriosa | Forb | Likely Native | 29 | 2 | 15 |
| Otu100 | rbcLREV | Magnoliopsida | Geraniales | Geraniaceae | Erodium |  | Forb | Likely Exotic | 157 | 1 | 157 |
| Otu101 | rbcLREV | Magnoliopsida | Rosales | Urticaceae | Urtica | incisa | Forb | Likely Native | 45 | 1 | 45 |
| Otu103 | rbcLFWD | Magnoliopsida | Caryophyllales | Caryophyllaceae | Cerastium | glomeratum | Forb | Likely Exotic | 16 | 1 | 16 |
| Otu103 | rbcLREV | Polypodiopsida | Polypodiales | Dennstaedtiaceae | Histiopteris | incisa | Fern | Likely Native | 147 | 2 | 74 |
| Otu104 | rbcLFWD | Magnoliopsida | Asterales | Campanulaceae | Wahlenbergia | gloriosa | Forb | Likely Native | 28 | 2 | 14 |
| Otu108 | rbcLFWD | Magnoliopsida | Poales | Cyperaceae | Lepidosperma | tortuosum | Grass-like Plants | Likely Native | 1188 | 6 | 198 |
| Otu108 | rbcLREV | Magnoliopsida | Rosales | Urticaceae | Australina | pusilla | Forb | Likely Native | 273 | 3 | 91 |
| Otu110 | rbcLFWD | Magnoliopsida | Ranunculales | Ranunculaceae | Clematis | aristata | Forb | Likely Native | 109 | 1 | 109 |
| Otu112 | rbcLFWD | Magnoliopsida | Solanales | Solanaceae | Solanum |  | Shrub or Tree | Likely Exotic | 332 | 2 | 166 |
| Otu112 | rbcLREV | Magnoliopsida | Fabales | Fabaceae |  |  | Shrub or Tree | Unknown | 147 | 3 | 49 |
| Otu113 | rbcLFWD | Polypodiopsida | Polypodiales | Dennstaedtiaceae | Histiopteris | incisa | Fern | Likely Native | 174 | 2 | 87 |
| Otu113 | rbcLREV | Magnoliopsida | Apiales | Pittosporaceae | Pittosporum | undulatum | Shrub or Tree | Likely Native | 205 | 2 | 103 |
| Otu114 | rbcLFWD | Magnoliopsida | Santales | Loranthaceae | Muellerina | eucalyptoides | Shrub or Tree | Likely Native | 143 | 1 | 143 |
| Otu115 | rbcLREV | Magnoliopsida | Myrtales | Lythraceae | Lythrum |  | Forb | Likely Native | 94 | 1 | 94 |
| Otu117 | rbcLFWD | Polypodiopsida | Polypodiales | Blechnaceae | Blechnum | minus | Fern | Likely Native | 111 | 2 | 56 |
| Otu117 | rbcLREV | Magnoliopsida | Gentianales | Rubiaceae |  |  | Forb | Unknown | 104 | 1 | 104 |
| Otu119 | rbcLFWD | Magnoliopsida | Rosales | Rosaceae | Aphanes | arvensis | Forb | Likely Exotic | 15 | 1 | 15 |
| Otu120 | rbcLREV | Magnoliopsida | Ericales | Ericaceae |  |  | Shrub or Tree | Unknown | 61 | 1 | 61 |
| Otu121 | rbcLFWD | Magnoliopsida | Poales | Poaceae | Anthosachne |  | Grass | Likely Native | 126 | 2 | 63 |
| Otu124 | rbcLREV | Bryopsida | Funariales | Funariaceae | Funaria | hygrometrica | Mosses and Liverworts | Unknown | 59 | 1 | 59 |
| Otu127 | rbcLREV | Magnoliopsida | Fabales | Fabaceae |  |  | Shrub or Tree | Unknown | 150 | 1 | 150 |
| Otu130 | rbcLFWD | Pinopsida | Pinales | Cupressaceae | Callitris | glaucophylla | Shrub or Tree | Likely Exotic | 76 | 1 | 76 |
| Otu134 | rbcLFWD | Magnoliopsida | Sapindales | Rutaceae | Zieria |  | Shrub or Tree | Likely Native | 133 | 2 | 67 |
| Otu135 | rbcLREV | Magnoliopsida | Solanales | Convolvulaceae |  |  | Forb | Unknown | 52 | 1 | 52 |
| Otu136 | rbcLREV | Polypodiopsida | Polypodiales | Blechnaceae | Blechnum |  | Fern | Likely Native | 2270 | 7 | 324 |
| Otu137 | rbcLREV | Pinopsida | Pinales | Cupressaceae | Callitris |  | Shrub or Tree | Likely Exotic | 69 | 1 | 69 |
| Otu141 | rbcLREV | Magnoliopsida | Gentianales | Rubiaceae |  |  | Forb | Unknown | 65 | 1 | 65 |
| Otu142 | rbcLFWD | Magnoliopsida | Fabales | Fabaceae | Mirbelia | oxylobioides | Shrub or Tree | Likely Native | 134 | 3 | 45 |
| Otu146 | rbcLREV | Magnoliopsida | Fabales | Fabaceae | Lotus |  | Forb | Likely Exotic | 56 | 2 | 28 |
| Otu147 | rbcLFWD | Magnoliopsida | Asparagales | Asphodelaceae | Dianella | tasmanica | Forb | Likely Native | 47 | 1 | 47 |
| Otu147 | rbcLREV | Magnoliopsida | Solanales | Convolvulaceae | Cuscuta |  | Forb | Likely Exotic | 40 | 1 | 40 |
| Otu148 | rbcLFWD | Magnoliopsida | Cucurbitales | Cucurbitaceae |  |  | Forb | Unknown | 44 | 1 | 44 |
| Otu149 | rbcLFWD | Magnoliopsida | Proteales | Proteaceae | Lomatia |  | Shrub or Tree | Likely Native | 43 | 1 | 43 |
| Otu152 | rbcLREV | Magnoliopsida | Malpighiales | Euphorbiaceae | Amperea | xiphoclada | Shrub or Tree | Likely Native | 64 | 1 | 64 |
| Otu154 | rbcLREV | Magnoliopsida | Caryophyllales | Caryophyllaceae | Cerastium | glomeratum | Forb | Likely Exotic | 10 | 1 | 10 |
| Otu157 | rbcLFWD | Polypodiopsida | Polypodiales | Dennstaedtiaceae | Hypolepis |  | Fern | Likely Native | 94 | 3 | 31 |
| Otu159 | rbcLFWD | Magnoliopsida | Malpighiales | Euphorbiaceae | Amperea | xiphoclada | Shrub or Tree | Likely Native | 61 | 1 | 61 |
| Otu160 | rbcLREV | Bryopsida | Dicranales | Ditrichaceae | Ditrichum |  | Mosses and Liverworts | Unknown | 84 | 1 | 84 |
| Otu161 | rbcLREV | Magnoliopsida | Solanales | Convovulaceae |  |  | Forb | Unknown | 44 | 1 | 44 |
| Otu163 | rbcLREV | Magnoliopsida | Poales | Poaceae |  |  | Grass | Unknown | 39 | 1 | 39 |
| Otu164 | rbcLREV | Magnoliopsida | Poales | Poaceae |  |  | Grass | Unknown | 78 | 1 | 78 |
| Otu165 | rbcLREV | Magnoliopsida | Solanales | Solanaceae | Solanum |  | Shrub or Tree | Likely Exotic | 1871 | 13 | 144 |
| Otu166 | rbcLFWD | Magnoliopsida | Myrtales | Lythraceae | Lythrum |  | Forb | Likely Native | 70 | 1 | 70 |
| Otu167 | rbcLREV | Magnoliopsida | Asparagales | Asphodelaceae | Dianella | tasmanica | Forb | Likely Native | 37 | 1 | 37 |
| Otu168 | rbcLREV | Magnoliopsida | Sapindales | Rutaceae | Zieria | arborescens | Shrub or Tree | Likely Native | 130 | 2 | 65 |
| Otu176 | rbcLREV | Magnoliopsida | Ericales | Primulaceae | Myrsine | howittiana | Shrub or Tree | Likely Native | 112 | 1 | 112 |
| Otu181 | rbcLREV | Magnoliopsida | Proteales | Proteaceae | Persoonia |  | Shrub or Tree | Likely Native | 37 | 1 | 37 |
| Otu182 | rbcLFWD | Bryopsida | Dicranales | Leucobryaceae | Campylopus |  | Mosses and Liverworts | Unknown | 13 | 1 | 13 |
| Otu187 | rbcLFWD | Magnoliopsida | Gentianales | Rubiaceae |  |  | Forb | Unknown | 38 | 1 | 38 |
| Otu188 | rbcLREV | Magnoliopsida | Gentianales | Rubiaceae |  |  | Forb | Unknown | 31 | 1 | 31 |
| Otu189 | rbcLREV | Magnoliopsida | Fabales | Fabaceae | Glycine | tabacina | Forb | Likely Native | 32 | 1 | 32 |
| Otu191 | rbcLREV | Magnoliopsida | Solanales | Convovulaceae |  |  | Forb | Unknown | 22 | 1 | 22 |
| Otu196 | rbcLREV | Magnoliopsida | Poales | Poaceae | Phragmites | australis | Grass | Likely Native | 54 | 1 | 54 |
| Otu199 | rbcLREV | Magnoliopsida | Malpighiales | Salicaceae | Populus | alba | Shrub or Tree | Likely Exotic | 22 | 1 | 22 |
| Otu202 | rbcLREV | Magnoliopsida | Asparagales | Asteliaceae | Astelia |  | Forb | Likely Native | 76 | 1 | 76 |
| Otu208 | rbcLREV | Magnoliopsida | Lamiales | Lamiaceae | Prunella | vulgaris | Forb | Likely Exotic | 31 | 1 | 31 |
| Otu209 | rbcLFWD | Jungermanniopsida | Metzgeriales | Metzgeriaceae | Metzgeria |  | Mosses and Liverworts | Unknown | 47 | 1 | 47 |
| Otu212 | rbcLREV | Jungermanniopsida | Metzgeriales | Metzgeriaceae | Metzgeria |  | Mosses and Liverworts | Unknown | 40 | 1 | 40 |
| Otu213 | rbcLFWD | Magnoliopsida | Malpighiales | Salicaceae | Populus | alba | Shrub or Tree | Likely Exotic | 21 | 1 | 21 |
| Otu214 | rbcLFWD | Magnoliopsida | Cucurbitales | Cucurbitaceae |  |  | Forb | Unknown | 15 | 1 | 15 |
| Otu217 | rbcLFWD | Bryopsida | Funariales | Funariaceae | Funaria | hygrometrica | Mosses and Liverworts | Unknown | 65 | 1 | 65 |
| Otu218 | rbcLREV | Pinopsida | Pinales | Pinaceae | Pinus |  | Shrub or Tree | Likely Exotic | 18 | 1 | 18 |
| Otu219 | rbcLREV | Magnoliopsida | Poales | Juncaceae | Juncus |  | Grass-like Plants | Likely Native | 45 | 1 | 45 |
| Otu228 | rbcLFWD | Pinopsida | Pinales | Cupressaceae | Cupressus | sempervirens | Shrub or Tree | Likely Exotic | 17 | 1 | 17 |
| Otu228 | rbcLREV | Polypodiopsida | Polypodiales | Blechnaceae | Blechnum | cartilagineum | Fern | Likely Native | 20 | 1 | 20 |
| Otu230 | rbcLFWD | Magnoliopsida | Gentianales | Rubiaceae | Coprosma |  | Forb | Likely Native | 34 | 1 | 34 |
| Otu231 | rbcLREV | Magnoliopsida | Gentianales | Rubiaceae |  |  | Shrub or Tree | Unknown | 33 | 1 | 33 |
| Otu233 | rbcLFWD | Magnoliopsida | Poales | Poaceae | Phragmites | australis | Grass | Likely Native | 144 | 2 | 72 |
| Otu240 | rbcLREV | Magnoliopsida | Cucurbitales | Cucurbitaceae |  |  | Forb | Unknown | 34 | 1 | 34 |
| Otu243 | rbcLFWD | Magnoliopsida | Gentianales | Rubiaceae |  |  | Forb | Unknown | 26 | 2 | 13 |
| Otu243 | rbcLREV | Magnoliopsida | Poales | Cyperaceae | Carex |  | Grass-like Plants | Likely Native | 1921 | 4 | 480 |
| Otu245 | rbcLREV | Magnoliopsida | Asterales | Asteraceae | Senecio |  | Forb | Likely Native | 15 | 1 | 15 |
| Otu246 | rbcLREV | Magnoliopsida | Solanales | Convovulaceae |  |  | Forb | Unknown | 15 | 1 | 15 |
| Otu248 | rbcLREV | Magnoliopsida | Gentianales | Rubiaceae |  |  | Forb | Unknown | 26 | 2 | 13 |
| Otu250 | rbcLFWD | Polypodiopsida | Polypodiales | Blechnaceae | Blechnum | fluviatile | Fern | Likely Native | 17 | 1 | 17 |
| Otu260 | rbcLREV | Magnoliopsida | Poales | Poaceae |  |  | Grass | Unknown | 65 | 1 | 65 |
| Otu263 | rbcLFWD | Magnoliopsida | Gentianales | Gentianaceae | Centaurium | erythraea | Forb | Likely Exotic | 12 | 1 | 12 |
| Otu265 | rbcLREV | Polypodiopsida | Polypodiales | Blechnaceae | Blechnum |  | Fern | Likely Native | 21 | 1 | 21 |
| Otu270 | rbcLREV | Magnoliopsida | Poales | Poaceae | Paspalum | dilatatum | Grass | Likely Exotic | 67 | 1 | 67 |
| Otu274 | rbcLREV | Magnoliopsida | Solanales | Convolvulaceae | Cuscuta |  | Forb | Likely Exotic | 28 | 1 | 28 |
| Otu279 | rbcLFWD | Magnoliopsida | Apiales | Apiaceae | Oreomyrrhis | eriopoda | Forb | Likely Native | 13 | 1 | 13 |
| Otu283 | rbcLREV | Magnoliopsida | Proteales | Proteaceae | Lomatia |  | Shrub or Tree | Likely Native | 24 | 1 | 24 |
| Otu288 | rbcLFWD | Magnoliopsida | Poales | Poaceae | Holcus | lanatus | Grass | Likely Exotic | 390 | 8 | 49 |
| Otu289 | rbcLFWD | Polypodiopsida | Gleicheniales | Gleicheniaceae | Gleichenia | dicarpa | Fern | Likely Native | 12 | 1 | 12 |
| Otu291 | rbcLFWD | Magnoliopsida | Poales | Cyperaceae | Carex | buxbaumii | Grass-like Plants | Likely Exotic | 2261 | 5 | 452 |
| Otu294 | rbcLFWD | Polypodiopsida | Polypodiales | Dryopteridaceae | Polystichum |  | Fern | Likely Native | 14 | 1 | 14 |
| Otu296 | rbcLREV | Bryopsida | Dicranales | Leucobryaceae | Campylopus |  | Mosses and Liverworts | Unknown | 28 | 1 | 28 |
| Otu299 | rbcLREV | Magnoliopsida | Laurales | Monimiaceae | Hedycarya | angustifolia | Shrub or Tree | Likely Native | 249 | 3 | 83 |
| Otu300 | rbcLFWD | Magnoliopsida | Poales | Poaceae | Phleum | pratense | Grass | Likely Exotic | 15 | 1 | 15 |
| Otu301 | rbcLREV | Pinopsida | Pinales | Cupressaceae |  |  | Shrub or Tree | Unknown | 15 | 1 | 15 |
| Otu305 | rbcLFWD | Polypodiopsida | Polypodiales | Blechnaceae | Blechnum |  | Fern | Likely Native | 216 | 1 | 216 |
| Otu310 | rbcLREV | Magnoliopsida | Ericales | Ericaceae | Leucopogon |  | Shrub or Tree | Likely Native | 11 | 1 | 11 |
| Otu313 | rbcLREV | Magnoliopsida | Poales | Poaceae | Ehrharta | erecta | Grass | Likely Exotic | 2525 | 2 | 1263 |
| Otu316 | rbcLFWD | Magnoliopsida | Poales | Poaceae |  |  | Grass | Unknown | 2313 | 12 | 193 |
| Otu317 | rbcLFWD | Bryopsida | Bryales | Bryaceae | Bryum |  | Mosses and Liverworts | Unknown | 304 | 3 | 101 |
| Otu319 | rbcLFWD | Magnoliopsida | Poales | Poaceae | Festuca | arundinacea | Grass | Likely Exotic | 261 | 7 | 37 |
| Otu322 | rbcLFWD | Magnoliopsida | Poales | Juncaceae | Juncus |  | Grass-like Plants | Likely Native | 32 | 1 | 32 |
| Otu328 | rbcLFWD | Magnoliopsida | Myrtales | Myrtaceae |  |  | Shrub or Tree | Unknown | 237 | 3 | 79 |
| Otu336 | rbcLFWD | Magnoliopsida | Poales | Poaceae |  |  | Grass | Unknown | 17 | 1 | 17 |
| Otu344 | rbcLREV | Magnoliopsida | Poales | Poaceae |  |  | Grass | Unknown | 25 | 2 | 13 |
| Otu348 | rbcLREV | Magnoliopsida | Poales | Poaceae |  |  | Grass | Unknown | 26 | 1 | 26 |
| Otu349 | rbcLREV | Magnoliopsida | Poales | Poaceae | Holcus | lanatus | Grass | Likely Exotic | 2508 | 14 | 179 |
| Otu353 | rbcLFWD | Magnoliopsida | Poales | Poaceae | Agrostis | capillaris | Grass | Likely Exotic | 358 | 7 | 51 |
| Otu359 | rbcLREV | Polypodiopsida | Polypodiales | Blechnaceae | Blechnum | nudum | Fern | Likely Native | 496 | 6 | 83 |
| Otu364 | rbcLREV | Magnoliopsida | Rosales | Urticaceae |  |  | Forb | Unknown | 94 | 1 | 94 |
| Otu365 | rbcLREV | Magnoliopsida | Poales | Poaceae |  |  | Grass | Unknown | 93 | 2 | 47 |
| Otu367 | rbcLFWD | Bryopsida | Dicranales | Ditrichaceae | Ditrichum |  | Mosses and Liverworts | Unknown | 98 | 1 | 98 |
| Otu374 | rbcLREV | Magnoliopsida | Poales | Poaceae | Ehrharta |  | Grass | Likely Exotic | 50 | 1 | 50 |
| Otu379 | rbcLFWD | Polypodiopsida | Polypodiales | Blechnaceae | Blechnum |  | Fern | Likely Native | 2400 | 6 | 400 |
| Otu386 | rbcLFWD | Magnoliopsida | Asterales | Asteraceae |  |  | Shrub or Tree | Unknown | 584 | 1 | 584 |
| Otu389 | rbcLFWD | Magnoliopsida | Fabales | Fabaceae | Glycine | tabacina | Forb | Likely Native | 34 | 1 | 34 |
| Otu390 | rbcLFWD | Magnoliopsida | Asterales | Asteraceae |  |  | Shrub or Tree | Unknown | 271 | 1 | 271 |
| Otu391 | rbcLFWD | Magnoliopsida | Asterales | Stylidiaceae | Stylidium | graminifolium | Forb | Likely Native | 10 | 1 | 10 |
| Otu392 | rbcLFWD | Magnoliopsida | Poales | Poaceae | Polypogon |  | Grass | Likely Exotic | 84 | 3 | 28 |
| Otu407 | rbcLFWD | Magnoliopsida | Saxifragales | Haloragaceae | Myriophyllum |  | Forb | Likely Native | 12 | 1 | 12 |
| Otu416 | rbcLFWD | Magnoliopsida | Poales | Poaceae | Agrostis |  | Grass | Likely Native | 72 | 1 | 72 |
| Otu419 | rbcLFWD | Magnoliopsida | Gentianales | Rubiaceae | Galium |  | Forb | Likely Native | 568 | 1 | 568 |
| Otu434 | rbcLFWD | Polypodiopsida | Polypodiales | Blechnaceae | Blechnum | nudum | Fern | Likely Native | 837 | 7 | 120 |
| Otu442 | rbcLFWD | Polypodiopsida | Polypodiales | Blechnaceae | Blechnum |  | Fern | Likely Native | 74 | 3 | 25 |
| Otu449 | rbcLFWD | Magnoliopsida | Fabales | Fabaceae |  |  | Shrub or Tree | Unknown | 42 | 1 | 42 |
| Otu452 | rbcLFWD | Magnoliopsida | Poales | Poaceae |  |  | Grass | Unknown | 210 | 4 | 53 |
| Otu453 | rbcLFWD | Magnoliopsida | Rosales | Rosaceae | Rubus |  | Shrub or Tree | Likely Exotic | 14 | 1 | 14 |
| Otu466 | rbcLFWD | Magnoliopsida | Gentianales | Rubiaceae | Galium |  | Forb | Likely Native | 13 | 1 | 13 |
| Otu477 | rbcLFWD | Magnoliopsida | Poales | Poaceae | Avena |  | Grass | Likely Exotic | 14 | 1 | 14 |
| Otu490 | rbcLFWD | Magnoliopsida | Asterales | Asteraceae |  |  | Shrub or Tree | Unknown | 294 | 1 | 294 |
| Otu503 | rbcLFWD | Magnoliopsida | Fabales | Fabaceae | Lotus |  | Forb | Likely Exotic | 60 | 2 | 30 |
| Otu518 | rbcLFWD | Bryopsida | Grimmiales | Grimmiaceae | Schistidium |  | Mosses and Liverworts | Unknown | 27 | 1 | 27 |
| Otu523 | rbcLFWD | Magnoliopsida | Poales | Juncaceae | Juncus |  | Grass-like Plants | Likely Native | 432 | 1 | 432 |
| Otu526 | rbcLFWD | Magnoliopsida | Asterales | Asteraceae |  |  | Shrub or Tree | Unknown | 113 | 1 | 113 |
| Otu537 | rbcLFWD | Polypodiopsida | Polypodiales | Blechnaceae | Blechnum |  | Fern | Likely Native | 12 | 1 | 12 |
| Otu546 | rbcLFWD | Magnoliopsida | Asterales | Asteraceae |  |  | Shrub or Tree | Unknown | 56 | 1 | 56 |
| Otu553 | rbcLFWD | Polypodiopsida | Polypodiales | Blechnaceae | Blechnum |  | Fern | Likely Native | 431 | 5 | 86 |

**Table S5.** Estimated mean number of seeds dispersed by sambar deer in both the Alpine National Park (Alpine) and Yarra Ranges National Park (Wet Forest), based on vegetation communities that faecal samples were collected from.
